# Alternative methods for pharmacological research on the action mechanisms of natural products used in the treatment of type 2 diabetes: a systematic review

**DOI:** 10.3389/fphar.2026.1729030

**Published:** 2026-02-10

**Authors:** Fernanda Artemisa Espinoza-Hernández, Samantha Martínez-Medina, Gerardo Mata-Torres, Christian Alan Cabello-Hernández, Adolfo Andrade-Cetto

**Affiliations:** 1 Laboratorio de Etnofarmacología, Departamento de Biología Celular, Facultad de Ciencias, Universidad Nacional Autónoma de México, Ciudad Universitaria, Ciudad de México, Mexico; 2 Posgrado en Ciencias Biológicas, Unidad de Posgrado, Ciudad Universitaria, Ciudad de México, Mexico; 3 Bioterio, Departamento de Biología Celular, Facultad de Ciencias, Universidad Nacional Autónoma de México, Ciudad Universitaria, Ciudad de México, Mexico

**Keywords:** 4Rs, animal research, bioethics, ethnopharmacology, in vitro studies, natural products, type 2 diabetes

## Abstract

Type 2 diabetes (T2D) is a complex metabolic disorder characterized by alterations in multiple pathways of carbohydrate and lipid metabolism. Due to the involvement of a network of dysfunctional enzymes contributing to hyperglycemia, ethnopharmacological research has increasingly focused on identifying new drugs that can improve clinical outcomes. In this context, animal models have remained essential for evaluating naturally occurring molecules with potential hypoglycemic effects. However, the scientific community has recently emphasized the need for alternatives to animal use in biomedical research. This review aims to highlight the alternative approaches employed in recent years to discover natural products with therapeutic potential for T2D, emphasizing the most relevant mechanisms of action and pharmacological targets. A systematic search of original articles published between 2019 and 2024 in the PubMed, Scopus, and Web of Science databases identified nine key mechanisms: inhibition of carbohydrate breakdown, modulation of glucose absorption and uptake, enhancement of glucose storage, suppression of glucose production, targeting insulin resistance factors, insulin sensitization, β-cell protection, improvement of lipid metabolism, and sirtuin modulation. During this period, approximately 45% of the studies employed predominantly *in vitro* approaches involving enzymes, transporters, and receptors central to the pathophysiology of T2D, while *in silico* studies displaced *in vivo* studies, increasing their percentage from 16% to 36% in the last year. This review presents a new classification of action mechanisms and compiles the most representative types of assays that have been carried out in recent years to address the most studied pharmacological targets. Therefore, it is expected that this review serves as a foundation for future investigations into underexplored mechanisms particularly insulin sensitization and pancreatic β-cell preservation that may offer greater therapeutic impact.

## 1 Introduction

As science advances, experimental techniques and designs evolve, are corrected and refined. Animal experimentation has led to an awareness of the bioethical implications surrounding their use as experimental subjects, mainly in biomedical research. The importance of ethical practice in scientific activity not only allows for the proper treatment of experimental animals, but also enriches scientific practice and avoids compromising experimental results. Some considerations are a clear and delimited justification for their use; avoid the unnecessary use of animals (i.e., use the smallest possible sample size that has sufficient statistical power to obtain reliable results); training of personnel in charge of animal management; and consideration of environmental and enrichment needs of animals. These questions have resulted in the development of 4Rs within experimental bioethics: reduction, refinement, replacement, and responsibility (Liu et al., 2025).

The search for alternatives to animal testing has become more relevant in recent years. These methods include any options that may replace, reduce, or refine the use of animals in biomedical research or teaching. Some sectors of the scientific community agree that animal testing should be allowed where no viable alternatives exist and provided it is conducted under the bioethical guidelines proposed by the 4Rs. In this context, the interconnectedness between human health and nature that governs the ethnopharmacological view does not make it indifferent to the questions about the use of animal models, since they have been a key tool in the assessment of the pharmacological effect of various bioactive agents from natural sources (Busia, 2024).

Particularly, the use of medicinal plants in the treatment of diabetes has been documented in various parts of the world throughout ethnopharmacological studies, whose research has led to the discovery and description of the action mechanisms behind their pharmacological effects. Diabetes is now considered one of the major epidemics of this century, affecting 588.7 million people worldwide and continuously exceeding its prevalence projections. Its impact on patients’ health is well documented, where type 2 diabetes (T2D) plays a crucial role in the development of complications and comorbidities responsible for reducing their lifespan and quality of life (International Diabetes Federation, 2025).

T2D can be defined as a group of metabolic disorders characterized by hyperglycemia, which is the result of β-cell dysfunction in a context of insulin resistance (American Diabetes Association Professional Practice Committee, 2025). Insulin malfunction, a consequence of disturbances in lipid metabolism, is the core problem that leads to the development of the pathophysiological abnormalities affecting glucose homeostasis that include an exacerbated production of endogenous glucose, a decrease in glucose uptake and storage, a deterioration of pancreatic β cells that impairs insulin secretion, a diminished incretin effect, and an increased renal glucose reabsorption (DeFronzo et al., 2015). To counteract the high glucose levels resulting from these abnormalities, there is an arsenal of drugs available; nevertheless, there is a need to continue research and improve therapies. Therefore, both *in vivo* and *in vitro* studies have been designed to explore the mechanisms of action behind their pharmacological effects. Because of the traditional approach involving the oral administration route, the need to use animal models to simulate this route has become indispensable. However, considering the growing diabetes epidemic and the need to design new drug therapies, it is necessary to find alternatives that involve the fewest *in vivo* experimental designs and that evaluate relevant drug targets in the disease. Consequently, based on self-assessment and need, this review seeks to contribute to a compilation of alternative methods for ethnopharmacological research of T2D, as well as the most evaluated pharmacological targets, by reviewing the most recent research that has been carried out with natural products in the last 5 years.

## 2 Materials and methods

A literature search was carried out on 19 December 2024 based on the guidelines of the Preferred Reporting Items for Systematic Review and Meta-Analysis (PRISMA) (Page et al., 2021) in PubMed, Scopus, and Web of Science (Supplementary Figure S1). The search terms used were “type 2 diabetes” and, “medicinal plants” or “natural products”, and “*in vivo*” or “*in vitro*” or “*in silico*”. For each database, the search was refined using the following filters: original articles, articles in English, and articles published from 2019 to 2024. The articles returned by the search of each database were condensed into a table, where duplicates were first removed. The resulting articles were screened using as an inclusion criterion those that evaluated a natural product or a medicinal plant on a specific pharmacological target or on metabolic parameters directly related to diabetes, such as glucose or lipid profile. Articles that were not research (reviews), unrelated to diabetes, that evaluated any compound in humans (clinical tries), that only evaluated parameters of inflammation or oxidative stress, that only evaluated the complications of diabetes, that only provided ethnobotanical data, and that did not evaluate any medicinal plant or natural product were excluded. Subsequently, each article was reviewed to identify the pharmacological target(s) evaluated, as well as the type of approach employed. These were classified as *in vivo* (experiments performed on living organisms), *in vitro* (experiments performed directly with enzymes or cellular components), *in silico* (experiments performed using computer simulation with specialized software), and *ex vivo* (experiments performed on organs or tissues after they have been removed from living organisms. Three people participated in the screening process and any disagreement about whether to include the article or not was discussed. Since the objective of this review was to compile existing evidence on current methods used rather than to evaluate the effects or causality of the methodologies employed, a formal assessment of the risk of bias was not performed. The resulting references grouped by mechanism can be found in the Supplementary Table S1.

## 3 Results

### 3.1 Overview

**FIGURE 1:**
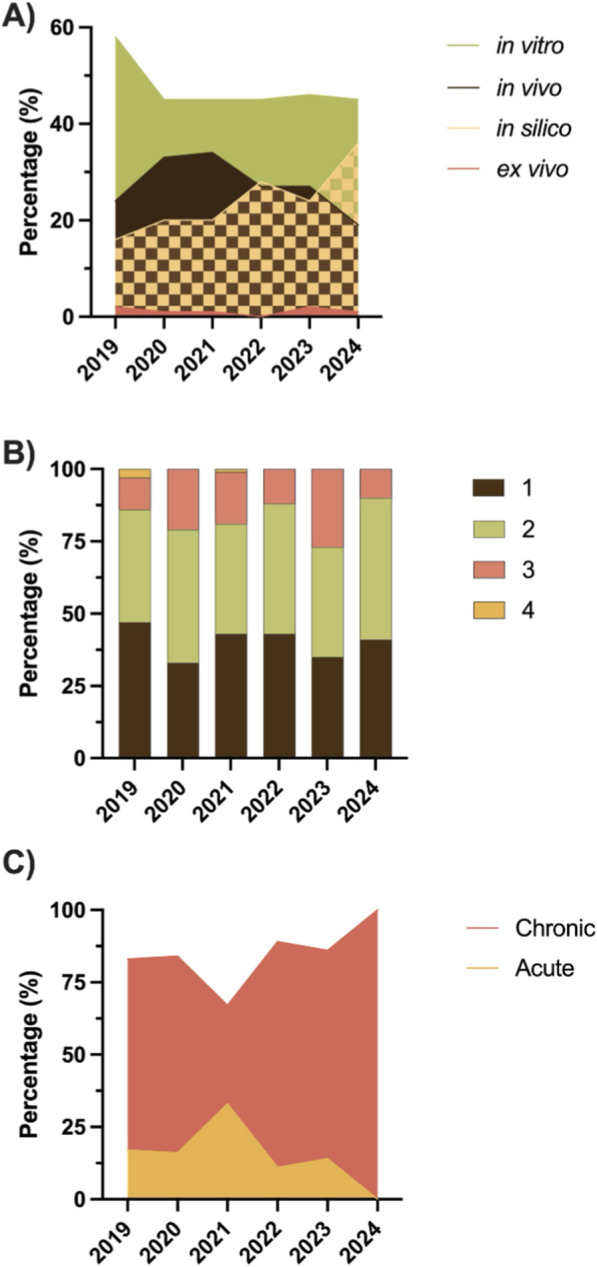
Main findings of the systematic review. **(A)** Percentage of experimental studies by year. A predominance of *in vitro* studies has been observed since 2019; on the other hand, while *in vivo* studies have been declining, *in silico* studies have been increasing. **(B)** Number of experimental studies per publication. There is a clear trend that studies involve one to two experimental approaches to studying the effect of natural products in the treatment of diabetes. **(C)** Experimental design. Most *in vivo* studies involve a chronic experimental design, where the effect of natural products on long-term metabolic parameters is evaluated.

Four hundred and sixty-six articles were selected to perform the analysis by experimental approach and by pharmacological targets grouped into mechanisms. According to the outcomes, it can be recognized four types of approaches regarding the evaluation of the potential therapeutical effect of natural products on T2D: *in vivo*, *ex vivo*, *in vitro*, and *in silico* studies. Figure 1A shows the percentage distribution of publications by study type over the past 5 years. A predominance of *in vitro* assays is observed during this period, with a percentage close to 45% that has remained stable since 2020. Meanwhile, *in vivo* studies have been displaced by *in silico* studies, especially in the last year; the latter have shown an upward trend over the past 5 years, with a percentage that has increased from 16% in 2019 to 36% in 2024. Finally, *ex vivo* assays represent the smallest component analyzed, accounting for only 1%–2% of the total publications.

Throughout the analyzed period, most publications had one or two types of studies, representing between 73% and 90% of the total published articles; publications with two studies reached a maximum of 49% of the total published articles in 2024. On the other hand, publications with three studies ranged from a low of 11% in 2019 to a high of 27% in 2023, with no clear trend. Publications with four assays were rare, with no articles of this type having been reported in the last 3 years (Figure 1B). In the case of *in vivo* assays, Figure 1C shows how many studies were chronic or acute according to the percentage they represented of the total per year. In 2024, 100% of the published studies were chronic, which is consistent with their increase over the last 5 years.

Overall, the main animal model that has been used to evaluate the effect of natural products *in vivo* is that induced by a high-fat diet and streptozotocin, whose characteristics and origin resemble the natural history of T2D. This model is used to characterize the impact of molecules or plant extracts on metabolic parameters such as glucose, glycated hemoglobin, and lipid profile, as well as specific marker proteins. Usually, the natural product is administered once to evaluate its acute effect or several times over a sustained period to assess its long-term impact. On the other hand, the most used *in vitro* method is enzyme assays, which evaluate the potential inhibitory or activating effect of enzymes relevant to a specific pharmacological target, while the most used *in silico* technique is molecular docking, which simulates the interaction of a specific molecule to a particular protein target. Finally, *ex vivo* studies involve applying the natural product to isolated cells or tissues of a living organism to assess its effect on a particular target.

### 3.2 Specific mechanisms and therapeutic targets

**FIGURE 2:**
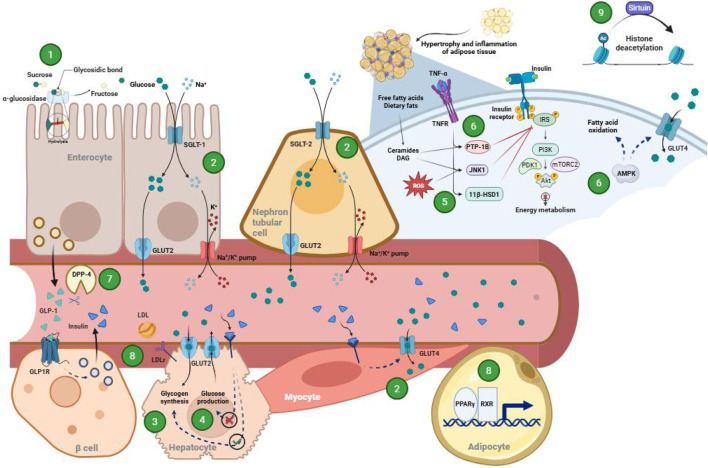
Schematic representation of main targets found through the systematic search: 1) carbohydrate breakdown inhibition, 2) glucose absorption and uptake, 3) glucose storage, 4) glucose production inhibition, 5) insulin resistance driving factors, 6) insulin sensitization, 7) β-cell protection, 8) lipid metabolism improvement, and 9) sirtuin modulation. 11β-HSD1: 11β-hydroxysteroid dehydrogenase type 1; Akt: protein kinase B; AMPK: 5′adenosine monophosphate-activated protein kinase; DAG: diacylglycerol; DPP-4: dipeptidyl peptidase 4; GLP-1: glucagon-like peptide-1; GLP1R: glucagon-like peptide-1 receptor; GLUT-2: glucose transporter type 2; GLUT-4: glucose transporter type 4; IRS: insulin receptor substrate; JNK1: c-Jun N-terminal kinase 1; LDL: low-density lipoprotein; LDLr: low-density lipoprotein receptor; mTORC2: mammalian target of rapamycin complex 2; PDK1: 3-phosphoinositide-dependent protein kinase 1; PI3K: phosphatidylinositol 3-kinase; PPARγ: peroxisome proliferator-activated receptor γ; PTP-1B: protein tyrosine phosphatase 1B; ROS: reactive oxygen species; RXR: retinoid X receptor; SGLT-1: sodium-glucose cotransporter type 1; SGLT-2: sodium-glucose cotransporter type 2; TNF-α: tumor necrosis factor α; TNFR: tumor necrosis factor receptor. Created with BioRender.

For the last 5 years, scientific efforts were made to investigate molecules from natural origin with hypoglycemic properties. Through *in vivo*, *ex vivo*, *in vitro*, and *in silico* approaches, studies have been focused on nine different mechanisms to assess how natural products used traditionally to treat T2D can exert their protective and therapeutic effects on metabolism (Figure 2): 1) carbohydrate breakdown inhibition, 2) glucose absorption and uptake, 3) glucose storage, 4) glucose production inhibition, 5) insulin resistance driving factors, 6) insulin sensitization, 7) β-cell protection, 8) lipid metabolism improvement, and 9) sirtuin modulation. Inhibition of carbohydrate breakdown represents the most addressed mechanism in recent years, encompassing 36.5% of the studies (209 articles) of all the mechanisms found. Although the enzymes involved in carbohydrate hydrolysis are not affected in T2D, inhibiting their activity decreases postprandial hyperglycemia, thereby reducing the hyperinsulinemic peak that negatively impacts the function of pancreatic β cells. The next most studied mechanisms are the protection of β cells and insulin resistance, comprising 13.1% (75 articles) and 11% (63 articles) of the reviewed studies, respectively. Both mechanisms are considered the main pharmacological targets of T2D because restoring the functionality of β cells and improving insulin function implies better regulation of glucose homeostasis. Glucose absorption and uptake and lipid metabolism improvement are the next most frequently studied mechanisms, comprising 9.4% (54 articles) and 8% (46 articles) of the analyzed studies. Eliminating glucose through peripheral uptake is one of the most sought-after targets in current therapies, since 80% of postprandial glucose is taken up by skeletal muscle. On the other hand, improving the lipid profile helps to reduce the low-grade inflammatory environment present in the disease and delays the onset of vascular complications. The least studied mechanisms are insulin sensitization, glucose production inhibition, sirtuin modulation, and glucose storage, which comprised 5.4% (31 articles), 2.8% (16 articles), 0.7% (4 articles), and 0.2% (1 article) of the reviewed studies, respectively. At the hepatic level, glucose production is one of the most important mechanisms that generates fasting hyperglycemia due to an exacerbated gluconeogenesis, while glucose storage contributes to the improvement of postprandial hyperglycemia by synthetizing glycogen from glucose. Finally, there were articles where no mechanism was specified, since they involve experiments on the evaluation of the hypoglycemic effect or on general metabolic parameters of the tested natural products (12.9%, 74 articles). It is worth noting that some articles assessed more than one pharmacological target and, therefore, were assigned to more than one mechanism. Table 1 groups the types of experimental approaches for each mechanism, as well as some representative assays. Each mechanism is next discussed in more detail, along with its implications in the pathophysiology of T2D and examples of some natural products.

**TABLE 1:** Number of experimental approaches and representative assays for each mechanism. Most are addressed through an *in vitro* approach.

Mechanism	Approach	Representative assays
Carbohydrate breakdown inhibition	*In vitro* (192) *In vivo* (50) *In silico* (84) *Ex vivo* (5)	* Enzyme activity inhibition assays * Virtual detection by molecular docking (3D-QSAR) * ADMET prediction * Oral disaccharide tolerance tests
Glucose absorption and uptake	*In vitro* (48) *In vivo* (19) *In silico* (9) *Ex vivo* (2)	* Uptake assays in liver (LO-2 cell model, C3A, HEPG2) and muscle cell lines (L6 myocytes) or isolated muscle * Intestinal absorption assays in Caco2 cell lines
Glucose storage	*In vitro* (0) *In vivo* (0) *In silico* (1) *Ex vivo* (0)	* Compound-protein interaction (CPI) network
Glucose production inhibition	*In vitro* (13) *In vivo* (7) *In silico* (5) *Ex vivo* (3)	* Concentration-response inhibition curves * Ensemble docking and molecular dynamics simulation * ADMET methods * Enzymatic activity of liver homogenate * Gene expression by RT-PCR
Insulin resistance driving factors	*In vitro* (40) *In vivo* (56) *In silico* (15) *Ex vivo* (2)	* Enzymatic assay inhibition * 3T3-L1 adipocyte activity assay * Measurement of liver activity in *ob/ob* mice * Measurement of plasma glucose and insulin to calculate the homeostatic index of insulin resistance (HOMA-IR) and the quantitative insulin sensitivity index (QUICKI) in animal models induced with a high-fat diet and streptozotocin
Insulin sensitization	*In vitro* (28) *In vivo* (15) *In silico* (11) *Ex vivo* (0)	* Enzyme activity inhibition assays * Uptake assays in 3T3-L1 adipocytes * Virtual detection by molecular docking * Western blotting analysis * Network pharmacology analysis * Immunostaining for the expression of muscle atrophy markers * Measurement of metabolic parameters in animal models (induced with a high-fat diet and streptozotocin; *db/db*)
β-cell protection	*In vitro* (54) *In vivo* (33) *In silico* (39) *Ex vivo* (1)	* Pancreatic histological and immunohistochemical analysis * Measurement of plasma glucose and insulin to calculate the homeostatic index of insulin resistance (HOMA-IR) and the quantitative insulin sensitivity index (QUICKI) * Evaluation of rat islet function determined by the HOMA-β index
Lipid metabolism improvement	*In vitro* (39) *In vivo* (26) *In silico* (18) *Ex vivo* (0)	* Adipogenic differentiation assays of adipogenic stem cells * Quantification of hepatic triglycerides and free fatty acids * Gene expression by RT-PCR * Western blotting analysis * *In silico* modeling * Measurement of lipid profile in animal models induced with a high-fat diet and streptozotocin * Oleic red O staining tests
Sirtuin modulation	*In vitro* (2) *In vivo* (2) *In silico* (3) *Ex vivo* (0)	* Molecular docking * Molecular dynamics simulation of SIRT1 activators * Enzyme activations assays * Measurement of metabolic parameters in animal models induced with a high-fat diet * Western blotting analysis
Others	*In vitro* (14) *In vivo* (49) *In silico* (26) *Ex vivo* (2)	* Measurement of serum biochemical parameters in animal models with a high-fat diet and streptozotocin * Oral glucose tolerance test (OGTT) * Histopathological analysis

#### 3.2.1 Carbohydrate breakdown inhibition

Usually, food is composed of a mixture of complex carbohydrates, i.e., oligosaccharides and disaccharides that must be hydrolyzed to be absorbed by the intestine. The metabolism of these oligosaccharides and disaccharides is carried out by enzymes known as α-amylases and α-glucosidases, whose action allows the absorption of glucose (Ghani, 2015; Singh et al., 2023). In patients with diabetes, absorption of carbohydrates is not modified; however, inhibiting the action of the enzymes mentioned above may contribute to the reduction of postprandial hyperglycemia (Maffettone et al., 2018). Hence, to assess the potential inhibition of α-amylases and α-glucosidases by promising natural molecules, *in vitro* and *in silico* assays have been performed. For instance, leaf extract of *Dactylorhiza hatagirea* (D.Don) Soó inhibited the α-amylase enzyme with an IC_50_ = 210.82 μg/mL (Alsawalha et al., 2019), while one polysaccharide isolated from *Porphyra* spp. exhibited an enzymatic inhibition of 98.78% with an IC_50_ = 12.72 mg/mL (Zeng et al., 2020). On the other hand, salvianolic acid C isolated from *Salvia miltiorrhiza* Bunge inhibited α-glucosidase enzyme with an IC_50_ = 3.03 µM (Tang et al., 2019). Another natural product tested against α-glucosidase was fisetin, isolated from *Cotinus coggygria* Scop., which showed inhibitory activity exhibiting an IC_50_ = 4.099 × 10^−4^ mM (Shen et al., 2021).

#### 3.2.2 Glucose absorption and uptake

After the breakdown of complex dietary carbohydrates into monosaccharides, simple sugars are absorbed by the small intestine. Glucose absorption plays a pivotal role in regulating blood glucose levels. Later, it is captured to be used as an energy source by the tissues; therefore, the process of glucose uptake is also an important mechanism for maintaining glucose homeostasis. Since glucose is the primary fuel cells need to generate energy, it must be transported across the membrane of most tissue cells to be usable. In this context, transport occurs through protein complexes anchored to the membrane, either by facilitated diffusion in most tissues or by active transport in the gastrointestinal tract and renal tubules (Nakrani et al., 2025). According to the literature, the measurement of transporter inhibition by natural products is performed *in vitro* by quantifying glucose uptake in cell culture. Labeled glucose is added to the cell culture medium, which was previously incubated with the samples. The cells are then lysed, and the captured labeled glucose is quantified by fluorescence. In addition, the change in gene and/or protein expression of transporters can be determined after administration of treatments in *in vivo* or *in vitro* models.

##### 3.2.2.1 Sodium-glucose cotransporters (SGLT)

Family of transporters that actively transport glucose against its concentration gradient by coupling with sodium. The main cotransporters that have gained relevance as pharmacological targets are sodium-glucose cotransporters type 1 and type 2 (SGLT1 and SGLT2) because both participate in the glucose influx into the circulation. SGLT1 is located in the apical membrane of the intestinal epithelium, while SGLT2 is involved in the glucose reabsorption in the renal tubular cells. The development of SGLT inhibitors that delay postprandial hyperglycemia and increase glucose excretion into urine is considered a potentially effective therapeutic strategy to reduce and control blood glucose levels (Sano et al., 2020). Some natural products that have been reported to inhibit these cotransporters are trigonelline, an alkaloid; pterostilbene and andrographolide, phytoconstituents isolated from *Pterocarpus marsupium* Roxb. and *Andrographis paniculata* (Burm.f.) Wall. ex Nees, respectively; neomangiferin, a xanthone; and soy extract (Dound et al., 2021; Singh et al., 2022; Lüersen et al., 2023; Olusola et al., 2024).

##### 3.2.2.2 Glucose transporters (GLUT)

The large family of glucose transporters (GLUTs) is involved in tissue-specific glucose uptake to ensure glycemic control. GLUT2 and GLUT4 are highly relevant, as they play an essential role in controlling the specific flows for each organ involved in the homeostatic regulation of glucose. GLUT2 participates in the processes of glucose-stimulated insulin secretion in pancreatic β cells and in hepatic glucose uptake. On the other hand, GLUT4 mediates insulin-dependent peripheral glucose uptake in muscle and adipose tissue. Both types of transporters have unique characteristics that allow them to participate in glycemic control under certain conditions. Therefore, improving their functioning has been considered one of the main pharmacological targets in the treatment of diabetes and related metabolic conditions (Artasensi et al., 2020; Chadt and Al-Hasani, 2020). Methyl rosmarinate, isolated from *Cordia morelosana* Standl., has shown to increase GLUT4 expression, while extracts of some plants used in traditional Chinese medicine, such as *Saposhnikovia divaricata* (Turcz. ex Ledeb.) Schischk., *Azadirachta indica* A.Juss., *Scrophularia nodosa* L., *Krameria lappacea* (Dombey) Burdet and B.B.Simpson, and *Epilobium angustifolium* L., demonstrated to promote GLUT4 translocation (Giles-Rivas et al., 2020; Stadlbauer et al., 2021).

#### 3.2.3 Glucose storage

Glucose is stored in the liver and muscle tissues of animals in the form of glycogen. Its primary function is the storage and release of energy, and it is essential for maintaining glucose homeostasis. Among other factors, the regulation of glycogen metabolism is controlled by hormones, with insulin being responsible for signaling glucose storage, i.e., glycogen synthesis. Insulin dysregulation can lead to an impaired glycogen metabolism, affecting storage and release of glucose. In individuals with diabetes or insulin resistance, the balance between synthesis and degradation of glycogen is broken, resulting in chronic hyperglycemia. Moreover, impaired glycogen metabolism further perpetuates insulin resistance (Neoh et al., 2024).

##### 3.2.3.1 Glycogen synthase kinase 3 (GSK3)

GSK3 is involved in multiple signaling pathways, including glucose metabolism and insulin sensitivity; therefore, dysregulation of this enzyme is implicated in diseases such as diabetes. Insulin promotes the phosphorylation of GSK3 which, when activated, inhibits glycogen synthesis by downregulate the activity of glycogen synthase. In patients with T2D associated with obesity, inhibition of GSK3 diminishes hepatic glucose production. In this sense, pharmacological inhibition of GSK3 may improve insulin sensibility and glucose metabolism. Currently, potential GSK3 inhibitors have been tested *in vitro* and *in silico* (Teli and Gajjar, 2023; Wang et al., 2025). For instance, compounds isolated from *Hovenia dulcis* Thunb. Were tested *in silico* to elucidate the action mechanism of the plant as hypoglycemic. The results showed that compounds may activate AKT1, which consequently inhibit the action of GSK3, increasing glucose storage due to the promotion of glycogen synthesis (De Godoi et al., 2021).

#### 3.2.4 Glucose production inhibition

In the early postabsorptive state, euglycemia is maintained by the endogenous glucose production in the liver to meet the energy demand primarily of the brain and red blood cells. During this nutritional phase, glycogenolysis accounts for nearly 50% of hepatic glucose output; however, its contribution decreases as the fast is prolonged to around 5% after 42 h (Sargsyan and Herman, 2019).

Studies performed to determine the contribution of endogenous glucose production to the hyperglycemic state in T2D patients indicate that glycogenolysis is not appropriately suppressed during a hyperglycemic clamp, suggesting that not only gluconeogenesis contributes to the excessive rates of hepatic glucose output observed in T2D, but also glycogenolysis (Rizza, 2010).

##### 3.2.4.1 Gluconeogenic rate-limiting enzymes

Gluconeogenesis is the metabolic pathway for the *de novo* glucose synthesis from pyruvate, amino acids, lactate, and glycerol, which is controlled by hormones such as glucagon or insulin. The latter is responsible for suppressing glucose production. In individuals with T2D, insulin resistance modifies hepatic glucose production through the exacerbated elevation of gluconeogenic rates by 40%–200%. One of the ways to control gluconeogenesis is regulating the activity of the rate-limiting enzymes, such as glucose-6-phosphatase, fructose-1,6-bisphosphatase, phosphoenolpyruvate carboxykinase, and pyruvate kinase. The inhibition of the activity of these enzymes may diminish fasting hyperglycemia in diabetic individuals. In this context, experiments have been conducted to evaluate the potential of many molecules to inhibit the above-mentioned enzymes using *in vitro* and *in silico* approaches (Lewis et al., 2021; Barroso et al., 2024). Since the inhibition of the gluconeogenic rate-limiting enzymes has been an important target to evaluate natural products as hypoglycemic agents, the inhibitory effect of some natural products has been assessed on glucose-6-phosphatase and fructose-1,6-bisphosphatase enzymes. In this context, the leaf extract of *Ocimum gratissimum* L. diminished the activity of glucose-6-phosphatase at a concentration of 50 μg/mL (Awwad et al., 2021). Likewise, caffeic acid and kaempferol-3-*O* (2,6-di-O-trans-*p*-coumaryl)-B-D-glucopyranoside, isolated from *Eryngium cymosum* F.Delaroche, showed to inhibit glucose-6-phosphatase with an IC_50_ = 719.8 μg/mL and IC_50_ = 27.7 μg/mL, respectively. In the same study, kaempferol-3-*O* (2,6-di-*O*-trans-*p*-coumaryl)-B-D-glucopyranoside showed an inhibition on fructose-1,6-bisphosphatase with IC_50_ = 52.5 ug/mL (Romo-Perez et al., 2022). On the other hand, the extracts of *Eryngium longifolium* Cav. and *Alsophila firma* (Baker) D.S.Conant were tested against fructose-1,6-bisphosphatase, exhibiting an IC_50_ = 93 μg/mL and IC_50_ = 45 μg/m, respectively (Andrade-Cetto et al., 2021).

##### 3.2.4.2 Glycogen phosphorylase (GP)

One of the therapeutic targets to decrease the augmented rates of hepatic glucose production that has become relevant in recent years is the glycogen phosphorylase (GP), which is the first and most important regulatory enzyme in the glycogen breakdown or glycogenolysis. This enzyme catalyzes the irreversible cleavage of glucose units from glycogen yielding glucose-1-phosphate and is regulated covalently through hormonal control and allosterically by high glucose levels (Blanco and Blanco, 2017; Hayes, 2017). The methods that have been used to evaluate the inhibitory effect of promising molecules on this enzymatic target have been the measurement of its activity *in vitro* and *ex vivo*. The anthocyanin fraction of the pomegranate juice potently inhibited muscle and liver isoforms of GP. Subsequent *in silico* experiments in the same study revealed that pelargonidin-3-*O*-glucoside was the responsible of the inhibition by its structural binding mode at the GP inhibitor site (Drakou et al., 2020).

#### 3.2.5 Insulin resistance driving factors

Insulin resistance, defined as the inability of insulin to adequately exert its functions on target tissues such as liver, skeletal muscle, and adipose tissue, is the central etiological factor underlying the development of several diseases like type 2 diabetes, cardiovascular disease, cancer, polycystic ovarian disease, and nonalcoholic fatty liver disease (Biddinger and Kahn, 2006). Among other risk factors, obesity, impaired glucose tolerance, alcoholism, smoking, dyslipidemia, hyperuricemia, and hypertension are related to the apparition of this pathological condition (Ahmed et al., 2021).

It is well-documented that obesity plays a major role in the development of insulin resistance. The obesogenic environment, promoted by enlarged and dysfunctional adipocytes derived from chronic overnutrition, leads to a low-grade systemic inflammation consisting of elevated free fatty acids, pro-inflammatory cytokines, and reactive oxygen species that interrupt insulin signaling (Ye, 2013; Ahmed et al., 2021).

##### 3.2.5.1 11β-hydroxysteroid dehydrogenase type 1 (11β-HSD1)

It has been suggested that the pro-inflammatory environment may increase the expression of 11β-HSD1, an enzyme widely expressed in insulin target organs, whose function is to convert inactive cortisone into active cortisol. This glucocorticoid stimulates energy expenditure, i.e., exerts catabolic effects on metabolism, such as inducing hepatic gluconeogenesis, inhibiting glucose uptake, and increasing free fatty acid release. In addition, it is involved in the development of insulin resistance by decreasing insulin receptor affinity and inducing post-receptor defects (Janssen, 2022). Therefore, recognizing and characterizing inhibitors of this glucocorticoid reductase enzyme could have a positive impact on the treatment of diseases related to insulin resistance and metabolic syndrome (Studzińska et al., 2018; Qi et al., 2024; Zhong et al., 2024). To identify molecules with inhibitory capacity of 11β-HSD1, *in vitro* concentration-response inhibitory enzyme assays are performed to determine IC_50_ values. These assays are complemented by *in vivo* and *in silico* studies to assess the effect of these inhibitors on metabolic parameters, as well as to determine their binding affinity with the enzyme. Some tanshinone IIA and cryptotanshinone derivatives and the extract of the *Spondias pinnata* (L.f.) Kurz fruits exhibited a significant *in vitro* 11β-HSD1 inhibitory activity (Deng et al., 2022; Wahab et al., 2023).

##### 3.2.5.2 c-Jun N-terminal kinase 1 (JNK1)

This serine/threonine kinase is an isoform ubiquitously expressed belonging to the mitogen-activated protein kinase (MAPK) family, which is involved in stress responses. Its functions include promoting proliferation and cell death depending on the context and duration of its activation. Pro-inflammatory cytokines released in the obesogenic environment induce JNK1 activation, which phosphorylates insulin pathway mediators on Ser/Thr, resulting in a reduction in their insulin-signal promoted Tyr phosphorylation and thus generating insulin resistance (Yung and Giacca, 2020). Given its relevance in metabolic diseases related to obesity, the effect of molecules on the signaling pathways in which JNK1 participates has been characterized (Li et al., 2020; Tang et al., 2022). The research carried out for this purpose involves the virtual screening of potential inhibitors using artificial intelligence (AI) and machine learning models and then conducting concentration-response inhibitory enzyme assays to obtain IC_50_ values. In addition, the characterization of the inhibitors has been performed by molecular docking, as well as the determination of their effect on the protein expression of JNK1 by Western blot in cell lines. Through AI were identified lariciresinol, tricin, and 4′-demethylepipodophyllotoxin as the most promising candidates for JNK1 inhibition; however, only tricin exhibited acceptable *in vitro* inhibitory activity (Yang et al., 2022).

#### 3.2.6 Insulin sensitization

Insulin sensitivity as a concept refers to the ability of insulin target organs to respond to physiological levels of the hormone insulin (Dimitriadis et al., 2021). In metabolic diseases such as T2D, metabolic syndrome, and cardiovascular disease, decreased insulin sensitivity is perceived, which is known as insulin resistance. In order to counteract insulin resistance, two ways can be recognized: increasing insulin sensitivity by promoting enhanced insulin function in its target organs through modulation of its signaling pathway and mimicking the action of insulin by promoting the actions that this hormone performs through external agents (Mirabelli et al., 2020; Mastrototaro and Roden, 2021).

##### 3.2.6.1 Protein tyrosine phosphatase 1B (PTP-1B)

Dephosphorylation of tyrosine residues catalyzed by protein tyrosine phosphatase 1B (PTP-1B), a negative regulatory mechanism of insulin action in insulin-sensitive tissues, has been associated to insulin resistance as overexpression of PTP-1B protein has been reported in insulin-resistant states related to obesity (Rondinone et al., 2002; Panzhinskiy et al., 2013).

PTP-1B is a classical intracellular tyrosine phosphatase expressed ubiquitously that is involved in several cellular processes required for maintaining overall homeostasis and is therefore strictly controlled at the transcriptional, post-transcriptional, and post-translational levels (Bakke and Haj, 2015). Due to its well-established role as a metabolic regulator, PTP-1B is widely recognized as a good pharmacological target for type 2 diabetes (Sun et al., 2016). Particularly, liver-specific PTP-1B deletion in mice has shown to improve glucose homeostasis, lipid profile, insulin signaling, and insulin sensitivity in addition to inhibiting gluconeogenic enzymes and hepatic glucose production (Teimouri et al., 2022). The most common methodology used to evaluate the inhibitory effect of potential molecules has been *in vitro* concentration-response inhibition assays to determine the IC_50_. Recent studies have focused on identifying potential allosteric inhibitors of PTP-1B with improved specificity and activity. SarathKumar and Lakshmi (2019) evaluated the allosteric effectiveness of six natural compounds isolated from medicinal plants that showed PTP-1B inhibition. Furthermore, an ethanolic extract of *Ficus deltoidea* Jack, rich in phenolic compounds and triterpenes, significantly downregulated the expression of the hepatic gene PTP-1B (Abdel-Rahman et al., 2020).

##### 3.2.6.2 AMP-activated protein kinase (AMPK)

AMP-activated protein kinase (AMPK) is an energy status sensor with dozens of physiological targets, acting as a key regulator of both cellular and physiological energy balance. AMPK is activated upon detecting an increase in the cytosolic AMP:ATP ratio which simultaneously promotes catabolic processes of ATP production and represses anabolic energy-consuming processes (Steinberg and Carling, 2019). The AMPK signaling cascade involves the enzymatic regulation of carbohydrate, lipid, and protein metabolism by activating their oxidation and inhibiting their synthesis (Sharma et al., 2023).

Due to its crucial role in physiology and pathology, AMPK is a drug target for various diseases, including obesity, diabetes, and cancer. Many of the metabolic pathways regulated by AMPK were identified by incubating cells or injecting animals with the adenosine analog 5-aminoimidazole-4-carboxamide-1-β-D-ribofuranoside (AICAR) (Trefts and Shaw, 2021). Chronic AMPK activation with AICAR has been shown to improve treadmill running capacity and metabolic gene expression in young mice. (Wilcox et al., 2025). Another pharmacological tool for the activation of AMPK with insulin-sensitizing effect is the use of biguanides both in intact cells, *in vivo* and *ex vivo* (Towler and Hardie, 2007). The natural derivative mangiferin TPX showed to improve insulin resistance in human hepatocytes HepG2 and HL-7702 by interacting with the AMPK protein and triggering AKT phosphorylation, thereby activating the insulin signaling pathway (Fan et al., 2023). Likewise, AMPK activation mediated by the herbal alkaloid hernandezine was described (Bai et al., 2022).

#### 3.2.7 B-cell protection

Beta-cell dysfunction is considered the critical pathway responsible for the development of T2D from a prediabetic condition. When pancreatic β cells are no longer able to secrete insulin to compensate for insulin resistance, a marked increase in hyperglycemia occurs. Consequently, the search for molecules capable of promoting cell survival and protection to enhance insulin secretion is a novel approach that has emerged in recent years (Salunkhe et al., 2018). One of the main identified mechanisms that promote β-cell failure is dedifferentiation, a process in which β cells lose their identity and transform into nonfunctional endocrine progenitor-like cells. It has been reported that this phenomenon can be reversed as a therapeutic strategy to promote β-cell regeneration (Son and Accili, 2023).

##### 3.2.7.1 Dipeptidyl peptidase 4 (DPP-4)

DPP-4 is a serine protease that participates in the regulation of incretin action through catalytic degradation of glucagon-like peptide-1 (GLP-1) and glucose-dependent insulinotropic polypeptide (GIP). These hormones, in addition to exacerbating the glucose-stimulated insulin secretion response, promote β-cell function and survival; however, their half-life is short due to the action of the DPP-4 enzyme. Therefore, efforts have been made to characterize inhibitors that prolong the lifespan of incretins (Campbell et al., 2016; Naim, 2024). The methodology used in recent years to achieve this goal has been the development of concentration-response inhibitory enzyme assays and molecular docking. Natural products such as boschnaloside, the major iridoid glycoside of *Boschniakia rossica* (Cham. and Schltdl.) B.Fedtsch., have shown to improve β-cell function and reduce DPP-4 activity (Lin et al., 2019), while other compounds, like cannabinoids and squalene, exerted a potent inhibitory effect on the enzyme (Widyawati et al., 2023; Mkabayi et al., 2024). In addition, chlorogenic acid was found to have a great affinity for DPP-4 through molecular docking (Balogun et al., 2022). On the other hand, plant extracts also possess inhibitory capacity on DPP-4; for instance, *Hibiscus × rosa-sinensis* L., *Alnus nitida* (Spach) Endl., and *Dicoma anomala* Sond (Ansari et al., 2020; Matsabisa et al., 2020; Sajid et al., 2020). Moreover, *Corymbia citriodora* (Hook.) K.D.Hill and L.A.S.Johnson, which showed to improve glucose tolerance and plasma insulin in high-fat-fed rats, attenuated plasma DPP-4 activity and increased GLP-1 levels in circulation (Ansari et al., 2022).

#### 3.2.8 Lipid metabolism improvement

Dyslipidemia and hyperglycemia are strongly interconnected pathophysiological conditions that lead to atherosclerotic vascular disease, a common long-term complication of diabetes. The presence of large amounts of triglycerides, packaged into lipoproteins for transport through the bloodstream, as well as free fatty acids, may be not only the main causes of insulin resistance and cardiovascular disease, but may also be the consequence of diabetes (Parhofer, 2015; Kane et al., 2021). Hence, improving lipid metabolism is one of the main objectives of drug therapy to treat metabolic diseases, where organs such as the liver and adipose tissue are particularly relevant as therapeutic targets.

##### 3.2.8.1 Peroxisome proliferator-activated receptors (PPAR)

Peroxisome proliferator-activated receptors (PPAR-α, PPAR-β/δ, and PPAR-γ) are nuclear receptors that function as ligand-activated transcription factors (Wagner and Wagner, 2020). They are constitutively bound to response elements forming a heterodimer with retinoid X receptors (RXR) and regulate lipid metabolism and energy homeostasis by exerting direct control of metabolic gene expression (Wall et al., 2016).

The PPAR superfamily is considered an important therapeutic target for chronic diseases such as diabetes, cancer, and atherosclerosis (Mirza et al., 2019). For instance, PPARα increases fatty acid uptake, esterification, and cellular trafficking, and regulates lipoprotein metabolism genes; PPARδ stimulates lipid and glucose utilization by increasing mitochondrial function and fatty acid desaturation pathways, while PPARγ promotes fatty acid uptake, triglyceride formation and storage in lipid droplets, thereby increasing insulin sensitivity and glucose metabolism (Montaigne et al., 2021). Biomedical research into PPAR-targeted ligands is justified due to the importance of their biological functions, as PPAR agonists have been shown to improve insulin resistance, cardiometabolic health, and liver health (Cooreman et al., 2024). The triterpenoids alpha-amyrin and lupeol from *Hibiscus sabdariffa* L. activated PPARδ and PPARγ receptors, thereby increasing the expression of GLUT4 and improving insulin sensitization (Giacoman-Martínez et al., 2019). On the other hand, treatment with LDT409, a fatty acid-like compound derived from cashew nutshell liquid, was identified as a pan-PPAR partial agonist, which was effective in reversing high-calorie diet-induced obesity and associated metabolic abnormalities in mice (Sahin et al., 2024).

##### 3.2.8.2 Low-density lipoprotein receptor (LDLr)

The low-density lipoprotein receptor (LDLr) is involved in the regulation of cholesterol homeostasis, specifically participating in cholesterol production in response to variations in intracellular sterol concentrations and cellular cholesterol demand (Masana et al., 2013). Lipoprotein receptors are expressed in multiple vascular cell types and mediate the action of a wide range of ligands to accelerate or block atherogenesis. Research into therapeutic options related to LDLr primarily focuses on preventing or reducing atherosclerosis. In this context, *in vitro* culture models or murine models have allowed the study of vascular disorders (Mineo, 2020). For instance, the effect of six compounds from *Rydingia michauxii* (Briq.) Scheen and V.A.Albert on the LDLr was evaluated in the human hepatocarcinoma cell line Huh7, where a hypocholesterolemic effect was observed and a mechanism similar to statins was suggested (Sut et al., 2022).

#### 3.2.9 Sirtuin modulation

Sirtuins are a family of NAD + -dependent histone deacetylases that are involved in various cellular processes and are activated under conditions of energy imbalance. It is thought that modulation of these enzymes could imply a novel therapeutic approach for the treatment of diseases such as cancer and diabetes. Regarding glucose and lipid metabolism, it has been reported that SIRT1 regulates positively systemic insulin sensitivity, pancreatic insulin secretion, and activation of AMPK and PTP-1B. SIRT6, on the other hand, participates in the maintenance of glucose homeostasis by increasing GLUT2 expression in pancreatic β cells and enhancing insulin sensitivity in liver and muscle. Both isoforms transcriptionally repress lipogenesis. SIRT2 activates gluconeogenesis when nutrient shortages occur and inhibits lipid synthesis, while SIRT3 enhances cellular respiration and stimulates fatty acid oxidation (Wu et al., 2022). Research to discover sirtuin modulators has focused on molecular docking analysis and concentration-response activation enzyme assays. Molecules that have been documented as sirtuin activators have also been evaluated in *in vivo* models to characterize their impact on the expression of these enzymes and metabolic homeostasis. In this sense, silymarin, a flavonolignan compound, has shown to increase the enzymatic activity and expression of SIRT1, which was related to the amelioration of insulin resistance of high-fat-fed mice (Feng et al., 2021). On the other hand, six compounds isolated from some *Ficus* species (gossypetin, herbacetin, kaempferol, leucoperalgonidin, leucodelphinidin, and sorbifolin) were identified as potential ligands of SIRT6 by molecular docking (Singh et al., 2019).

## 4 Discussion

The objective of this review was to identify the main pharmacological targets employed to assess the hypoglycemic effects of natural products, with particular emphasis on alternatives to the use of animal models. According to the results, nine major pharmacological mechanisms have been identified as the primary targets of natural products evaluated over the last 5 years using *in vivo*, *in vitro*, *in silico*, and *ex vivo* approaches. Although there is a wide range of methods to evaluate the effect of natural products for the treatment of diabetes, animal models remain important for drug development; however, the use of *in vitro* and *in silico* methodologies is increasing. In this sense, complete replacement of animal models is not currently feasible, although certain *in vitro* and *in silico* approaches can partially substitute for animal experiments in specific mechanisms. For instance, while *in vitro* experiments, such as glucose uptake assays or enzyme assays, are useful for detecting the specific effect on a particular target, they do not consider the physiological context provided by the *in vivo* approach.

Previous reviews have emphasized the specific mechanisms that different natural products exert to regulate metabolism, focusing on the nature of the compounds rather than on the mechanisms of action (Sharma et al., 2018; Xu et al., 2018; Aguerd et al., 2025). Therefore, this review focuses on grouping the most studied mechanisms of action in the area of natural products to combat diabetes with the aim of serving as a foundation for identifying alternatives to animal use in hypoglycemic natural-product research. We highlight experimental approaches, such as enzymatic assays, which can be employed to evaluate the effects of natural products on mechanisms including hepatic glucose production and carbohydrate digestion. For mechanisms in which replacement of animal models is not possible, reducing the number of animals used and refining experimental techniques remain essential, along with the responsible and ethical use of animals in research.

Natural products have demonstrated multiple mechanisms of action against T2D. These include the inhibition of hepatic glucose production through the reduction of enzymatic activity or downregulation of enzyme expression. Additional mechanisms by which natural products may improve the diabetic condition involve decreasing intestinal glucose absorption and enhancing peripheral glucose uptake. Although specific natural products and their mechanisms of action can be mentioned, the primary objective of this review was to propose alternatives to the use of animal models, using selected natural products previously evaluated as illustrative examples.

The search for drugs based on natural products from an ethnopharmacological perspective stems from the traditional use of biologically active products, which allows for the study of their pharmacological effects. In the case of metabolic diseases such as T2D, medicinal preparations are often consumed orally, the most widely route of administration due to its simplicity and convenience. The active compounds present in these preparations exhibit systemic effects whose synergy can be crucial to achieving the desired therapeutical effect (Yebouk et al., 2020; Chakale et al., 2021; Fakchich and Elachouri, 2021; Nguyen et al., 2022; Iwaka et al., 2023).

Owing to the route of administration used by patients with T2D, the assessment of the potential therapeutical effect of natural products must consider the pharmacokinetic processes involved in the oral route. In this scenario, animal models play a fundamental role in the drug discovery and development. Historically, animals have been used as experimental subjects in research on human diseases due to the ethical and safety concerns involved in conducting research directly in humans. Thanks to the use of animal models, it has been possible to understand the pathological processes of diseases to develop diagnostic strategies and facilitate the design of more effective and safer drugs and therapies (El Sayed Abd El-Aal, 2014; Verdier et al., 2015). In this sense, the animal model of choice to evaluate the effect of natural products on metabolic parameters related to T2D is that induced by a high-fat diet and streptozotocin. This model is useful because it resembles the natural history of the disease and presents the most important characteristics of T2D, such as hyperglycemia, insulin resistance, and pancreatic β-cell dysfunction. Furthermore, it has been used to evaluate the impact of molecules or extracts on the overall phenotype of insulin resistance or impaired insulin secretion. However, the main limitation is that specific action mechanism cannot be identified from the natural product of interest. Therefore, several *in vitro*, *in silico*, and *ex vivo* methods have been implemented that limit the system to a particular protein, cell, or tissue.

Scientific advances have allowed the development of alternatives to the use of animals, which could reduce their use in pharmaceutical research. However, it is not yet possible to eliminate them, so scientists have an obligation to ensure their wellbeing and minimize any discomfort or pain they may experience. In this context, it is necessary to adopt approaches that prioritize both the animal welfare and the robustness of studies, which involves optimizing experimental design to ensure reproducibility and improve results. To address this issue, tools such as the ARRIVE guidelines have been developed, which are designed to improved transparency, quality, and reproducibility in animal research (Percie du Sert et al., 2020).

It is also important refine maintenance, handling, and sampling techniques to minimize discomfort, as well as maintain an objective and ongoing pain assessment program that includes tools such as grimace scales, physiological parameter measurement, and staff skills development to identify any behavioral abnormalities (Broomé et al., 2023). When performing any surgical procedure, proper pain management is essential, as it directly impacts animal welfare and contributes to the validity of the research. In this regard, the most current and recommended approach is multimodal pain management, which suggests the use of drugs with different therapeutic targets. This allows for reduced administered doses and enhanced overall analgesic effect, thereby reducing side effects without compromising data accuracy (Gomez de Segura et al., 2025). Finally, it is essential to clearly and definitively establish humane endpoints in pharmacological research that aim to avoid unnecessary animal suffering, thereby reducing the use of animal lives and complying with international regulations (Najafi et al., 2021; Williams and Baneux, 2022).

Some of the challenges of using animals for drug development include high costs and long development times. To overcome this, *in silico* or computational models can be applied to enable rapid and cost-effective evaluation of large numbers of drug candidate molecules, significantly reducing the number of animals required to perform the same task (Piñero et al., 2018; El-Tanani et al., 2025). These models also can emulate biological systems such as cells and organs, or even simulate clinical trials, thus facilitating the understanding of a drug’s pharmacokinetic and pharmacodynamic processes. Furthermore, *in silico* models can analyze a large amount of information, facilitating to elucidate and understand the pathophysiological mechanisms characteristic of diseases and contributing to the discovery of new therapeutic targets (Shekhar, 2008). Virtual experiments that recreate biological and chemical reactions can also be performed to analyze the affinity of candidate molecules with specific therapeutic targets (El-Tanani et al., 2025). However, the main limitations of *in silico* methods are that they offer an incomplete biological representation, limited modeling of disease heterogeneity, limited predictive power for long-term outcomes, and oversimplification of pharmacokinetics and pharmacodynamics. Because T2D is a highly heterogeneous, multifactorial, and multisystemic chronic disease, *in silico* models do not have the ability to fully capture organ-organ interaction, hormonal feedback loops, or long-term adaptations, simplifying interactions and limiting the reliability of models to predict dose-response relationships or adverse effects.

Cell and tissue cultures used in pharmacological research also offer alternatives to animal testing (BéruBé et al., 2010). One of their key advantages is the use of human tissue, which enables results with high extrapolative value. Additionally, they support the analysis of uptake, transport, and metabolism of candidate molecules within a controlled environment, allowing for the elucidation of mechanisms of action through rapid and cost-effective evaluation—often more efficient than traditional animal testing (LeDOUX, 2005; Peppas and Carr, 2009; Ehrhardt et al., 2017). Their specific uses in drug development focus on toxicology and metabolic activity (Spanu et al., 2022). However, although they are a widely used method, they have several limitations, such as their low complexity compared to a living organism, which limits the multi-organ interactions that occur within it (Ehrhardt et al., 2017). Moreover, traditional 2D cell culture models do not represent an organ’s microenvironment, which can influence the characteristics that give tissue functionality and therefore the outcomes (Buchheiser et al., 2012; Deng et al., 2023).

The pathophysiological processes that occur in T2D are complex and difficult to replicate. However, for several years, techniques have been available that utilize 3D tissue engineering and microfluidics technology, which in combination can develop organ-on-chips. These techniques, although little applied, have emerged as promising tools for emulating these and many other pathological processes (Rogal et al., 2019; Deng et al., 2023). Among their most notable features, organs-on-chips exhibit high levels of cellular differentiation and can replicate the biochemical and cellular microenvironment of human organs. They feature multiple cell types, allowing them to recreate the functional organization of specific organs (Lauffer, 2021). They also incorporate a fluid flow that mimics blood circulation and thus the nutrient delivery that occurs in real organs (Pimenta et al., 2022; Keuper-Navis et al., 2023). They also can emulate mechanical forces that are very important for the functionality of specific tissues such as the lungs (Lauffer, 2021). It is also possible to interconnect different organ chips into a single system, permitting real-time measurements and expanding the possibilities for analyzing the data obtained (Rogal et al., 2019). Despite their advantages, organ-on-a-chip models have important limitations for drug discovery in diabetes research. T2D is a chronic, multisystem disease involving coordinated dysfunction of the pancreas, liver, muscle, adipose tissue, gut, and immune system, whereas most organ-on-chip platforms represent only single organs or limited inter-organ interactions. These systems are typically maintained for short periods, restricting their ability to model long-term disease progression. In addition, endocrine, neural, and immune components that critically regulate glucose homeostasis are often simplified or absent. Technical challenges such as drug absorption by chip materials, limited throughput, high costs, and lack of standardization further constrain reproducibility and scalability. Consequently, although organ-on-chip systems improve human relevance compared with conventional *in vitro* models, they currently remain complementary rather than standalone alternatives for diabetes research and drug development.

Another technology developed alongside organs-on-chips is organoids, whose goal is to mimic the macroscopic and microscopic anatomy, as well as the functionality, of real tissues. Organoids are three-dimensional multicellular structures cultured *in vitro* from embryonic stem cells, induced pluripotent stem cells, or adult stem cells that differentiate into the different cell types present in an organ (Qu et al., 2023). The ability of organoids to mimic a real organ is especially important in the development of new drugs, as it facilitates and improves the testing of therapies and drugs. They are also particularly useful for high-fidelity evaluations of efficacy and toxicity, as well as adverse reactions and metabolism of these drugs (Heinzelmann et al., 2024; Xiang et al., 2024; Zhang Y. et al., 2025). Organoids also have the potential to be used for personalized medicine research, as they can be developed from patient tissue, targeting and personalizing therapies (Dumont et al., 2019; Heinzelmann et al., 2024). As well as organ-on-chips, organoid technologies have several limitations as typically represent isolated tissues and lack inter-organ communication essential for glucose homeostasis. Many pancreatic, hepatic, or intestinal organoids exhibit immature or fetal-like phenotypes, limiting their ability to model adult diabetic states, β-cell dysfunction, and insulin resistance accurately. Organoids also lack vascularization, immune components, and neural inputs, restricting studies of inflammation, endocrine regulation, and metabolic signaling. Moreover, long-term culture stability, variability between batches, and limited standardization further reduce reproducibility and scalability.

The most recent advancement in pharmacological research is the use of AI, which is characterized by machine learning, natural language processing, decision-making and reasoning, and process automation (Xu et al., 2021; Singh and Kaur, 2025). This enables the streamlining and optimization of drug development phases, particularly through the analysis of large-scale genomic, proteomic, metabolomic, and other datasets to identify novel therapeutic targets. It also facilitates the extraction of relevant information from medical records and the integration of diverse data sources to validate, prioritize, and, when appropriate, confirm the relevance of these targets. In drug discovery, it simulates molecular interactions with therapeutic targets, designs new compounds, and generates molecular structures with desired properties. Additionally, it predicts pharmacokinetic profiles and supports the design of synthetic routes for new molecules. During preclinical studies, it enhances experimental design, identifies predictive biomarkers, and estimates effective and safe dosage ranges. In clinical trials, it forecasts the physical, chemical, and biological properties of candidate molecules, assesses personalized efficacy, anticipates potential adverse effects, and contributes to the design of more efficient trials. Moreover, it strengthens pharmacovigilance by enabling rapid detection and reporting of side effects (Pettersen et al., 2021; Tiwari et al., 2023; Zhang K. et al., 2025). However, AI models are highly dependent on the quality, size, and representativeness of training datasets, which are often biased, incomplete, or derived from specific populations, limiting generalizability. AI systems struggle to capture the complex, dynamic, and long-term pathophysiology of diabetes, including disease progression, β-cell failure, and lifestyle-driven variability. Integration of heterogeneous data sources (clinical, omics, behavioral, and environmental) remains challenging, and real-world performance often declines due to missing data or inconsistent monitoring.

Enzyme assays represent another alternative for drug discovery in diabetes. These tools allow for the study of a specific target under highly controlled conditions and are ideal for mechanistic studies. Furthermore, their application enables the screening of a large number of molecules, requiring only a small sample. Moreover, they are cheaper than other models such as organ-on-chips and offer high reproducibility. However, due to the lack of physiological context, it is difficult to translate the results obtained to whole organisms, and the pharmacokinetic properties of the tested products are not considered. Despite this, they remain the most widely used methodologies when evaluating natural products because they are more accessible than other methods.

In December 2022, the FDA Modernization Act 2.0 was enacted in the United States, allowing for the approval of drugs without animal testing, thus refuting the legislation in place since 1938 that sought to use animals in the development of new drugs to guarantee the highest standards of quality and biosafety. However, although new approach methodologies allow for better capture of human-relevant biology and may accelerate the movement toward phase 1 clinical trials of promising natural products, the use of animals in drug testing is far from extinct, as in many cases, properly validated alternative methods still do not exist. Furthermore, drugs approved under this scheme must employ development methodologies that guarantee their efficacy and safety (Deng et al., 2023; Han, 2023). The goal is to reduce the number of animals used in drug development and promote the advancement of alternative methods that are ethically and economically less costly, while also reducing the development time for new therapies.

Finally, it must be mentioned that the best way to study diabetes is with patients; however, there are important bioethical limitations that prevent or limit this practice (Rogal et al., 2019). For this reason, the most recommended studies, due to their accessibility and low cost, are *in vitro* assays that include the evaluation of enzymatic inhibition of the targets proposed in this systematic review. In this context, according to the literature search, carbohydrate breakdown inhibition is the most assessed mechanism for determining the potential therapeutic effect of molecules from natural sources; however, despite being the most explored mechanism, it does not actually correct any pathophysiological abnormality of the disease, since the expression or activity of the enzymes that delay carbohydrate absorption are not altered in patients with T2D. Therefore, subsequent studies involving the search for new molecules with potential therapeutic effects to treat T2D should focus on limiting the activity of enzymes that directly participate in the generation of hyperglycemia, dyslipidemia, insulin resistance, etc., or on enhancing the activity of enzymes that improve insulin function or that protect pancreatic β cells. There is an area of opportunity in little-explored mechanisms that can be exploited in the search for new drugs to delay or reduce the disease.

## 5 Conclusion

Currently, the development of new drugs based on natural products for the treatment of T2D relies on practical and accessible resources that can help reduce the use of experimental animals. However, predicting a drug’s success in clinical practice remains difficult due to the multiple interactions that occur within an organism, even when its potential has been demonstrated, such as its molecular affinity, evidence of its *in vitro* effect, and biological plausibility, among other factors. In this sense, at least for now, these techniques can optimize the use of animal models in preclinical research, preselecting the most promising molecules, improving their *in vivo* safety, and reducing the number of trials required to demonstrate a significant effect. According to this systematic review, the efforts of the scientific community should focus on studying little-explored mechanisms with significantly greater impact in the search for new molecules with potential therapeutic effects; for instance, promotion of glucose storage, inhibition of glucose production, insulin sensitization, and functional improvement of the β cell.
