# Supplementary material for: Alternative methods for pharmacological research on the action mechanisms of natural products used in the treatment of type 2 diabetes: a systematic review
Source: Front Pharmacol. 2026 Feb 10;17:1729030. doi: 10.3389/fphar.2026.1729030 (PMC12929498; doi:10.3389/fphar.2026.1729030)
Supplement: Supplementary file 1 [file Supplementaryfile1.docx]

Supplementary Material

**
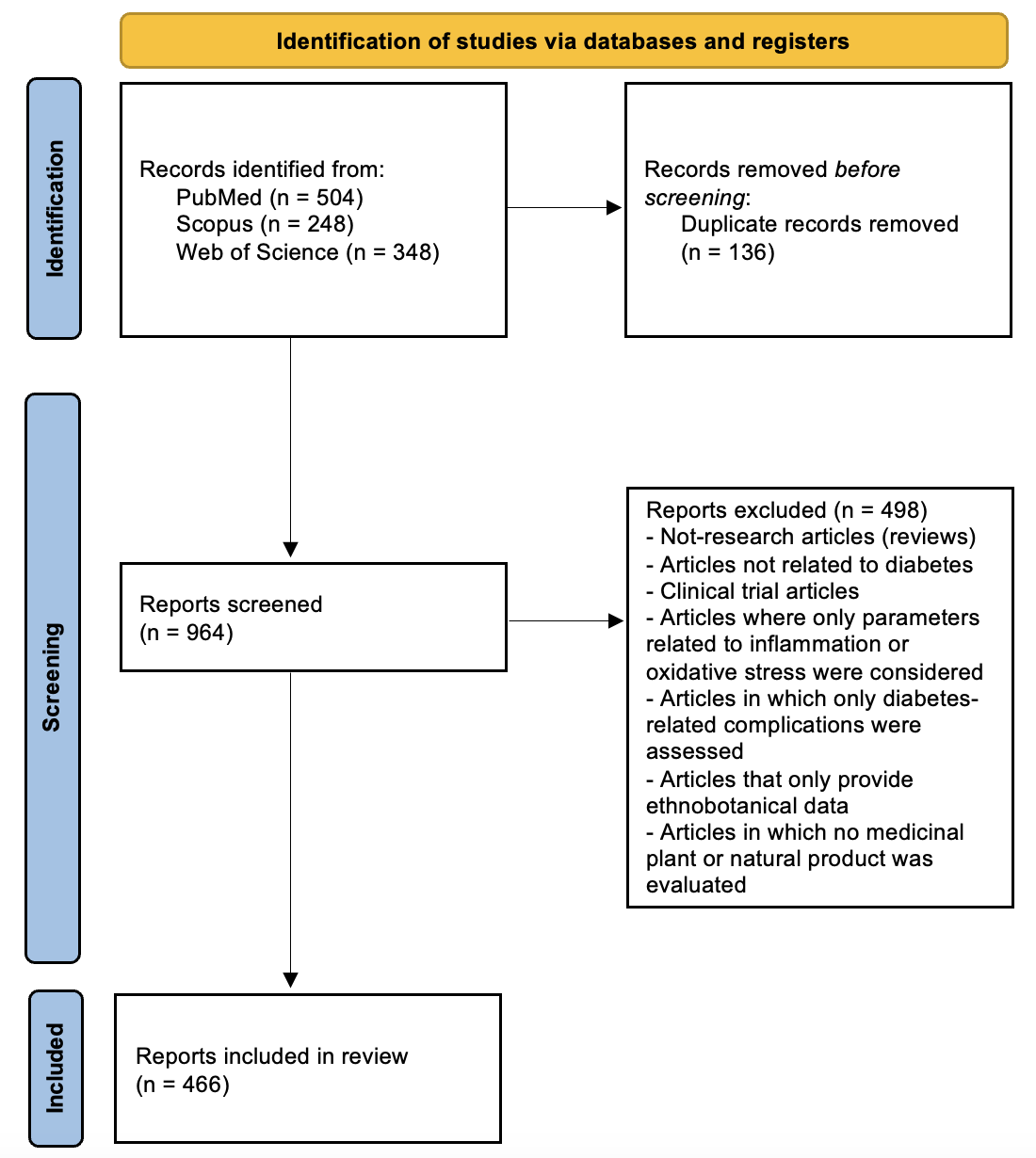
**

**Supplementary Figure 1.** PRISMA flowchart.

**Supplementary Table 1.** Resulting references grouped by mechanism.

| **Mechanism** | **References** |
| --- | --- |
| Carbohydrate breakdown inhibition | Abu-Odeh, A., Shehadeh, M., Suaifan, G. A. R. Y., Karameh, N., Abdel Rahman, D., and Kandil, Y. (2022). In Vitro and In Vivo Antidiabetic Activity, Phenolic Content and Microscopical Characterization of Terfezia claveryi. *Molecules* 27, 4843. doi: 10.3390/molecules27154843  Abudurexiti, A., Zhang, R., Zhong, Y., Tan, H., Yan, J., Bake, S., et al. (2023). Identification of α-glucosidase inhibitors from Mulberry using UF-UPLC-QTOF-MS/MS and molecular docking. *J. Funct. Foods* 101, 105362. doi: 10.1016/j.jff.2022.105362  Ajiboye, B. O., Ojo, O. A., Oyinloye, B. E., Akuboh, O., Okesola, M. A., Idowu, O., et al. (2020). In vitro antioxidant and inhibitory activities of polyphenolic-rich extracts of Syzygium cumini (Linn) Skeels leaf on two important enzymes relevant to type II diabetes mellitus. *Pak. J. Pharm. Sci.* 33, 523–529. doi: https://pubmed.ncbi.nlm.nih.gov/32276893/  Al-Araby, S. Q., Rahman, M. A., Chowdhury, M. A. H., Das, R. R., Chowdhury, T. A., Hasan, C. M. M., et al. (2020). Padina tenuis (marine alga) attenuates oxidative stress and streptozotocin-induced type 2 diabetic indices in Wistar albino rats. *South African J. Bot.* 128, 87–100. doi: 10.1016/j.sajb.2019.09.007  AL-Bukhaiti, W. Q., Al-Dalali, S., Noman, A., Qiu, S., Abed, S. M., and Qiu, S.-X. (2022). Response Surface Modeling and Optimization of Enzymolysis Parameters for the In Vitro Antidiabetic Activities of Peanut Protein Hydrolysates Prepared Using Two Proteases. *Foods* 11, 3303. doi: 10.3390/foods11203303  Aladejana, A. E., Bradley, G., and Afolayan, A. J. (2021). In vitro evaluation of the anti-diabetic potential of Helichrysum petiolare Hilliard &amp; B.L. Burtt using HepG2 (C3A) and L6 cell lines. *F1000Research* 9, 1240. doi: 10.12688/f1000research.26855.2  Alongi, M., Frías Celayeta, J. M., Vriz, R., Kinsella, G. K., Rulikowska, A., and Anese, M. (2021). In vitro digestion nullified the differences triggered by roasting in phenolic composition and α-glucosidase inhibitory capacity of coffee. *Food Chem.* 342, 128289. doi: 10.1016/j.foodchem.2020.128289  Alsawalha, M., Al-Subaei, A., Al-Jindan, R., Bolla, S., Sen, D., Balakrishna, J., et al. (2019). Anti-diabetic activities of Dactylorhiza hatagirea leaf extract in 3T3-L1 cell line model. *Pharmacogn. Mag.* 15, 212. doi: 10.4103/pm.pm_8_19  Alu’datt, M. H., Al‐u’datt, D. G. F., Alhamad, M. N., Tranchant, C. C., Rababah, T., Gammoh, S., et al. (2021). Characterization and biological properties of peptides isolated from dried fermented cow milk products by RP‐HPLC: Amino acid composition, antioxidant, antihypertensive, and antidiabetic properties. *J. Food Sci.* 86, 3046–3060. doi: 10.1111/1750-3841.15794  Álvarez-Almazán, S., Solís-Domínguez, L. C., Duperou-Luna, P., Fuerte-Gómez, T., González-Andrade, M., Aranda-Barradas, M. E., et al. (2023). Anti-Diabetic Activity of Glycyrrhetinic Acid Derivatives FC-114 and FC-122: Scale-Up, In Silico, In Vitro, and In Vivo Studies. *Int. J. Mol. Sci.* 24, 12812. doi: 10.3390/ijms241612812  Amankwaah, F., Addotey, J. N., Orman, E., Adosraku, R., and Amponsah, I. K. (2023). A comparative study of Ghanaian propolis extracts: Chemometric analysis of the chromatographic profile, antioxidant, and hypoglycemic potential and identification of active constituents. *Sci. African* 22, e01956. doi: 10.1016/j.sciaf.2023.e01956  Amrita, C., Kaur, I., Sharma, A. D., Sarkar, A., and Panja, A. S. (2025). In vitro α-amylase and α-glucosidase inhibitors study of Cymbopogon martinii essential oil and molecular docking study of predominant components, PASS prediction and ADME/Tox studies. *Chem. Pap.* 79, 365–394. doi: 10.1007/s11696-024-03786-8  Andrade-Cetto, A., Espinoza-Hernández, F., and Mata-Torres, G. (2021). Hypoglycemic Effect of Calea urticifolia (Mill.) DC. *Evidence-Based Complement. Altern. Med.* 2021, 1–10. doi: 10.1155/2021/6625009  Andrade-Cetto, A., Cruz, E. C., Cabello-Hernández, C. A., and Cárdenas-Vázquez, R. (2019). Hypoglycemic Activity of Medicinal Plants Used among the Cakchiquels in Guatemala for the Treatment of Type 2 Diabetes. *Evidence-Based Complement. Altern. Med.* 2019, 1–7. doi: 10.1155/2019/2168603  Ansari, P., Hannan, J. M. A., Choudhury, S. T., Islam, S. S., Talukder, A., Seidel, V., et al. (2022). Antidiabetic Actions of Ethanol Extract of Camellia sinensis Leaf Ameliorates Insulin Secretion, Inhibits the DPP-IV Enzyme, Improves Glucose Tolerance, and Increases Active GLP-1 (7–36) Levels in High-Fat-Diet-Fed Rats. *Medicines* 9, 56. doi: 10.3390/medicines9110056  Ansari, P., Flatt, P. R., Harriott, P., Hannan, J. M. A., and Abdel-Wahab, Y. H. A. (2021). Identification of Multiple Pancreatic and Extra-Pancreatic Pathways Underlying the Glucose-Lowering Actions of Acacia arabica Bark in Type-2 Diabetes and Isolation of Active Phytoconstituents. *Plants* 10, 1190. doi: 10.3390/plants10061190  Añibarro-Ortega, M., López, V., Núñez, S., Petrović, J., Mandim, F., Barros, L., et al. (2024). Phenolic composition and in vitro bioactive and enzyme inhibitory properties of bell pepper (Capsicum annuum L.) plant extracts. *Ind. Crops Prod.* 214, 118546. doi: 10.1016/j.indcrop.2024.118546  Asmara, A. P., Prasansuklab, A., Tencomnao, T., and Ung, A. T. (2023). Identification of Phytochemicals in Bioactive Extracts of Acacia saligna Growing in Australia. *Molecules* 28, 1028. doi: 10.3390/molecules28031028  Atchan Nwakiban, A. P., Sokeng, A. J., Dell’Agli, M., Bossi, L., Beretta, G., Gelmini, F., et al. (2019). Hydroethanolic plant extracts from Cameroon positively modulate enzymes relevant to carbohydrate/lipid digestion and cardio-metabolic diseases. *Food Funct.* 10, 6533–6542. doi: 10.1039/C9FO01664C  Awote, O. K., Kanmodi, R. I., Ebube, S. C., and Abdulganniyyu, Z. F. (2024). Nutritional Profile, GC-MS Analysis and In-silico Anti-diabetic Phytocompounds Candidature of Jatropha gossypifolia Leaf Extracts. *Curr. Drug Discov. Technol.* 21. doi: 10.2174/0115701638267143230925172207  Aylanc, V., Eskin, B., Zengin, G., Dursun, M., and Cakmak, Y. S. (2020). In vitro studies on different extracts of fenugreek ( Trigonella spruneriana BOISS.): Phytochemical profile, antioxidant activity, and enzyme inhibition potential. *J. Food Biochem.* 44. doi: 10.1111/jfbc.13463  B, A. S., V, S., and R, K. (2021). IN-VITRO INHIBITION OF TYPE 2 DIABETES KEY ENZYMES; EFFECT OF EXTRACTS AND SOLVENT-SOLVENT FRACTIONS OF DANIELLIA OLIVERI (ROLFE) HUTCH. &amp; DALZIEL. *Bull. Pharm. Sci. Assiut* 44, 415–426. doi: 10.21608/bfsa.2021.207160  Balogun, F. O., Singh, K., Rampadarath, A., Akoonjee, A., Naidoo, K., and Sabiu, S. (2023). Cheminformatics identification of modulators of key carbohydrate-metabolizing enzymes from C. cujete for type-2 diabetes mellitus intervention. *J. Diabetes Metab. Disord.* 22, 1299–1317. doi: 10.1007/s40200-023-01249-7  Balu, P., Sonia Jas, J., and Govindaraj, M. (2019). Design and evaluation of chalconeimine derivatives as α-amylase inhibitors. *Bioinformation* 15, 523–529. doi: 10.6026/97320630015523  Bendjedou, H., Benamar, H., Bennaceur, M., Rodrigues, M. J., Pereira, C. G., Trentin, R., et al. (2023). New Insights into the Phytochemical Profile and Biological Properties of Lycium intricatum Bois. (Solanaceae). *Plants* 12, 996. doi: 10.3390/plants12050996  Bian, G., Hu, Y., Yan, K., Xin-jie Cheng, and Li, D. (2022). Characterization of constituents by UPLC-MS and the influence of extraction methods of the seeds of Vernonia anthelmintica willd.: extraction, characterization, antioxidant and enzyme modulatory activities. *Heliyon* 8, e10332. doi: 10.1016/j.heliyon.2022.e10332  Bonesi, M., Saab, A. M., Tenuta, M. C., Leporini, M., Saab, M. J., Loizzo, M. R., et al. (2020). Screening of traditional Lebanese medicinal plants as antioxidants and inhibitors of key enzymes linked to type 2 diabetes. *Plant Biosyst. - An Int. J. Deal. with all Asp. Plant Biol.* 154, 656–662. doi: 10.1080/11263504.2019.1674400  Boutahiri, S., Bouhrim, M., Abidi, C., Mechchate, H., Alqahtani, A. S., Noman, O. M., et al. (2021). Antihyperglycemic Effect of Lavandula pedunculata: In Vivo, In Vitro and Ex Vivo Approaches. *Pharmaceutics* 13, 2019. doi: 10.3390/pharmaceutics13122019  Caldeira, A. S. P., Mbiakop, U. C., Pádua, R. M., van de Venter, M., Matsabisa, M. G., Campana, P. R. V., et al. (2021). Bioguided chemical characterization of pequi (Caryocar brasiliense) fruit peels towards an anti-diabetic activity. *Food Chem.* 345, 128734. doi: 10.1016/j.foodchem.2020.128734  Capetti, F., Cagliero, C., Marengo, A., Bicchi, C., Rubiolo, P., and Sgorbini, B. (2020). Bio-Guided Fractionation Driven by In Vitro α-Amylase Inhibition Assays of Essential Oils Bearing Specialized Metabolites with Potential Hypoglycemic Activity. *Plants* 9, 1242. doi: 10.3390/plants9091242  Chanu, K. D., Sharma, N., Kshetrimayum, V., Chaudhary, S. K., Ghosh, S., Haldar, P. K., et al. (2023). Ageratina adenophora (Spreng.) King &amp; H. Rob. Standardized leaf extract as an antidiabetic agent for type 2 diabetes: An in vitro and in vivo evaluation. *Front. Pharmacol.* 14. doi: 10.3389/fphar.2023.1178904  Chelleng, N., Begum, T., Dutta, P. P., Chetia, P., Sen, S., Dey, B. K., et al. (2024). Antidiabetic potential of Amomum dealbatum Roxb. flower and isolation of three bioactive compounds with molecular docking and in vivo study. *Nat. Prod. Res.* 38, 3427–3432. doi: 10.1080/14786419.2023.2245115  Chen, X., Chen, C., and Fu, X. (2022). Hypoglycemic activity in vitro and vivo of a water-soluble polysaccharide from Astragalus membranaceus. *Food Funct.* 13, 11210–11222. doi: 10.1039/D2FO02298B  Chen, Q., Toy, J. Y. H., Seta, C., Yeo, T. C., and Huang, D. (2021). Inhibition Effect of Extract of Psychotria viridiflora Stem on α-Amylase and α-Glucosidase and Its Application in Lowering the Digestibility of Noodles. *Front. Nutr.* 8. doi: 10.3389/fnut.2021.701114  Chen, J., Wu, S., Zhang, Q., Yin, Z., and Zhang, L. (2020). α-Glucosidase inhibitory effect of anthocyanins from Cinnamomum camphora fruit: Inhibition kinetics and mechanistic insights through in vitro and in silico studies. *Int. J. Biol. Macromol.* 143, 696–703. doi: 10.1016/j.ijbiomac.2019.09.091  Chike-Ekwughe, A., Adegboyega, A. E., Johnson, T. O., Adebayo, A. H., and Ogunlana, O. O. (2023). In vitro and in-silico inhibitory validation of Tapinanthus cordifolius leaf extract on alpha-amylase in the management of type 2 diabetes. *Informatics Med. Unlocked* 36, 101148. doi: 10.1016/j.imu.2022.101148  Chike-Ekwughe, A., Adegboyega, A. E., Johnson, T. O., Adebayo, A. H., and Ogunlana, O. O. (2024). In vitro and in silico inhibitory validation of Tapinanthus cordifolius leaf extract on alpha-glucosidase in the management of type 2 diabetes. *J. Biomol. Struct. Dyn.* 42, 2512–2524. doi: 10.1080/07391102.2023.2212791  Chukiatsiri, S., Wongsrangsap, N., Ratanabunyong, S., and Choowongkomon, K. (2022). In Vitro Evaluation of Antidiabetic Potential of Cleistocalyx nervosum var. paniala Fruit Extract. *Plants* 12, 112. doi: 10.3390/plants12010112  Coutinho, M. R., da Silva, A. W., Ferreira, M. K. A., de Lima Rebouças, E., Mendes, F. R. S., Teixeira, E. H., et al. (2022). Hypoglycemic effect on adult zebrafish ( <scp> *Danio rerio* </scp> ) of the 3β‐6β‐16β‐trihydroxylup‐20(29)‐ene triterpene isolated from *Combretum leprosum* leaves in vivo and in silico approach. *Fundam. Clin. Pharmacol.* 36, 818–826. doi: 10.1111/fcp.12776  Cvetkova, M., Bandere, D., Lauberte, L., Niedra, S., and Teterovska, R. (2024). Polyphenol Content, Antiradical Properties, and α-Amylase Inhibition Activity of Vaccinium myrtillus L. (Bilberry) and Vaccinium vitis-idaea L. (Lingonberry) Leaf and Aerial Parts Extracts. *Appl. Sci.* 14, 5237. doi: 10.3390/app14125237  D, R. F. ; P. W. E. ; S. W. (2021). Virtual Assessment of Imperata Cylindrica Roots Bioactive Compounds as a Potential Inhibitor for Alpha-Glucosidase: the Study of Tengger Tribe’s Medicinal Plant. *Trop. J. Nat. Prod. Res.* 5, 1240–1245. doi: 10.26538/tjnpr/v5i7.13  Dall’Acqua, S., Ak, G., Sut, S., Zengin, G., Yıldıztugay, E., Fawzi Mahomoodally, M., et al. (2020). Comprehensive bioactivity and chemical characterization of the endemic plant Scorzonera hieraciifolia Hayek extracts: A promising source of bioactive compounds. *Food Res. Int.* 137, 109371. doi: 10.1016/j.foodres.2020.109371  Daou, M., Elnaker, N. A., Ochsenkühn, M. A., Amin, S. A., Yousef, A. F., and Yousef, L. F. (2022). In vitro α-glucosidase inhibitory activity of Tamarix nilotica shoot extracts and fractions. *PLoS One* 17, e0264969. doi: 10.1371/journal.pone.0264969  Devaraj, A., and Mahalingam, G. (2024). Green synthesis of Au Nps using Hemidesmus indicus root extract (Hire) and investigating its potential biomedical efficacies. *Chem. Pap.* 78, 2895–2914. doi: 10.1007/s11696-023-03280-7  Djeujo, F. M., Ragazzi, E., Urettini, M., Sauro, B., Cichero, E., Tonelli, M., et al. (2022). Magnolol and Luteolin Inhibition of α-Glucosidase Activity: Kinetics and Type of Interaction Detected by In Vitro and In Silico Studies. *Pharmaceuticals* 15, 205. doi: 10.3390/ph15020205  Dong, Q., Hu, N., Yue, H., Wang, H., and Wei, Y. (2023). Rapid screening of α ‐glucosidase inhibitors in Hypericum perforatum L. using bio‐affinity chromatography coupled with UPLC/MS. *Biomed. Chromatogr.* 37. doi: 10.1002/bmc.5536  Dong, Y., Yu, N., Li, X., Zhang, B., Xing, Y., Zhuang, C., et al. (2020). Dietary 5,6,7-Trihydroxy-flavonoid Aglycones and 1-Deoxynojirimycin Synergistically Inhibit the Recombinant Maltase–Glucoamylase Subunit of α-Glucosidase and Lower Postprandial Blood Glucose. *J. Agric. Food Chem.* 68, 8774–8787. doi: 10.1021/acs.jafc.0c01668  Đorđevski, N., Uba, A. I., Zengin, G., Božunović, J., Gašić, U., Ristanović, E., et al. (2022). Chemical and Biological Investigations of Allium scorodoprasum L. Flower Extracts. *Pharmaceuticals* 16, 21. doi: 10.3390/ph16010021  dos Santos, F. A. R., Xavier, J. A., da Silva, F. C., Merlin, J. P. J., Goulart, M. O. F., and Rupasinghe, H. P. V. (2022). Antidiabetic, Antiglycation, and Antioxidant Activities of Ethanolic Seed Extract of Passiflora edulis and Piceatannol In Vitro. *Molecules* 27, 4064. doi: 10.3390/molecules27134064  Eawsakul, K., Panichayupakaranant, P., Ongtanasup, T., Warinhomhoun, S., Noonong, K., and Bunluepuech, K. (2021). Computational study and in vitro alpha-glucosidase inhibitory effects of medicinal plants from a Thai folk remedy. *Heliyon* 7, e08078. doi: 10.1016/j.heliyon.2021.e08078  El-Askary, H., Salem, H. H., and Abdel Motaal, A. (2022). Potential Mechanisms Involved in the Protective Effect of Dicaffeoylquinic Acids from Artemisia annua L. Leaves against Diabetes and Its Complications. *Molecules* 27, 857. doi: 10.3390/molecules27030857  Encarnação, S., De Mello-Sampayo, C., Carrapiço, B., São Braz, B., Jordão, A., Peleteiro, C., et al. (2022). Anacardium occidentale Bark as an Antidiabetic Agent. *Plants* 11, 2637. doi: 10.3390/plants11192637  Erukainure, O. L., Oyebode, O. A., Salau, V. F., Koorbanally, N. A., and Islam, M. S. (2019). Flowers of Clerodendrum volubile modulates redox homeostasis and suppresses DNA fragmentation in Fe2+ − induced oxidative hepatic and pancreatic injuries; and inhibits carbohydrate catabolic enzymes linked to type 2 diabetes. *J. Diabetes Metab. Disord.* 18, 513–524. doi: 10.1007/s40200-019-00458-3  Erukainure, O. L., Ijomone, O. M., Chukwuma, C. I., Xiao, X., Salau, V. F., and Islam, M. S. (2020). Dacryodes edulis (G. Don) H.J. Lam modulates glucose metabolism, cholinergic activities and Nrf2 expression, while suppressing oxidative stress and dyslipidemia in diabetic rats. *J. Ethnopharmacol.* 255, 112744. doi: 10.1016/j.jep.2020.112744  Erukainure, O. L., Salau, V. F., Xiao, X., Matsabisa, M. G., Koorbanally, N. A., and Islam, M. S. (2021). Bioactive compounds of African star apple ( Chrysophyllum albidum G. Don) and its modulatory effect on metabolic activities linked to type 2 diabetes in isolated rat psoas muscle. *J. Food Biochem.* 45. doi: 10.1111/jfbc.13576  Fagbohunka, B., Nwolisah, O., Odufuwa, K., and Adetayo, P. (2024). In vitro study of the inhibitory potentials of cold and hot aqueous extract of Vernonia amygdalina, Calotropis procera, and Persea americana on α-glucosidase. *Ann. Heal. Res. (The J. Med. Dent. Consult. Assoc. Niger. OOUTH, Sagamu, Niger.* 10, 152–162. doi: 10.30442/ahr.1002-07-235  Fawzi Mahomoodally, M., Picot-Allain, M. C. N., Zengin, G., Llorent-Martínez, E. J., Abdullah, H. H., Ak, G., et al. (2020). Phytochemical Analysis, Network Pharmacology and in Silico Investigations on Anacamptis pyramidalis Tuber Extracts. *Molecules* 25, 2422. doi: 10.3390/molecules25102422  Ferron, L., Colombo, R., Mannucci, B., and Papetti, A. (2020). A New Italian Purple Corn Variety (Moradyn) Byproduct Extract: Antiglycative and Hypoglycemic In Vitro Activities and Preliminary Bioaccessibility Studies. *Molecules* 25, 1958. doi: 10.3390/molecules25081958  Fettach, S., Mrabti, H. N., Sayah, K., Bouyahya, A., Salhi, N., Cherrah, Y., et al. (2019). Phenolic content, acute toxicity of Ajuga iva extracts and assessment of their antioxidant and carbohydrate digestive enzyme inhibitory effects. *South African J. Bot.* 125, 381–385. doi: 10.1016/j.sajb.2019.08.010  Finos, M. B., Visentini, F. F., Cian, R., Fernández, J. L., Santiago, L. G., and Perez, A. A. (2024). Nanosupplements based on protein-polysaccharide coacervates loaded with essential oils: Evaluation of antioxidant and antidiabetic properties. *Food Hydrocoll.* 155, 110248. doi: 10.1016/j.foodhyd.2024.110248  Floris, S., Pintus, F., Fais, A., Era, B., Raho, N., Siguri, C., et al. (2024). Biological Potential of Asphodelus microcarpus Extracts: α-Glucosidase and Antibiofilm Activities In Vitro. *Molecules* 29, 5063. doi: 10.3390/molecules29215063  Gamboa‐Gómez, C. I., Guerrero‐Romero, F., Sánchez‐Meraz, M. A., and Simental‐Mendía, L. E. (2020). Hypoglycemic and antioxidant properties of konjac ( Amorphophallus konjac ) in vitro and in vivo. *J. Food Biochem.* 44. doi: 10.1111/jfbc.13503  Giles-Rivas, D., Estrada-Soto, S., Aguilar-Guadarrama, A. B., Almanza-Pérez, J., García-Jiménez, S., Colín-Lozano, B., et al. (2020). Antidiabetic effect of Cordia morelosana, chemical and pharmacological studies. *J. Ethnopharmacol.* 251, 112543. doi: 10.1016/j.jep.2020.112543  Giri, B. R., Baral, R., Bhatt, H., Khadka, A., Tamrakar, R., Timalsina, G., et al. (2023). Phytochemical Screening, Free-Radical Scavenging Activity, in vitro Alpha-Amylase Inhibitory Activity, and in vivo Hypoglycemic Activity Studies of Several Crude Drug Formulations Based on Selected Medicinal Plants of Nepal. *Pharm. Chem. J.* 56, 1369–1378. doi: 10.1007/s11094-023-02799-z  Gunny, A. A. N., Subramanian, P., Mahmod, S. S., AL-Rajabi, M. M., Ahmad, A. A., and Abu Bakar, A. R. (2024). Mechanism of inhibition of alpha-amylase by caffeic acid using in-vitro and in-silico techniques. *Nat. Prod. Res.*, 1–5. doi: 10.1080/14786419.2024.2402465  Haddou, S., Elrherabi, A., Loukili, E. H., Abdnim, R., Hbika, A., Bouhrim, M., et al. (2023). Chemical Analysis of the Antihyperglycemic, and Pancreatic α-Amylase, Lipase, and Intestinal α-Glucosidase Inhibitory Activities of Cannabis sativa L. Seed Extracts. *Molecules* 29, 93. doi: 10.3390/molecules29010093  Haguet, Q., Le Joubioux, F., Chavanelle, V., Groult, H., Schoonjans, N., Langhi, C., et al. (2023). Inhibitory Potential of α-Amylase, α-Glucosidase, and Pancreatic Lipase by a Formulation of Five Plant Extracts: TOTUM-63. *Int. J. Mol. Sci.* 24, 3652. doi: 10.3390/ijms24043652  Halayal, R. Y., Bagewadi, Z. K., Aldabaan, N. A., Shaikh, I. A., and Khan, A. A. (2024). Exploring the therapeutic mechanism of potential phytocompounds from Kalanchoe pinnata in the treatment of diabetes mellitus by integrating network pharmacology, molecular docking and simulation approach. *Saudi Pharm. J.* 32, 102026. doi: 10.1016/j.jsps.2024.102026  Heyem, Z., Nassima, B., Ahmed, M., Erenler, R., Lahcene, Z., Fadila, B., et al. (2022). Phytochemical Profile, Anti-lipid peroxidation and Anti-diabetic activities of Thymus algeriensis Boiss. &amp; Reut. *Egypt. J. Chem.*, 0–0. doi: 10.21608/ejchem.2022.126336.5600  Huang, T., Liu, P., Lin, Y., and Tsai, J. (2022). Hypoglycemic peptide‐enriched hydrolysates of <scp> *Corbicula fluminea* </scp> and <scp> *Chlorella sorokiniana* </scp> possess synergistic hypoglycemic activity through inhibiting α‐glucosidase and dipeptidyl peptidase‐4 activity. *J. Sci. Food Agric.* 102, 716–723. doi: 10.1002/jsfa.11402  Hussain, Z. T. E., Yagi, S., Mahomoodally, M. F., Mohammed, I., and Zengin, G. (2019). A comparative study of different solvents and extraction techniques on the anti-oxidant and enzyme inhibitory activities of Adansonia digitata L. (Baobab) fruit pulp. *South African J. Bot.* 126, 207–213. doi: 10.1016/j.sajb.2019.01.034  I.M, A. T. I. ; A. R. O. ; A. M. A. ; D. U. C. ; B. I. D. ; O. A.-Q. K. ; A. (2023). Prediction of Antidiabetic Compounds in Curcuma longa – In vitro and In silico Investigations. *Trop. J. Nat. Prod. Res.* 7. doi: 10.26538/tjnpr/v7i10.33  Iheagwam, F. N., Israel, E. N., Kayode, K. O., DeCampos, O. C., Ogunlana, O. O., and Chinedu, S. N. (2020). Nauclea latifolia Sm. Leaf Extracts Extenuates Free Radicals, Inflammation, and Diabetes-Linked Enzymes. *Oxid. Med. Cell. Longev.* 2020, 1–13. doi: 10.1155/2020/5612486  Iraji, A., Saeedi, M., Rafiee-Sereshky, T., Mojtabavi, S., Faramarzi, M. A., and Akbarzadeh, T. (2022). Ugi Adducts: Design and Synthesis of Natural-based α-glucosidase Inhibitors. *Lett. Org. Chem.* 19, 1084–1093. doi: 10.2174/1570178619666220401143634  Ismail, H., Mohamed, A. I., and Islam, M. S. (2024). Aloe ferox leaf gel extracts attenuate redox imbalance in oxidative renal injury and stimulates glucose uptake, whilst inhibiting key enzymes linked to diabetes and obesity. *Sci. African* 26, e02425. doi: 10.1016/j.sciaf.2024.e02425  J, M., H, M., E, N., and K.M, G. (2022). Synergistic Effect of Ocimum sanctum and Piper nigrum: An In Vitro Study on Type 2 Diabetes-Related Enzymes and MCF-7 Breast Cancer Cell line. *Curr. Trends Biotechnol. Pharm.* 16, 56–63. doi: 10.5530/ctbp.2022.2s.31  J, D., V, V. P., R, G., and R, S. (2019). In vitro α-amylase and α-glucosidase inhibitory activity of iridoid glucoside. *Drug Invent. Today* 12, 1307.  Jacob, B., RT, N., Nadar, M. M., and Itsaranuwat, P. (2022). Mineral composition, phytochemical analysis, anti-oxidant and anti-diabetic activities of a polyherbal formulation- an in vitro approach. *Chem. Data Collect.* 39, 100874. doi: 10.1016/j.cdc.2022.100874  Jaishree, V., and Narsimha, S. (2020). Swertiamarin and quercetin combination ameliorates hyperglycemia, hyperlipidemia and oxidative stress in streptozotocin-induced type 2 diabetes mellitus in wistar rats. *Biomed. Pharmacother.* 130, 110561. doi: 10.1016/j.biopha.2020.110561  Jamir, L., and P., H. (2024). Employing Machine Learning Models to Predict Potential α-Glucosidase Inhibitory Plant Secondary Metabolites Targeting Type-2 Diabetes and Their In Vitro Validation. *J. Chem. Inf. Model.* 64, 9150–9162. doi: 10.1021/acs.jcim.4c00955  Jasim, A. R. M., Abhirami, B. L., Anto, E. M., George, S., Jayamurthy, P., and Kumaran, A. (2024). Multimodal therapeutic amelioration of type 2 diabetes via bioactive compounds isolated from Cassia mimosoides L. *South African J. Bot.* 172, 567–578. doi: 10.1016/j.sajb.2024.07.060  Jyothi Reddy, G., Bhaskar Reddy, K., and Subba Reddy, G. V. (2020). In vivo Anti-diabetic and Anti-hyperlipidemic Activities of ethyl Acetate/Methanol Fractions of Feronia elephantum Fruit in type 2 Diabetic Rats: Via α-amylase and PPAR-γ by using in silico Approach. *Indian J. Pharm. Educ. Res.* 54, 761–770. doi: 10.5530/ijper.54.3.128  Kajszczak, D., Sosnowska, D., Frąszczak, B., and Podsędek, A. (2024). Composition, Anti-Diabetic, and Antioxidant Potential of Raphanus sativus Leaves. *Molecules* 29, 5689. doi: 10.3390/molecules29235689  Khan, H. A., Ghufran, M., Shams, S., Jamal, A., Ayaz, M., Ullah, M., et al. (2023). In-depth in-vitro and in-vivo anti-diabetic evaluations of Fagonia cretica mediated biosynthesized selenium nanoparticles. *Biomed. Pharmacother.* 164, 114872. doi: 10.1016/j.biopha.2023.114872  Khan, D., Sharif, A., Zafar, M., Akhtar, B., Akhtar, M. F., and Awan, S. (2020). Delonix regia a Folklore Remedy for Diabetes; Attenuates Oxidative Stress and Modulates Type II Diabetes Mellitus. *Curr. Pharm. Biotechnol.* 21, 1059–1069. doi: 10.2174/1389201021666200217112244  Khojah, A. A., Padilla-González, G. F., Bader, A., Simmonds, M. J. S., Munday, M., and Heinrich, M. (2021). Barbeya oleoides Leaves Extracts: In Vitro Carbohydrate Digestive Enzymes Inhibition and Phytochemical Characterization. *Molecules* 26, 6229. doi: 10.3390/molecules26206229  Khusnutdinova, E. F., Petrova, A. V., Thu, H. N. T., Tu, A. L. T., Thanh, T. N., Thi, C. B., et al. (2019). Structural modifications of 2,3-indolobetulinic acid: Design and synthesis of highly potent α-glucosidase inhibitors. *Bioorg. Chem.* 88, 102957. doi: 10.1016/j.bioorg.2019.102957  Kiage-Mokua, B. N., De Vrese, M., Kraus-Stojanowic, I., Nielsen, A., Kareru, P., Kenji, G., et al. (2020). Effect of extracts from selected Kenyan plants on traits of metabolic syndrom in Wistar rats fed a high-fat high fructose diet. *Trop. J. Pharm. Res.* 19, 2137–2146. doi: 10.4314/tjpr.v19i10.18  Kikuchi, H., Toyoda, N., Ezawa, S., Yoshida, S., Hibino, Y., and Sunaga, K. (2020). Effects of hot‑water extracts from 26�herbs on α‑glucosidase activity. *Mol. Med. Rep.* doi: 10.3892/mmr.2020.11397  Kim, H.-R., Antonisamy, P., Kim, Y.-S., Lee, G., Ham, H.-D., and Kwon, K.-B. (2022). Inhibitory effect of Amomum villosum water extracts on α-glucosidase activity. *Physiol. Mol. Plant Pathol.* 117, 101779. doi: 10.1016/j.pmpp.2021.101779  Kısa, D., Kaya, Z., İmamoğlu, R., Genç, N., Taslimi, P., and Taskin-Tok, T. (2022). Assessment of antimicrobial and enzymes inhibition effects of Allium kastambulense with in silico studies: Analysis of its phenolic compounds and flavonoid contents. *Arab. J. Chem.* 15, 103810. doi: 10.1016/j.arabjc.2022.103810  Kukavica, B., Škondrić, S., Trifković, T., Mišić, D., Gašić, U., Topalić-Trivunović, L., et al. (2024). Comparative polyphenolic profiling of five ethnomedicinal plants and their applicative potential in the treatment of type 2 diabetes. *J. Ethnopharmacol.* 320, 117377. doi: 10.1016/j.jep.2023.117377  Lakshmi MV; Swapna, T. S. (2022). Bioactive molecules and the antidiabetic efficacy of <em>Memecylon randerianum</em> — an ethnomedicinal plant from the Western Ghats. *Indian J. Exp. Biol.* doi: 10.56042/ijeb.v59i12.57830  Laraoui, H., Lanez, E., Zegheb, N., Adaika, A., Lanez, T., and Benkhaled, M. (2023). Anti‐Diabetic Activity of Flavonol Glucosides From Fumana montana Pomel: In vitro Analysis, In Silico Docking, ADMET Prediction, and Molecular Dynamics Simulations. *ChemistrySelect* 8. doi: 10.1002/slct.202204512  Latolla, N., Reddy, S., Venter, M. van de, and Hlangothi, B. (2023). Phytochemical composition and antidiabetic potential of the leaf, stem, and rhizome extracts of Cissampelos capensis L.f. *South African J. Bot.* 163, 468–477. doi: 10.1016/j.sajb.2023.11.003  Leonardo, J., Putra, P. P., Tallei, T. E., Fatimawali, F., Taslim, N. A., Tjandrawinata, R. R., et al. (2024). Unraveling biomolecules, antidiabetic and antioxidants properties of DelitesTM via pharmacoinformatics and in vitro investigation. *Pharmacol. Res. - Mod. Chinese Med.* 13, 100551. doi: 10.1016/j.prmcm.2024.100551  Leporini, M., Loizzo, M. R., Sicari, V., Pellicanò, T. M., Reitano, A., Dugay, A., et al. (2020). Citrus × Clementina Hort. Juice Enriched with Its By-Products (Peels and Leaves): Chemical Composition, In Vitro Bioactivity, and Impact of Processing. *Antioxidants* 9, 298. doi: 10.3390/antiox9040298  Lévuok-Mena, K. P., Patiño-Ladino, O. J., and Prieto-Rodríguez, J. A. (2023). In Vitro Inhibitory Activities against α-Glucosidase, α-Amylase, and Pancreatic Lipase of Medicinal Plants Commonly Used in Chocó (Colombia) for Type 2 Diabetes and Obesity Treatment. *Sci. Pharm.* 91, 49. doi: 10.3390/scipharm91040049  Li, H., Zhai, B., Sun, J., Fan, Y., Zou, J., Cheng, J., et al. (2022). Ultrasound-Assisted Extraction of Total Saponins from Aralia taibaiensis: Process Optimization, Phytochemical Characterization, and Mechanism of α-Glucosidase Inhibition. *Drug Des. Devel. Ther.* Volume 16, 83–105. doi: 10.2147/DDDT.S345592  Li, D., Sun, L., Yang, Y., Wang, Z., Yang, X., Zhao, T., et al. (2019). Young apple polyphenols postpone starch digestion in vitro and in vivo. *J. Funct. Foods* 56, 127–135. doi: 10.1016/j.jff.2019.03.009  Li, C., Zhang, K., Liu, L., Shen, J., Wang, Y., Tan, Y., et al. (2023). Study of the Mechanism of Astragali Radix in Treating Type 2 Diabetes Mellitus and Its Renal Protection Based on Enzyme Activity, Network Pharmacology, and Experimental Verification. *Molecules* 28, 8030. doi: 10.3390/molecules28248030  Liu, Y., Zheng, W., Zhong, Y., Zhang, L., Su, T., Liang, G., et al. (2021). Identification of α-glucosidase inhibitors from Cortex Lycii based on a bioactivity-labeling high-resolution mass spectrometry–metabolomics investigation. *J. Chromatogr. A* 1642, 462041. doi: 10.1016/j.chroma.2021.462041  Lüersen, K., Fischer, A., Bauer, I., Huebbe, P., Uekaji, Y., Chikamoto, K., et al. (2023). Soy Extract, Rich in Hydroxylated Isoflavones, Exhibits Antidiabetic Properties In Vitro and in Drosophila melanogaster In Vivo. *Nutrients* 15, 1392. doi: 10.3390/nu15061392  Mabate, B., Daub, C. D., Malgas, S., Edkins, A. L., and Pletschke, B. I. (2021). A Combination Approach in Inhibiting Type 2 Diabetes-Related Enzymes Using Ecklonia radiata Fucoidan and Acarbose. *Pharmaceutics* 13, 1979. doi: 10.3390/pharmaceutics13111979  Mahdi, S., Azzi, R., and Lahfa, F. B. (2020). Evaluation of in vitro α-amylase and α-glucosidase inhibitory potential and hemolytic effect of phenolic enriched fractions of the aerial part of Salvia officinalis L. *Diabetes Metab. Syndr. Clin. Res. Rev.* 14, 689–694. doi: 10.1016/j.dsx.2020.05.002  Mahnashi, M. H., Alqahtani, Y. S., Alyami, B. A., Alqarni, A. O., Ayaz, M., Ghufran, M., et al. (2022). Phytochemical Analysis, α-Glucosidase and Amylase Inhibitory, and Molecular Docking Studies on Persicaria hydropiper L. Leaves Essential Oils. *Evidence-Based Complement. Altern. Med.* 2022, 1–11. doi: 10.1155/2022/7924171  Mala, P., Khan, G. A., Gopalan, R., Gedefaw, D., and Soapi, K. (2022). Fijian medicinal plants and their role in the prevention of Type 2 diabetes mellitus. *Biosci. Rep.* 42. doi: 10.1042/BSR20220461  Manzano, J. A. H., Llames, L. C. J., and Macabeo, A. P. G. (2023). Tetrahydrobisbenzylisoquinoline alkaloids from Phaeanthus ophthalmicus inhibit target enzymes associated with type 2 diabetes and obesity. *J. Appl. Pharm. Sci.* doi: 10.7324/JAPS.2023.154518  Martini, S., Solieri, L., Cattivelli, A., Pizzamiglio, V., and Tagliazucchi, D. (2021). An Integrated Peptidomics and In Silico Approach to Identify Novel Anti-Diabetic Peptides in Parmigiano-Reggiano Cheese. *Biology (Basel).* 10, 563. doi: 10.3390/biology10060563  Matowane, G. R., Ramorobi, L. M., Mashele, S. S., Bonnet, S. L., Noreljaleel, A. E. M., Swain, S. S., et al. (2023). Novel Caffeic Acid - Zinc Acetate Complex: Studies on Promising Antidiabetic and Antioxidative Synergism Through Complexation. *Med. Chem. (Los. Angeles).* 19, 147–162. doi: 10.2174/1573406418666220620144601  Mekki, S., Belhocine, M., Bouzouina, M., Chaouad, B., and Mostari, A. (2023). Therapeutic effects of Salvia balansae on metabolic disorders and testicular dysfunction mediated by a high-fat diet in Wistar rats. *Med. J. Nutrition Metab.* 16, 21–39. doi: 10.3233/MNM-220094  Meng, X., Zong, H., Zheng, Z., Xing, J., Liu, Z., Song, F., et al. (2023). Ligand-targeted fishing of α-glucosidase inhibitors from Tribulus terrestris L. based on chitosan-functionalized multi-walled carbon nanotubes with immobilized α-glucosidase. *Anal. Bioanal. Chem.* 415, 2677–2692. doi: 10.1007/s00216-023-04666-y  Mirab, B., Ahmadi Gavlighi, H., Amini Sarteshnizi, R., Azizi, M. H., and C. Udenigwe, C. (2020). Production of low glycemic potential sponge cake by pomegranate peel extract (PPE) as natural enriched polyphenol extract: Textural, color and consumer acceptability. *LWT* 134, 109973. doi: 10.1016/j.lwt.2020.109973  Mohammed, H. S., Abdel-Aziz, M. M., Abu-Baker, M. S., Saad, A. M., Mohamed, M. A., and Ghareeb, M. A. (2019). Antibacterial and Potential Antidiabetic Activities of Flavone C-glycosides Isolated from Beta vulgaris Subspecies cicla L. var. Flavescens (Amaranthaceae) Cultivated in Egypt. *Curr. Pharm. Biotechnol.* 20, 595–604. doi: 10.2174/1389201020666190613161212  Morais, F. S., Canuto, K. M., Ribeiro, P. R. V., Silva, A. B., Pessoa, O. D. L., Freitas, C. D. T., et al. (2020). Chemical profiling of secondary metabolites from Himatanthus drasticus (Mart.) Plumel latex with inhibitory action against the enzymes α-amylase and α-glucosidase: In vitro and in silico assays. *J. Ethnopharmacol.* 253, 112644. doi: 10.1016/j.jep.2020.112644  Msomi, N. Z., Shode, F. O., Pooe, O. J., Mazibuko-Mbeje, S., and Simelane, M. B. C. (2019). Iso-Mukaadial Acetate from Warburgia salutaris Enhances Glucose Uptake in the L6 Rat Myoblast Cell Line. *Biomolecules* 9, 520. doi: 10.3390/biom9100520  Mugaranja, K. P., and Kulal, A. (2022). Investigation of effective natural inhibitors for starch hydrolysing enzymes from Simaroubaceae plants by molecular docking analysis and comparison with in-vitro studies. *Heliyon* 8, e09360. doi: 10.1016/j.heliyon.2022.e09360  Mugari, P., Nyoni, S., and Dzomba, P. (2024). Ethnomedicinal Plants, Associated Indigenous Knowledge and Phytochemical Composition of Extracts with Significant in vitro Antidiabetic Activity. *Pharmacognosy Res.* 16, 769–783. doi: 10.5530/pres.16.4.89  Mukundh, S. T., Veeraraghavan, V. P., Ponnusamy, B., and Jayaraman, S. (2024). Phytochemical Screening and Antidiabetic Activity of Aqueous Extract of Evolvulus Alsinoides Leaves: An In Vitro and In Silico Study. *J. Pharm. Bioallied Sci.* 16, S1246–S1248. doi: 10.4103/jpbs.jpbs_585_23  Musa, A., Ahmed, S. R., Hussein, S., Youssif, K. A., El-Ghorab, A. H., Haidari, R. A. Al, et al. (2024). Prominent antidiabetic and anticancer investigation of Scrophularia deserti extract: Integration of experimental and computational approaches. *J. Mol. Struct.* 1315, 138769. doi: 10.1016/j.molstruc.2024.138769  Nadeem, M., Mumtaz, M. W., Danish, M., Rashid, U., Mukhtar, H., and Irfan, A. (2020). Antidiabetic functionality of Vitex negundo L. leaves based on UHPLC-QTOF-MS/MS based bioactives profiling and molecular docking insights. *Ind. Crops Prod.* 152, 112445. doi: 10.1016/j.indcrop.2020.112445  Nag, S., Stany, B., Mishra, S., Kumar, S., Mohanto, S., Ahmed, M. G., et al. (2024). Multireceptor Analysis for Evaluating the Antidiabetic Efficacy of Karanjin: A Computational Approach. *Endocrinol. Diabetes Metab.* 7. doi: 10.1002/edm2.509  Nakashima, Y., Yamamoto, N., Tsukioka, R., Sugawa, H., Ohshima, R., Aoki, K., et al. (2022). In vitro evaluation of the anti-diabetic potential of soymilk yogurt and identification of inhibitory compounds on the formation of advanced glycation end-products. *Food Biosci.* 50, 102051. doi: 10.1016/j.fbio.2022.102051  Naz, D., Muhamad, A., Zeb, A., and Shah, I. (2019). In vitro and in vivo Antidiabetic Properties of Phenolic Antioxidants From Sedum adenotrichum. *Front. Nutr.* 6. doi: 10.3389/fnut.2019.00177  Ndarawit, W., Ochieng, C. O., Angwenyi, D., Cruz, J. N., Santos, C. B. R., and Kimani, N. M. (2024). Discovery of α-amylase and α-glucosidase dual inhibitors from NPASS database for management of Type 2 Diabetes Mellitus: A chemoinformatic approach. *PLoS One* 19, e0313758. doi: 10.1371/journal.pone.0313758  Ng, Z. X., and See, A. N. (2019). Effect of in vitro digestion on the total polyphenol and flavonoid, antioxidant activity and carbohydrate hydrolyzing enzymes inhibitory potential of selected functional plant‐based foods. *J. Food Process. Preserv.* 43, e13903. doi: 10.1111/jfpp.13903  Ngo, D.-H., Ngo, D.-N., Vo, T. T. N., and Vo, T. S. (2019). Mechanism of Action of Mangifera indica Leaves for Anti-Diabetic Activity. *Sci. Pharm.* 87, 13. doi: 10.3390/scipharm87020013  Nipun, T. S., Khatib, A., Ibrahim, Z., Ahmed, Q. U., Redzwan, I. E., Saiman, M. Z., et al. (2020). Characterization of α-Glucosidase Inhibitors from Psychotria malayana Jack Leaves Extract Using LC-MS-Based Multivariate Data Analysis and In-Silico Molecular Docking. *Molecules* 25, 5885. doi: 10.3390/molecules25245885  Nur Kabidul Azam, M., Biswas, P., Mohaimenul Islam Tareq, M., Ridoy Hossain, M., Bibi, S., Anisul Hoque, M., et al. (2024). Identification of antidiabetic inhibitors from Allophylus villosus and Mycetia sinensis by targeting α-glucosidase and PPAR-γ: In-vitro, in-vivo, and computational evidence. *Saudi Pharm. J.* 32, 101884. doi: 10.1016/j.jsps.2023.101884  Nyathi, B., Bvunzawabaya, J. T., Venissa P Mudawarima, C., Manzombe, E., Tsotsoro, K., Selemani, M. A., et al. (2023). Inhibitory and in silico molecular docking of Xeroderris stuhlmannii (Taub.) Mendonca &amp; E.P. Sousa phytochemical compounds on human α-glucosidases. *J. Ethnopharmacol.* 312, 116501. doi: 10.1016/j.jep.2023.116501  Ogbe, A. A., Gupta, S., Finnie, J. F., and van Staden, J. (2023). Preliminary studies on in vitro antioxidant and retardation of essential carbohydrate hydrolysing enzymes by some indigenous South African medicinal plants. *South African J. Bot.* 159, 686–696. doi: 10.1016/j.sajb.2023.05.030  Ogbe, A. A., Naidoo, D., Kar, P., Roy, A., Finnie, J. F., and Van Staden, J. (2024). The hypoglycemic potential of Syzygium cordatum (Hochst. ex Krauss.) extracts; in vitro analysis and in silico modelling. *South African J. Bot.* 174, 239–248. doi: 10.1016/j.sajb.2024.08.046  Ojo, O. A., Gyebi, G. A., Ezenabor, E. H., Iyobhebhe, M., Emmanuel, D. A., Adelowo, O. A., et al. (2024). Exploring beetroot ( Beta vulgaris L.) for diabetes mellitus and Alzheimer’s disease dual therapy: in vitro and computational studies. *RSC Adv.* 14, 19362–19380. doi: 10.1039/D4RA03638G  Oyebode, O. A., Erukainure, O. L., Ibeji, C. U., Koorbanally, N. A., and Islam, M. S. (2019). Phytochemical constituents, antioxidant and antidiabetic activities of different extracts of the leaves, stem and root barks of Alstonia boonei : an in vitro and in silico study. *Bot. Lett.* 166, 444–456. doi: 10.1080/23818107.2019.1624980  Oyebode, O., Erukainure, O. L., Zuma, L., Ibeji, C. U., Koorbanally, N. A., and Islam, M. S. (2022). In vitro and computational studies of the antioxidant and anti-diabetic properties of Bridelia ferruginea. *J. Biomol. Struct. Dyn.* 40, 3989–4003. doi: 10.1080/07391102.2020.1852961  Oyedemi, S. O., Atanes, P., Aiyegoro, O. A., Amoo, S. O., Swain, S. S., and Persaud, S. J. (2023). In vitro profiling and functional assessments of the anti‐diabetic capacity of phenolic‐rich extracts of Bulbine natalensis and Bulbine frutescens. *Diabet. Med.* 40. doi: 10.1111/dme.14770  Parveen, S., Shehzadi, S., Shafiq, N., Rashid, M., Naz, S., Mehmood, T., et al. (2025). A discovery of potent kaempferol derivatives as multi-target medicines against diabetes as well as bacterial infections: an in silico approach. *J. Biomol. Struct. Dyn.* 43, 5218–5240. doi: 10.1080/07391102.2024.2308773  Perumal, N., Nallappan, M., Shohaimi, S., Kassim, N. K., Tee, T. T., and Cheah, Y. H. (2022). Synergistic antidiabetic activity of Taraxacum officinale (L.) Weber ex F.H.Wigg and Momordica charantia L. polyherbal combination. *Biomed. Pharmacother.* 145, 112401. doi: 10.1016/j.biopha.2021.112401  Placines, C., Castaneda-Loaiza, V., Rodrigues, M. J., Pereira, C. G., da Silva, J. P., Zengin, G., et al. (2021). In Vitro Enzyme Inhibitory and Antioxidant Properties, Cytotoxicity, and LC-DAD-ESI-MS/MS Profile of Extracts from the Halophyte Lotus creticus L. *Jundishapur J. Nat. Pharm. Prod.* 16. doi: 10.5812/jjnpp.101125  Prasetyawan, S., Safitri, A., and Rahayu, S. (2022). Computational study of Curcuma zanthorrhiza Roxb compounds as potential antidiabetic towards alpha-amylase, alpha-glucosidase, and Keap1 inhibition. *J. Pharm. Pharmacogn. Res.* 10, 206–217. doi: 10.56499/jppres21.1175_10.2.206  Purnomo, Y., Makdasari, J., and Fatahillah, F. I. (2021). Inhibitory activity of Urena lobata leaf extract on alpha-amylase and alpha-glucosidase: in vitro and in silico approach. *J. Basic Clin. Physiol. Pharmacol.* 32, 889–894. doi: 10.1515/jbcpp-2020-0430  Qi, S., Jiang, B., Huang, C., and Jin, Y. (2023). Dual Regulation of Sulfonated Lignin to Prevent and Treat Type 2 Diabetes Mellitus. *Biomacromolecules* 24, 841–848. doi: 10.1021/acs.biomac.2c01267  Qi, J., Wang, D., Yin, X., Zhang, Q., and Gao, J.-M. (2020). New Metabolite With Inhibitory Activity Against α-Glucosidase and α-Amylase From Endophytic Chaetomium globosum. *Nat. Prod. Commun.* 15. doi: 10.1177/1934578X20941338  Qin, C., Chen, N., and Li, J. (2024). Detailed investigation on the polyphenols from four Dendrobium species against α-glucosidase: An integrated in vitro and in silico approach. *Arab. J. Chem.* 17, 105627. doi: 10.1016/j.arabjc.2024.105627  Quimque, M. T. J., Magsipoc, R. J. Y., Llames, L. C. J., Flores, A. I. G., Garcia, K. Y. M., Ratzenböck, A., et al. (2022). Polyoxygenated Cyclohexenes from Uvaria grandiflora with Multi-Enzyme Targeting Properties Relevant in Type 2 Diabetes and Obesity. *ACS Omega* 7, 36856–36864. doi: 10.1021/acsomega.2c05544  Quintero‐Soto, M. F., Chávez‐Ontiveros, J., Garzón‐Tiznado, J. A., Salazar‐Salas, N. Y., Pineda‐Hidalgo, K. V., Delgado‐Vargas, F., et al. (2021). Characterization of peptides with antioxidant activity and antidiabetic potential obtained from chickpea ( Cicer arietinum L.) protein hydrolyzates. *J. Food Sci.* 86, 2962–2977. doi: 10.1111/1750-3841.15778  R, S., V, V., D, Z.-D., V, B., I, S., S, Y., et al. (2019). Trans-3,5-dicaffeoylquinic acid from Geigeria alata Benth. &amp; Hook.f. ex Oliv. &amp; Hiern with beneficial effects on experimental diabetes in animal model of essential hypertension. *Food Chem. Toxicol.* 132, 110678. doi: 10.1016/j.fct.2019.110678  Rahman, S., Jan, G., and Jan, F. G. (2024). Isolation, Characterization, Chemical Profiling and Evaluation of Antidiabetic and Antioxidant Potential of Innula cappa (Buch.-Ham. ex D. Don) DC Leaves in Alloxan-Induced Type 2 Diabetic Mice. *Nat. Prod. Commun.* 19. doi: 10.1177/1934578X241306064  Rangra, N., Samanta, S., and Pradhan, K. (2021). Evaluation of Acacia auriculiformis Benth. leaves for wound healing activity in type 2 diabetic rats. *Pharmacogn. Mag.* 17, 129. doi: 10.4103/pm.pm_496_20  Rao, G., Yu, H., Zhang, M., Cheng, Y., Ran, K., Wang, J., et al. (2022). α-Glucosidase and Bacterial β-Glucuronidase Inhibitors from the Stems of Schisandra sphaerandra Staph. *Pharmaceuticals* 15, 329. doi: 10.3390/ph15030329  Rehman, A.-, Latif, A., Anwar, R., Abbas, N., Nawaz, S., and Mirza, H. T. (2019). Postprandial Anti-Diabetic Effects of Various Fractions of Fagonia indica Burm. f. by in vitro and in vivo Studies. *Pak. J. Zool.* 51. doi: 10.17582/journal.pjz/2019.51.1.333.340  Retnaningtyas, E., Setiawan, A., Susatia, B., Hariyanto, T., and Sudiwati, N. L. P. E. (2024). In silico studies of Ruellia tuberosa L. compounds as aldose reductase, dipeptidyl peptidase 4, and α-glucosidase inhibitors against type 2 diabetes mellitus. *J. Pharm. Pharmacogn. Res.* 12, 735–747. doi: 10.56499/jppres23.1891_12.4.735  Rodrigues, M. J., Jekő, J., Cziáky, Z., Pereira, C. G., and Custódio, L. (2022). The Medicinal Halophyte Frankenia laevis L. (Sea Heath) Has In Vitro Antioxidant Activity, α-Glucosidase Inhibition, and Cytotoxicity towards Hepatocarcinoma Cells. *Plants* 11, 1353. doi: 10.3390/plants11101353  Rodrigues, M. J., Oliveira, M., Neves, V., Ovelheiro, A., Pereira, C. A., Neng, N. R., et al. (2019). Coupling sea lavender (Limonium algarvense Erben) and green tea (Camellia sinensis (L.) Kuntze) to produce an innovative herbal beverage with enhanced enzymatic inhibitory properties. *South African J. Bot.* 120, 87–94. doi: 10.1016/j.sajb.2017.12.003  Rout, D., Dash, U. C., Kanhar, S., Swain, S. K., and Sahoo, A. K. (2022). Homalium zeylanicum attenuates streptozotocin-induced hyperglycemia and cellular stress in experimental rats via attenuation of oxidative stress imparts inflammation. *J. Ethnopharmacol.* 283, 114649. doi: 10.1016/j.jep.2021.114649  Royapuram Parthasarathy, P., E, I. V., and Shanmugam, R. (2023). In Vitro Anti-diabetic Activity of Pomegranate Peel Extract-Mediated Strontium Nanoparticles. *Cureus*. doi: 10.7759/cureus.51356  Sabiu, S., Balogun, F. O., and Amoo, S. O. (2021). Phenolics Profiling of Carpobrotus edulis (L.) N.E.Br. and Insights into Molecular Dynamics of Their Significance in Type 2 Diabetes Therapy and Its Retinopathy Complication. *Molecules* 26, 4867. doi: 10.3390/molecules26164867  Sajid, M., Khan, M. R., Ismail, H., Latif, S., Rahim, A. A., Mehboob, R., et al. (2020). Antidiabetic and antioxidant potential of Alnus nitida leaves in alloxan induced diabetic rats. *J. Ethnopharmacol.* 251, 112544. doi: 10.1016/j.jep.2020.112544  Salaj, N., Kladar, N., Srđenović Čonić, B., Jeremić, K., Hitl, M., Gavarić, N., et al. (2021). Traditional multi-herbal formula in diabetes therapy – Antihyperglycemic and antioxidant potential. *Arab. J. Chem.* 14, 103347. doi: 10.1016/j.arabjc.2021.103347  Salau, V. F., Erukainure, O. L., Koorbanally, N. A., and Islam, M. S. (2023). Kolaviron modulates dysregulated metabolism in oxidative pancreatic injury and inhibits intestinal glucose absorption with concomitant stimulation of muscle glucose uptake. *Arch. Physiol. Biochem.* 129, 157–167. doi: 10.1080/13813455.2020.1806331  Salau, V. F., Olofinsan, K. A., Mishra, A. P., Odewole, O. A., Ngnameko, C. R., and Matsabisa, M. G. (2024). Croton gratissimus Burch Herbal Tea Exhibits Anti-Hyperglycemic and Anti-Lipidemic Properties via Inhibition of Glycation and Digestive Enzyme Activities. *Plants* 13, 1952. doi: 10.3390/plants13141952  Sarker, P., Sani, D. H., Miah, M. F., and Alam, M. J. (2024). Curbing Key Digestive Enzymes by Three Plant Extracts for Sustainable Management of Postprandial Hyperglycemia. *Lett. Drug Des. Discov.* 21, 2015–2022. doi: 10.2174/1570180820666230518100900  Sato, V. H., Chewchinda, S., Goli, A. S., Sato, H., Nontakham, J., and Vongsak, B. (2023). Oral Glucose Tolerance Test (OGTT) Evidence for the Postprandial Anti-Hyperglycemic Property of Salacca zalacca (Gaertn.) Voss Seed Extract. *Molecules* 28, 6775. doi: 10.3390/molecules28196775  Sciacca, C., Cardullo, N., Pulvirenti, L., Travagliante, G., D’Urso, A., D’Agata, R., et al. (2024). Synthesis of obovatol and related neolignan analogues as α-glucosidase and α-amylase inhibitors. *Bioorg. Chem.* 147, 107392. doi: 10.1016/j.bioorg.2024.107392  Sekhon-Loodu, S., and Rupasinghe, H. P. V. (2019). Evaluation of Antioxidant, Antidiabetic and Antiobesity Potential of Selected Traditional Medicinal Plants. *Front. Nutr.* 6. doi: 10.3389/fnut.2019.00053  Shahid, M., Fazry, S., Azfaralariff, A., Najm, A. A. K., Law, D., and Mackeen, M. M. (2023). Bioactive compound identification and in vitro evaluation of antidiabetic and cytotoxic potential of Garcinia atroviridis fruit extract. *Food Biosci.* 51, 102285. doi: 10.1016/j.fbio.2022.102285  Shamim, T., Asif, H. M., Abida Ejaz, S., Hussain, Z., Wani, T. A., Sumreen, L., et al. (2024). Investigations of Limeum Indicum Plant for Diabetes Mellitus and Alzheimer’s Disease Dual Therapy: Phytochemical, GC‐MS Chemical Profiling, Enzyme Inhibition, Molecular Docking and In‐Vivo Studies. *Chem. Biodivers.* 21. doi: 10.1002/cbdv.202301858  Shen, Y., Wang, M., Zhou, J., Chen, Y., Wu, M., Yang, Z., et al. (2020). Construction of Fe3O4@α-glucosidase magnetic nanoparticles for ligand fishing of α-glucosidase inhibitors from a natural tonic Epimedii Folium. *Int. J. Biol. Macromol.* 165, 1361–1372. doi: 10.1016/j.ijbiomac.2020.10.018  Shen, B., Shangguan, X., Yin, Z., Wu, S., Zhang, Q., Peng, W., et al. (2021). Inhibitory Effect of Fisetin on α-Glucosidase Activity: Kinetic and Molecular Docking Studies. *Molecules* 26, 5306. doi: 10.3390/molecules26175306  Shojaeifard, Z., Moheimanian, N., and Jassbi, A. R. (2023). Comparison of inhibitory activities of 50 Salvia species against α-Glucosidase. *J. Diabetes Metab. Disord.* 22, 1685–1693. doi: 10.1007/s40200-023-01301-6  Siahbalaei, R., and Kavoosi, G. (2021). In Vitro Anti-diabetic Activity of Free Amino Acid and Protein Amino Acid Extracts from Four Iranian Medicinal Plants. *Iran. J. Sci. Technol. Trans. A Sci.* 45, 443–454. doi: 10.1007/s40995-020-01031-x  Sinan, K. I., Chiavaroli, A., Orlando, G., Bene, K., Zengin, G., Cziáky, Z., et al. (2020). Evaluation of Pharmacological and Phytochemical Profiles of Piptadeniastrum africanum (Hook.f.) Brenan Stem Bark Extracts. *Biomolecules* 10, 516. doi: 10.3390/biom10040516  Spínola, V., and Castilho, P. C. (2021). Assessing the In Vitro Inhibitory Effects on Key Enzymes Linked to Type-2 Diabetes and Obesity and Protein Glycation by Phenolic Compounds of Lauraceae Plant Species Endemic to the Laurisilva Forest. *Molecules* 26, 2023. doi: 10.3390/molecules26072023  Sravani, T., and Sunitha, K. (2024). Phytochemical Screening and α-Glucosidase Inhibitor Activity of Aerial Parts of Maidenhair Fern: *Adiantum lunulatum*. *J. Nat. Remedies*, 1355–1362. doi: 10.18311/jnr/2024/35850  Srisongkram, T., Waithong, S., Thitimetharoch, T., and Weerapreeyakul, N. (2022). Machine Learning and In Vitro Chemical Screening of Potential α-Amylase and α-Glucosidase Inhibitors from Thai Indigenous Plants. *Nutrients* 14, 267. doi: 10.3390/nu14020267  Stojkovic, D., Smiljkovic, M., Ciric, A., Glamoclija, J., Van Griensven, L., Ferreira, I. C. F. R., et al. (2019). An insight into antidiabetic properties of six medicinal and edible mushrooms: Inhibition of α-amylase and α-glucosidase linked to type-2 diabetes. *South African J. Bot.* 120, 100–103. doi: 10.1016/j.sajb.2018.01.007  Sun, K., Ding, M., Fu, C., Li, P., Li, T., Fang, L., et al. (2023). Effects of dietary wild bitter melon (Momordica charantia var. abbreviate Ser.) extract on glucose and lipid metabolism in HFD/STZ-induced type 2 diabetic rats. *J. Ethnopharmacol.* 306, 116154. doi: 10.1016/j.jep.2023.116154  Suryavanshi, A., Kumar, S., Kain, D., and Arya, A. (2021). Chemical composition, antioxidant and enzyme inhibitory properties of Ajuga parviflora Benth. *Biocatal. Agric. Biotechnol.* 37, 102191. doi: 10.1016/j.bcab.2021.102191  Swargiary, A., and Daimari, M. (2021). Identification of Major Compounds and α-Amylase and α-Glucosidase Inhibitory Activity of Rhizome of Musa balbisiana Colla: An in-vitro and in-silico Study. *Comb. Chem. High Throughput Screen.* 25, 139–148. doi: 10.2174/1386207323666201124144332  Świątek, Ł., Sieniawska, E., Sinan, K. I., Zengin, G., Boguszewska, A., Hryć, B., et al. (2023). Chemical Characterization of Different Extracts of Justicia secunda Vahl and Determination of Their Anti-Oxidant, Anti-Enzymatic, Anti-Viral, and Cytotoxic Properties. *Antioxidants* 12, 509. doi: 10.3390/antiox12020509  Syabana, M. A., Yuliana, N. D., Batubara, I., and Fardiaz, D. (2022). α-glucosidase inhibitors from Syzygium polyanthum (Wight) Walp leaves as revealed by metabolomics and in silico approaches. *J. Ethnopharmacol.* 282, 114618. doi: 10.1016/j.jep.2021.114618  Tang, H., Ma, F., Zhao, D., and Xue, Z. (2019). Exploring the effect of salvianolic acid C on α-glucosidase: Inhibition kinetics, interaction mechanism and molecular modelling methods. *Process Biochem.* 78, 178–188. doi: 10.1016/j.procbio.2019.01.011  Thissera, B., Visvanathan, R., Khanfar, M. A., Qader, M. M., Hassan, M. H. A., Hassan, H. M., et al. (2020). Sesbania grandiflora L. Poir leaves: A dietary supplement to alleviate type 2 diabetes through metabolic enzymes inhibition. *South African J. Bot.* 130, 282–299. doi: 10.1016/j.sajb.2020.01.011  Tiwari, V. P., Dubey, A., Al-Shehri, M., and Tripathi, I. P. (2024). Exploration of human pancreatic alpha-amylase inhibitors from Physalis peruviana for the treatment of type 2 diabetes. *J. Biomol. Struct. Dyn.* 42, 1031–1046. doi: 10.1080/07391102.2023.2243336  Tolmie, M., Bester, M. J., and Apostolides, Z. (2021). Inhibition of α‐glucosidase and α‐amylase by herbal compounds for the treatment of type 2 diabetes: A validation of in silico reverse docking with in vitro enzyme assays. *J. Diabetes* 13, 779–791. doi: 10.1111/1753-0407.13163  Trang, N. T. H., Tang, D. Y. Y., Chew, K. W., Linh, N. T., Hoang, L. T., Cuong, N. T., et al. (2021). Discovery of α-Glucosidase Inhibitors from Marine Microorganisms: Optimization of Culture Conditions and Medium Composition. *Mol. Biotechnol.* 63, 1004–1015. doi: 10.1007/s12033-021-00362-3  Trentin, R., Custódio, L., Rodrigues, M. J., Moschin, E., Sciuto, K., da Silva, J. P., et al. (2020). Exploring Ulva australis Areschoug for possible biotechnological applications: In vitro antioxidant and enzymatic inhibitory properties, and fatty acids contents. *Algal Res.* 50, 101980. doi: 10.1016/j.algal.2020.101980  Tundis, R., Grande, F., Occhiuzzi, M. A., Sicari, V., Loizzo, M. R., and Cappello, A. R. (2023). Lavandula angustifolia mill. (Lamiaceae) ethanol extract and its main constituents as promising agents for the treatment of metabolic disorders: chemical profile, in vitro biological studies, and molecular docking. *J. Enzyme Inhib. Med. Chem.* 38. doi: 10.1080/14756366.2023.2269481  Uddin, M. J., Faraone, I., Haque, M. A., Rahman, M. M., Halim, M. A., Sönnichsen, F. D., et al. (2022). Insights into the leaves of Ceriscoides campanulata: Natural proanthocyanidins alleviate diabetes, inflammation, and esophageal squamous cell cancer via in vitro and in silico models. *Fitoterapia* 158, 105164. doi: 10.1016/j.fitote.2022.105164  V, K., D, M., N, S., A, K., and J, K. (2023). Molecular docking analysis of natural compounds from medicinal plants against α-amylase and α-glucosidase of type 2 diabetes. *Int. J. Bioinform. Res. Appl.* 19, 280–305. doi: 10.1504/IJBRA.2023.135366  Vadivu, R. S., Bakthavatchalam, S., Rani, V. G., Hirad, A. H., Wen, Z.-H., Yuan, C.-H., et al. (2024). Assessment of anti-diabetic properties of Ziziphus oenopolia (L.) wild edible fruit extract: In vitro and in silico investigations through molecular docking analysis. *Open Chem.* 22. doi: 10.1515/chem-2024-0032  Vijh, D., and Gupta, P. (2024). GC–MS analysis, molecular docking, and pharmacokinetic studies on Dalbergia sissoo barks extracts for compounds with anti-diabetic potential. *Sci. Rep.* 14, 24936. doi: 10.1038/s41598-024-75570-3  Vo, T., Le, P., and Ngo, D. (2021). The Role of Physalis angulata as Potential Anti‑Type 2 Diabetic Agent. *Phcog Res* 13, 69–74. doi: 10.4103/pr.pr_51_19  Vo, T. S., Le, P. U., and Ngo, D.-H. (2022). In Vitro Hypoglycemic and Radical Scavenging Activities of Certain Medicinal Plants. *Serbian J. Exp. Clin. Res.* 23, 291–298. doi: 10.2478/sjecr-2019-0083  Wang, X., Deng, Y., Wang, J., Qin, L., Du, Y., Zhang, Q., et al. (2024). New natural protein tyrosine phosphatase 1B inhibitors from Gynostemma pentaphyllum. *J. Enzyme Inhib. Med. Chem.* 39. doi: 10.1080/14756366.2024.2360063  Wariyapperuma, W. A. N. M., Kannangara, S., Wijayasinghe, Y. S., Subramanium, S., and Jayawardena, B. (2020). In vitro anti-diabetic effects and phytochemical profiling of novel varieties of Cinnamomum zeylanicum (L.) extracts. *PeerJ* 8, e10070. doi: 10.7717/peerj.10070  Weng, L., Chen, T.-H., Zheng, Q., Weng, W.-H., Huang, L., Lai, D., et al. (2021). Syringaldehyde promoting intestinal motility with suppressing α-amylase hinders starch digestion in diabetic mice. *Biomed. Pharmacother.* 141, 111865. doi: 10.1016/j.biopha.2021.111865  Wong, P. Lou, Zolkeflee, N. K. Z., Ramli, N. S., Tan, C. P., Azlan, A., Tham, C. L., et al. (2024). Antidiabetic effect of Ardisia elliptica extract and its mechanisms of action in STZ-NA-induced diabetic rat model via 1H-NMR-based metabolomics. *J. Ethnopharmacol.* 318, 117015. doi: 10.1016/j.jep.2023.117015  Wongsa, P., Phatikulrungsun, P., and Prathumthong, S. (2022). FT-IR characteristics, phenolic profiles and inhibitory potential against digestive enzymes of 25 herbal infusions. *Sci. Rep.* 12, 6631. doi: 10.1038/s41598-022-10669-z  Wu, S., Dong, C., Zhang, M., Cheng, Y., Cao, X., Yang, B., et al. (2024). Revealing the Hypoglycemic Effect of Red Yeast Rice: Perspectives from the Inhibition of α-Glucosidase and the Anti-Glycation Capability by Ankaflavin and Monascin. *Foods* 13, 1573. doi: 10.3390/foods13101573  Xiao, X., Erukainure, O. L., Beseni, B., Koorbanally, N. A., and Islam, M. S. (2020). Sequential extracts of red honeybush ( Cyclopia genistoides ) tea: Chemical characterization, antioxidant potentials, and anti‐hyperglycemic activities. *J. Food Biochem.* 44. doi: 10.1111/jfbc.13478  Xu, X.-T., Deng, X.-Y., Chen, J., Liang, Q.-M., Zhang, K., Li, D.-L., et al. (2020). Synthesis and biological evaluation of coumarin derivatives as α-glucosidase inhibitors. *Eur. J. Med. Chem.* 189, 112013. doi: 10.1016/j.ejmech.2019.112013  Xu, J., Li, T., Xia, X., Fu, C., Wang, X., and Zhao, Y. (2020). Dietary Ginsenoside T19 Supplementation Regulates Glucose and Lipid Metabolism via AMPK and PI3K Pathways and Its Effect on Intestinal Microbiota. *J. Agric. Food Chem.* 68, 14452–14462. doi: 10.1021/acs.jafc.0c04429  Y, G., E, H., N, M., Z, L., J, F., and R, Y. (2020). Discovery, biological evaluation and docking studies of novel N-acyl-2-aminothiazoles fused (+)-nootkatone from Citrus paradisi Macf. as potential α-glucosidase inhibitors. *Bioorg. Chem.* 104, 104294. doi: 10.1016/j.bioorg.2020.104294  Y, C. A., R, H., D, S. P. A. E., F, S. S., O, S., P, A. L. Z., et al. (2020). In vitro Antihyperglycemic and Chelating Potential of Selected Ayurvedic Medicinal Plants. *Indian J. Pharm. Sci.* 82. doi: 10.36468/pharmaceutical-sciences.672  Zabidi, N. A., Ishak, N. A., Hamid, M., Ashari, S. E., and Mohammad Latif, M. A. (2021). Inhibitory evaluation of Curculigo latifolia on α-glucosidase, DPP (IV) and in vitro studies in antidiabetic with molecular docking relevance to type 2 diabetes mellitus. *J. Enzyme Inhib. Med. Chem.* 36, 109–121. doi: 10.1080/14756366.2020.1844680  Zeng, A., Yang, R., Yu, S., and Zhao, W. (2020). A novel hypoglycemic agent: polysaccharides from laver ( Porphyra spp.). *Food Funct.* 11, 9048–9056. doi: 10.1039/D0FO01195A  Zhang, P., Wei, W., Zhang, X., Wen, C., Ovatlarnporn, C., and Olatunji, O. J. (2023). Antidiabetic and antioxidant activities of Mitragyna speciosa (kratom) leaf extract in type 2 diabetic rats. *Biomed. Pharmacother.* 162, 114689. doi: 10.1016/j.biopha.2023.114689  Zhang, H., Che, X., Jing, H., Su, Y., Yang, W., Wang, R., et al. (2024). A New Potent Inhibitor against α-Glucosidase Based on an In Vitro Enzymatic Synthesis Approach. *Molecules* 29, 878. doi: 10.3390/molecules29040878 |
| Glucose absorption and uptake | Achyutha Devi, J., Bindu, G., and Ravi Kiran, S. (2023). Evaluation of in vitro glucose metabolism potential of sterols and essential oil extracts from Erythroxylum monogynum Roxb. using cell lines and enzyme inhibition models. *South African J. Bot.* 159, 110–130. doi: 10.1016/j.sajb.2023.06.008  Aladejana, A. E., Bradley, G., and Afolayan, A. J. (2021). In vitro evaluation of the anti-diabetic potential of Helichrysum petiolare Hilliard &amp; B.L. Burtt using HepG2 (C3A) and L6 cell lines. *F1000Research* 9, 1240. doi: 10.12688/f1000research.26855.2  Alsawalha, M., Al-Subaei, A., Al-Jindan, R., Bolla, S., Sen, D., Balakrishna, J., et al. (2019). Anti-diabetic activities of Dactylorhiza hatagirea leaf extract in 3T3-L1 cell line model. *Pharmacogn. Mag.* 15, 212. doi: 10.4103/pm.pm_8_19  Ansari, P., Flatt, P. R., Harriott, P., Hannan, J. M. A., and Abdel-Wahab, Y. H. A. (2021). Identification of Multiple Pancreatic and Extra-Pancreatic Pathways Underlying the Glucose-Lowering Actions of Acacia arabica Bark in Type-2 Diabetes and Isolation of Active Phytoconstituents. *Plants* 10, 1190. doi: 10.3390/plants10061190  Ansari, P., Hannan, J. M. A., Choudhury, S. T., Islam, S. S., Talukder, A., Seidel, V., et al. (2022). Antidiabetic Actions of Ethanol Extract of Camellia sinensis Leaf Ameliorates Insulin Secretion, Inhibits the DPP-IV Enzyme, Improves Glucose Tolerance, and Increases Active GLP-1 (7–36) Levels in High-Fat-Diet-Fed Rats. *Medicines* 9, 56. doi: 10.3390/medicines9110056  Awwad, A., Poucheret, P., Idres, Y. A., Tshibangu, D. S. T., Servent, A., Ferrare, K., et al. (2021). In Vitro Tests for a Rapid Evaluation of Antidiabetic Potential of Plant Species Containing Caffeic Acid Derivatives: A Validation by Two Well-Known Antidiabetic Plants, Ocimum gratissimum L. Leaf and Musanga cecropioides R. Br. ex Tedlie (Mu) Stem Bark. *Molecules* 26, 5566. doi: 10.3390/molecules26185566  Azimian, L., Weerasuriya, N. M., Munasinghe, R., Song, S., Lin, C., and You, L. (2023). Investigating the effects of Ceylon cinnamon water extract on HepG2 cells for Type 2 diabetes therapy. *Cell Biochem. Funct.* 41, 254–267. doi: 10.1002/cbf.3778  Bai, J., Zhang, S., Cao, J., Sun, H., Mang, Z., Shen, W. L., et al. (2022). Hernandezine, a natural herbal alkaloid, ameliorates type 2 diabetes by activating AMPK in two mouse models. *Phytomedicine* 105, 154366. doi: 10.1016/j.phymed.2022.154366  Bharadwaja, S., Issac, P. K., Cleta, J., Jeganathan, R., Chandrakumar, S. S., and Sundaresan, S. (2021). An in vitro mechanistic approach towards understanding the distinct pathways regulating insulin resistance and adipogenesis by apocynin. *J. Biosci.* 46. Available at: http://www.ncbi.nlm.nih.gov/pubmed/33709960  Borkar, R. M., Kanwal, A., Raju, B., Pulimamidi, S. S., Das, A. P., Agarwal, S. M., et al. (2023). A pharmacokinetic study to correlate the hypoglycemic effect of phlorizin in rats: Identification of metabolites as inhibitors of sodium/glucose cotransporters. *J. Mass Spectrom.* 58. doi: 10.1002/jms.4964  Casertano, M., Genovese, M., Piazza, L., Balestri, F., Del Corso, A., Vito, A., et al. (2022). Identifying Human PTP1B Enzyme Inhibitors from Marine Natural Products: Perspectives for Developing of Novel Insulin-Mimetic Drugs. *Pharmaceuticals* 15, 325. doi: 10.3390/ph15030325  Choi, E. M., Suh, K. S., Park, S. Y., Yun, S., Chin, S. O., Rhee, S. Y., et al. (2020). Orientin reduces the inhibitory effects of 2,3,7,8-tetrachlorodibenzo-p-dioxin on adipogenic differentiation and insulin signaling pathway in murine 3T3-L1 adipocytes. *Chem. Biol. Interact.* 318, 108978. doi: 10.1016/j.cbi.2020.108978  Chukiatsiri, S., Wongsrangsap, N., Ratanabunyong, S., and Choowongkomon, K. (2022). In Vitro Evaluation of Antidiabetic Potential of Cleistocalyx nervosum var. paniala Fruit Extract. *Plants* 12, 112. doi: 10.3390/plants12010112  Dhanya, R., and Jayamurthy, P. (2020). In vitro evaluation of antidiabetic potential of hesperidin and its aglycone hesperetin under oxidative stress in skeletal muscle cell line. *Cell Biochem. Funct.* 38, 419–427. doi: 10.1002/cbf.3478  Dound, Y. A., Chaudhary, S., Chaudhary, S. S., Rawat, S., Alqarni, M. H., Ahmad, M. M., et al. (2021). Mechanistic understanding of PtyroneTM: A plant based natural anti diabetic product. *J. King Saud Univ. - Sci.* 33, 101454. doi: 10.1016/j.jksus.2021.101454  Erukainure, O. L., Ijomone, O. M., Chukwuma, C. I., Xiao, X., Salau, V. F., and Islam, M. S. (2020). Dacryodes edulis (G. Don) H.J. Lam modulates glucose metabolism, cholinergic activities and Nrf2 expression, while suppressing oxidative stress and dyslipidemia in diabetic rats. *J. Ethnopharmacol.* 255, 112744. doi: 10.1016/j.jep.2020.112744  Erukainure, O. L., Salau, V. F., Xiao, X., Matsabisa, M. G., Koorbanally, N. A., and Islam, M. S. (2021). Bioactive compounds of African star apple ( Chrysophyllum albidum G. Don) and its modulatory effect on metabolic activities linked to type 2 diabetes in isolated rat psoas muscle. *J. Food Biochem.* 45. doi: 10.1111/jfbc.13576  Henaux, L., Pereira, K. D., Thibodeau, J., Pilon, G., Gill, T., Marette, A., et al. (2021). Glucoregulatory and Anti-Inflammatory Activities of Peptide Fractions Separated by Electrodialysis with Ultrafiltration Membranes from Salmon Protein Hydrolysate and Identification of Four Novel Glucoregulatory Peptides. *Membranes (Basel).* 11, 528. doi: 10.3390/membranes11070528  Ismail, H., Mohamed, A. I., and Islam, M. S. (2024). Aloe ferox leaf gel extracts attenuate redox imbalance in oxidative renal injury and stimulates glucose uptake, whilst inhibiting key enzymes linked to diabetes and obesity. *Sci. African* 26, e02425. doi: 10.1016/j.sciaf.2024.e02425  Jasim, A. R. M., Abhirami, B. L., Anto, E. M., George, S., Jayamurthy, P., and Kumaran, A. (2024). Multimodal therapeutic amelioration of type 2 diabetes via bioactive compounds isolated from Cassia mimosoides L. *South African J. Bot.* 172, 567–578. doi: 10.1016/j.sajb.2024.07.060  Kashyap, B., Barge, S. R., Bharadwaj, S., Deka, B., Rahman, S., Ghosh, A., et al. (2021). Evaluation of therapeutic effect of Premna herbacea in diabetic rat and isoverbascoside against insulin resistance in L6 muscle cells through bioenergetics and stimulation of JNK and AKT/mTOR signaling cascade. *Phytomedicine* 93, 153761. doi: 10.1016/j.phymed.2021.153761  Kumar, V., Sachan, R., Rahman, M., Sharma, K., Al-Abbasi, F. A., and Anwar, F. (2021). Prunus amygdalus extract exert antidiabetic effect via inhibition of DPP-IV: in-silico and in-vivo approaches. *J. Biomol. Struct. Dyn.* 39, 4160–4174. doi: 10.1080/07391102.2020.1775124  Lakshmi MV; Swapna, T. S. (2022). Bioactive molecules and the antidiabetic efficacy of <em>Memecylon randerianum</em> — an ethnomedicinal plant from the Western Ghats. *Indian J. Exp. Biol.* doi: 10.56042/ijeb.v59i12.57830  Lankatillake, C., Huynh, T., and Dias, D. A. (2024). Abrus precatorius Leaf Extract Stimulates Insulin-mediated Muscle Glucose Uptake: In vitro Studies and Phytochemical Analysis. *Planta Med.* 90, 388–396. doi: 10.1055/a-2281-0988  Matowane, G. R., Ramorobi, L. M., Mashele, S. S., Bonnet, S. L., Noreljaleel, A. E. M., Swain, S. S., et al. (2023). Novel Caffeic Acid - Zinc Acetate Complex: Studies on Promising Antidiabetic and Antioxidative Synergism Through Complexation. *Med. Chem. (Los. Angeles).* 19, 147–162. doi: 10.2174/1573406418666220620144601  Matsabisa, M. G., Chukwuma, C. I., Chaudhary, S. K., Kumar, C. S., Baleni, R., Javu, M., et al. (2020). Dicoma anomala (Sond.) abates glycation and DPP-IV activity and modulates glucose utilization in Chang liver cells and 3T3-L1 adipocytes. *South African J. Bot.* 128, 182–188. doi: 10.1016/j.sajb.2019.09.013  Moens, C., Muller, C. J. F., and Bouwens, L. (2022). In vitro comparison of various antioxidants and flavonoids from Rooibos as beta cell protectants against lipotoxicity and oxidative stress-induced cell death. *PLoS One* 17, e0268551. doi: 10.1371/journal.pone.0268551  Mrabti, H. N., El Abbes Faouzi, M., Mayuk, F. M., Makrane, H., Limas-Nzouzi, N., Dibong, S. D., et al. (2019). Arbutus unedo L., (Ericaceae) inhibits intestinal glucose absorption and improves glucose tolerance in rodents. *J. Ethnopharmacol.* 235, 385–391. doi: 10.1016/j.jep.2019.02.013  Msomi, N. Z., Shode, F. O., Pooe, O. J., Mazibuko-Mbeje, S., and Simelane, M. B. C. (2019). Iso-Mukaadial Acetate from Warburgia salutaris Enhances Glucose Uptake in the L6 Rat Myoblast Cell Line. *Biomolecules* 9, 520. doi: 10.3390/biom9100520  Ngo, D.-H., Ngo, D.-N., Vo, T. T. N., and Vo, T. S. (2019). Mechanism of Action of Mangifera indica Leaves for Anti-Diabetic Activity. *Sci. Pharm.* 87, 13. doi: 10.3390/scipharm87020013  Ni, D., Ai, Z., Munoz‐Sandoval, D., Suresh, R., Ellis, P. R., Yuqiong, C., et al. (2020). Inhibition of the facilitative sugar transporters (GLUTs) by tea extracts and catechins. *FASEB J.* 34, 9995–10010. doi: 10.1096/fj.202000057RR  Nishikai-Shen, T., Hosono-Fukao, T., Ariga, T., Hosono, T., and Seki, T. (2022). Cinnamon extract improves abnormalities in glucose tolerance by decreasing Acyl-CoA synthetase long-chain family 1 expression in adipocytes. *Sci. Rep.* 12, 12574. doi: 10.1038/s41598-022-13421-9  Olusola, A. J., Famuyiwa, S. O., Faloye, K. O., Olatunji, O. E., Olayemi, U. I., Adeyemi, A. A., et al. (2024). Neomangiferin, a Naturally Occurring Mangiferin Congener, Inhibits Sodium-Glucose Co-transporter-2: An In silico Approach. *Bioinform. Biol. Insights* 18. doi: 10.1177/11779322231223851  Ovalle-Magallanes, B., Navarrete, A., Haddad, P. S., Tovar, A. R., Noriega, L. G., Tovar-Palacio, C., et al. (2019). Multi-target antidiabetic mechanisms of mexicanolides from Swietenia humilis. *Phytomedicine* 58, 152891. doi: 10.1016/j.phymed.2019.152891  Oyebode, O. A., Erukainure, O. L., Chuturgoon, A. A., Ghazi, T., Naidoo, P., Chukwuma, C. I., et al. (2022). Bridelia ferruginea Benth. (Euphorbiaceae) mitigates oxidative imbalance and lipotoxicity, with concomitant modulation of insulin signaling pathways via GLUT4 upregulation in hepatic tissues of diabetic rats. *J. Ethnopharmacol.* 284, 114816. doi: 10.1016/j.jep.2021.114816  Peng, S., Wang, Y., Zhou, Y., Ma, T., Wang, Y., Li, J., et al. (2019). Rare ginsenosides ameliorate lipid overload-induced myocardial insulin resistance via modulating metabolic flexibility. *Phytomedicine* 58, 152745. doi: 10.1016/j.phymed.2018.11.006  Perumal, N., Nallappan, M., Shohaimi, S., Kassim, N. K., Tee, T. T., and Cheah, Y. H. (2022). Synergistic antidiabetic activity of Taraxacum officinale (L.) Weber ex F.H.Wigg and Momordica charantia L. polyherbal combination. *Biomed. Pharmacother.* 145, 112401. doi: 10.1016/j.biopha.2021.112401  Salau, V. F., Erukainure, O. L., Bharuth, V., Ibeji, C. U., Olasehinde, T. A., and Islam, M. S. (2021). Kolaviron stimulates glucose uptake with concomitant modulation of metabolic activities implicated in neurodegeneration in isolated rat brain, without perturbation of tissue ultrastructural morphology. *Neurosci. Res.* 169, 57–68. doi: 10.1016/j.neures.2020.06.008  Salau, V. F., Erukainure, O. L., Koorbanally, N. A., and Islam, M. S. (2023). Kolaviron modulates dysregulated metabolism in oxidative pancreatic injury and inhibits intestinal glucose absorption with concomitant stimulation of muscle glucose uptake. *Arch. Physiol. Biochem.* 129, 157–167. doi: 10.1080/13813455.2020.1806331  Schreck, K., and Melzig, M. F. (2021). Traditionally Used Plants in the Treatment of Diabetes Mellitus: Screening for Uptake Inhibition of Glucose and Fructose in the Caco2-Cell Model. *Front. Pharmacol.* 12. doi: 10.3389/fphar.2021.692566  Singh, D., Bedi, N., Tiwary, A. K., Kurmi, B. Das, and Bhattacharya, S. (2022). Natural bio functional lipids containing solid self-microemulsifying drug delivery system of Canagliflozin for synergistic prevention of type 2 diabetes mellitus. *J. Drug Deliv. Sci. Technol.* 69, 103138. doi: 10.1016/j.jddst.2022.103138  Stadlbauer, V., Neuhauser, C., Aumiller, T., Stallinger, A., Iken, M., and Weghuber, J. (2021). Identification of Insulin-Mimetic Plant Extracts: From an In Vitro High-Content Screen to Blood Glucose Reduction in Live Animals. *Molecules* 26, 4346. doi: 10.3390/molecules26144346  Su, H., Xie, L., Xu, Y., Ke, H., Bao, T., Li, Y., et al. (2020). Pelargonidin-3- O -glucoside Derived from Wild Raspberry Exerts Antihyperglycemic Effect by Inducing Autophagy and Modulating Gut Microbiota. *J. Agric. Food Chem.* 68, 13025–13037. doi: 10.1021/acs.jafc.9b03338  Surbala, L., Singh, C. B., Devi, R. V., and Singh, O. J. (2020). Rutaecarpine exhibits anti-diabetic potential in high fat diet–multiple low dose streptozotocin induced type 2 diabetic mice and in vitro by modulating hepatic glucose homeostasis. *J. Pharmacol. Sci.* 143, 307–314. doi: 10.1016/j.jphs.2020.04.008  Suriyah, W. H., Ichwan, S. J. A., Kasmuri, A. R., and Taher, M. (2019). In vitro Evaluation of the Effect of Pluchea indica Extracts in Promoting Glucose Consumption Activity on A Liver Cell Line. *Makara J. Heal. Res.* 23. doi: 10.7454/msk.v23i1.10153  Suryavanshi, A., Kumar, S., Kain, D., and Arya, A. (2021). Chemical composition, antioxidant and enzyme inhibitory properties of Ajuga parviflora Benth. *Biocatal. Agric. Biotechnol.* 37, 102191. doi: 10.1016/j.bcab.2021.102191  Viraragavan, A., Hlengwa, N., de Beer, D., Riedel, S., Miller, N., Bowles, S., et al. (2020). Model development for predicting in vitro bio-capacity of green rooibos extract based on composition for application as screening tool in quality control. *Food Funct.* 11, 3084–3094. doi: 10.1039/C9FO02480H  Vo, T. S., Le, P. U., and Ngo, D.-H. (2022). In Vitro Hypoglycemic and Radical Scavenging Activities of Certain Medicinal Plants. *Serbian J. Exp. Clin. Res.* 23, 291–298. doi: 10.2478/sjecr-2019-0083  Vo, T., Le, P., and Ngo, D. (2021). The Role of Physalis angulata as Potential Anti‑Type 2 Diabetic Agent. *Phcog Res* 13, 69–74. doi: 10.4103/pr.pr_51_19  Wang, Y., Zeng, T., Li, H., Wang, Y., Wang, J., and Yuan, H. (2023). Structural Characterization and Hypoglycemic Function of Polysaccharides from Cordyceps cicadae. *Molecules* 28, 526. doi: 10.3390/molecules28020526  Yoon, S.-Y., Ahn, D., Hwang, J. Y., Kang, M. J., and Chung, S. J. (2021). Linoleic acid exerts antidiabetic effects by inhibiting protein tyrosine phosphatases associated with insulin resistance. *J. Funct. Foods* 83, 104532. doi: 10.1016/j.jff.2021.104532  Yoon, S.-Y., Kim, J., Lee, B. S., Baek, S. C., Chung, S. J., and Kim, K. H. (2022). Terminalin from African Mango (Irvingia gabonensis) Stimulates Glucose Uptake through Inhibition of Protein Tyrosine Phosphatases. *Biomolecules* 12, 321. doi: 10.3390/biom12020321  Zabidi, N. A., Ishak, N. A., Hamid, M., Ashari, S. E., and Mohammad Latif, M. A. (2021). Inhibitory evaluation of Curculigo latifolia on α-glucosidase, DPP (IV) and in vitro studies in antidiabetic with molecular docking relevance to type 2 diabetes mellitus. *J. Enzyme Inhib. Med. Chem.* 36, 109–121. doi: 10.1080/14756366.2020.1844680  Zhao, P., Zhong, S., Liao, J., Tao, J., Yao, Y., Song, P., et al. (2025). Caragana jubata ethanol extract ameliorates the symptoms of STZ-HFD-induced T2DM mice by PKC/GLUT4 pathway. *J. Ethnopharmacol.* 339, 119171. doi: 10.1016/j.jep.2024.119171 |
| Glucose storage | de Godoi, R. S., Almerão, M. P., and da Silva, F. R. (2021). In silico evaluation of the antidiabetic activity of natural compounds from Hovenia dulcis Thunberg. *J. Herb. Med.* 28, 100349. doi: 10.1016/j.hermed.2020.100349 |
| Glucose production inhibition | Andrade-Cetto, A., Espinoza-Hernández, F., and Mata-Torres, G. (2021). Hypoglycemic Effect of Calea urticifolia (Mill.) DC. *Evidence-Based Complement. Altern. Med.* 2021, 1–10. doi: 10.1155/2021/6625009  Andrade-Cetto, A., Espinoza-Hernández, F., Mata-Torres, G., and Escandón-Rivera, S. (2021). Hypoglycemic Effect of Two Mexican Medicinal Plants. *Plants* 10, 2060. doi: 10.3390/plants10102060  Awwad, A., Poucheret, P., Idres, Y. A., Tshibangu, D. S. T., Servent, A., Ferrare, K., et al. (2021). In Vitro Tests for a Rapid Evaluation of Antidiabetic Potential of Plant Species Containing Caffeic Acid Derivatives: A Validation by Two Well-Known Antidiabetic Plants, Ocimum gratissimum L. Leaf and Musanga cecropioides R. Br. ex Tedlie (Mu) Stem Bark. *Molecules* 26, 5566. doi: 10.3390/molecules26185566  Ayyoub, S., Al-Trad, B., Aljabali, A. A. A., Alshaer, W., Al Zoubi, M., Omari, S., et al. (2022). Biosynthesis of gold nanoparticles using leaf extract of Dittrichia viscosa and in vivo assessment of its anti-diabetic efficacy. *Drug Deliv. Transl. Res.* 12, 2993–2999. doi: 10.1007/s13346-022-01163-0  Brás, N. F., Neves, R. P. P., Lopes, F. A. A., Correia, M. A. S., Palma, A. S., Sousa, S. F., et al. (2021). Combined in silico and in vitro studies to identify novel antidiabetic flavonoids targeting glycogen phosphorylase. *Bioorg. Chem.* 108, 104552. doi: 10.1016/j.bioorg.2020.104552  Chetter, B. A., Kyriakis, E., Barr, D., Karra, A. G., Katsidou, E., Koulas, S. M., et al. (2020). Synthetic flavonoid derivatives targeting the glycogen phosphorylase inhibitor site: QM/MM-PBSA motivated synthesis of substituted 5,7-dihydroxyflavones, crystallography, in vitro kinetics and ex-vivo cellular experiments reveal novel potent inhibitors. *Bioorg. Chem.* 102, 104003. doi: 10.1016/j.bioorg.2020.104003  Drakou, C. E., Gardeli, C., Tsialtas, I., Alexopoulos, S., Mallouchos, A., Koulas, S. M., et al. (2020). Affinity Crystallography Reveals Binding of Pomegranate Juice Anthocyanins at the Inhibitor Site of Glycogen Phosphorylase: The Contribution of a Sugar Moiety to Potency and Its Implications to the Binding Mode. *J. Agric. Food Chem.* 68, 10191–10199. doi: 10.1021/acs.jafc.0c04205  Erukainure, O. L., Oyebode, O. A., Salau, V. F., Koorbanally, N. A., and Islam, M. S. (2019). Flowers of Clerodendrum volubile modulates redox homeostasis and suppresses DNA fragmentation in Fe2+ − induced oxidative hepatic and pancreatic injuries; and inhibits carbohydrate catabolic enzymes linked to type 2 diabetes. *J. Diabetes Metab. Disord.* 18, 513–524. doi: 10.1007/s40200-019-00458-3  Henaux, L., Pereira, K. D., Thibodeau, J., Pilon, G., Gill, T., Marette, A., et al. (2021). Glucoregulatory and Anti-Inflammatory Activities of Peptide Fractions Separated by Electrodialysis with Ultrafiltration Membranes from Salmon Protein Hydrolysate and Identification of Four Novel Glucoregulatory Peptides. *Membranes (Basel).* 11, 528. doi: 10.3390/membranes11070528  Liu, S., Huang, S., Wu, X., Feng, Y., Shen, Y., Zhao, Q., et al. (2020). Activation of SIK1 by phanginin A inhibits hepatic gluconeogenesis by increasing PDE4 activity and suppressing the cAMP signaling pathway. *Mol. Metab.* 41, 101045. doi: 10.1016/j.molmet.2020.101045  Macalalad, M. A. B., and Gonzales, A. A. (2023). In Silico Screening and Identification of Antidiabetic Inhibitors Sourced from Phytochemicals of Philippine Plants against Four Protein Targets of Diabetes (PTP1B, DPP-4, SGLT-2, and FBPase). *Molecules* 28, 5301. doi: 10.3390/molecules28145301  Mata-Torres, G., Andrade-Cetto, A., Espinoza-Hernández, F. A., and Cárdenas-Vázquez, R. (2020). Hepatic Glucose Output Inhibition by Mexican Plants Used in the Treatment of Type 2 Diabetes. *Front. Pharmacol.* 11. doi: 10.3389/fphar.2020.00215  Ovalle-Magallanes, B., Navarrete, A., Haddad, P. S., Tovar, A. R., Noriega, L. G., Tovar-Palacio, C., et al. (2019). Multi-target antidiabetic mechanisms of mexicanolides from Swietenia humilis. *Phytomedicine* 58, 152891. doi: 10.1016/j.phymed.2019.152891  Pasachan, T., Duangjai, A., Ontawong, A., Amornlerdpison, D., Jinakote, M., Phatsara, M., et al. (2021). Tiliacora triandra (Colebr.) Diels Leaf Aqueous Extract Inhibits Hepatic Glucose Production in HepG2 Cells and Type 2 Diabetic Rats. *Molecules* 26, 1239. doi: 10.3390/molecules26051239  Patil, R. B., Owoseeni, O. D., Phage, P. M., Famuyiwa, S. O., Gboyero, F. O., Arowojolu, G. M., et al. (2024). In silico Identification of Fructose-1,6-biphosphatase Inhibitory Potentials of Xanthones Isolated from African Medicinal Plants: An Integrated Computational Approach. *Lett. Drug Des. Discov.* 21, 1675–1693. doi: 10.2174/1570180820666230417124235  Romo-Pérez, A., Escandón-Rivera, S. M., Miranda, L. D., and Andrade-Cetto, A. (2022). Phytochemical Study of Eryngium cymosum F. Delaroche and the Inhibitory Capacity of Its Main Compounds on Two Glucose-Producing Pathway Enzymes. *Plants* 11, 992. doi: 10.3390/plants11070992 |
| Insulin resistance driving factors | Abdel-Rahman, R. F., Ezzat, S. M., Ogaly, H. A., Abd-Elsalam, R. M., Hessin, A. F., Fekry, M. I., et al. (2020). Ficus deltoidea extract down-regulates protein tyrosine phosphatase 1B expression in a rat model of type 2 diabetes mellitus: a new insight into its antidiabetic mechanism. *J. Nutr. Sci.* 9, e2. doi: 10.1017/jns.2019.40  Ansari, P., Flatt, P. R., Harriott, P., and Abdel-Wahab, Y. H. A. (2021). Anti-hyperglycaemic and insulin-releasing effects of Camellia sinensis leaves and isolation and characterisation of active compounds. *Br. J. Nutr.* 126, 1149–1163. doi: 10.1017/S0007114520005085  Bharadwaja, S., Issac, P. K., Cleta, J., Jeganathan, R., Chandrakumar, S. S., and Sundaresan, S. (2021). An in vitro mechanistic approach towards understanding the distinct pathways regulating insulin resistance and adipogenesis by apocynin. *J. Biosci.* 46. Available at: http://www.ncbi.nlm.nih.gov/pubmed/33709960  Boonphang, O., Ontawong, A., Pasachan, T., Phatsara, M., Duangjai, A., Amornlerdpison, D., et al. (2021). Antidiabetic and Renoprotective Effects of Coffea arabica Pulp Aqueous Extract through Preserving Organic Cation Transport System Mediated Oxidative Stress Pathway in Experimental Type 2 Diabetic Rats. *Molecules* 26, 1907. doi: 10.3390/molecules26071907  Cai, J., Zhang, J., Li, S., Lin, Y., Xiao, X., and Guo, J. (2021). Comprehensive chemical analysis of Zhenshu Tiaozhi formula and its effect on ameliorating glucolipid metabolic disorders in diabetic rats. *Biomed. Pharmacother.* 133, 111060. doi: 10.1016/j.biopha.2020.111060  Campos, M. L. de, Castro, M. B. de, Campos, A. D., Fernandes, M. F., Conegundes, J. L. M., Rodrigues, M. N., et al. (2021). Antiobesity, hepatoprotective and anti-hyperglycemic effects of a pharmaceutical formulation containing Cecropia pachystachya Trécul in mice fed with a hypercaloric diet. *J. Ethnopharmacol.* 280, 114418. doi: 10.1016/j.jep.2021.114418  Chen, X., Chen, C., and Fu, X. (2022). Hypoglycemic activity in vitro and vivo of a water-soluble polysaccharide from Astragalus membranaceus. *Food Funct.* 13, 11210–11222. doi: 10.1039/D2FO02298B  de Campos Zani, S. C., Wang, R., Veida-Silva, H., Clugston, R. D., Yue, J. T. Y., Mori, M. A., et al. (2023). An Egg White-Derived Peptide Enhances Systemic Insulin Sensitivity and Modulates Markers of Non-Alcoholic Fatty Liver Disease in Obese, Insulin Resistant Mice. *Metabolites* 13, 174. doi: 10.3390/metabo13020174  De la Fuente-Muñoz, M., De la Fuente-Fernández, M., Román-Carmena, M., Amor, S., Iglesias-de la Cruz, M. C., García-Laínez, G., et al. (2023). Supplementation with a New Standardized Extract of Green and Black Tea Exerts Antiadipogenic Effects and Prevents Insulin Resistance in Mice with Metabolic Syndrome. *Int. J. Mol. Sci.* 24, 8521. doi: 10.3390/ijms24108521  Deng, X., Huang, S.-L., Ren, J., Pan, Z.-H., Shen, Y., Zhou, H.-F., et al. (2022). Development and structure–activity relationships of tanshinones as selective 11β-hydroxysteroid dehydrogenase 1 inhibitors. *Nat. Products Bioprospect.* 12, 36. doi: 10.1007/s13659-022-00358-9  Duan, J., Zhao, Y., Pei, F., Deng, W., He, L., Rao, C., et al. (2023). Swietenine inhibited oxidative stress through <scp>AKT</scp> /Nrf2/ <scp>HO</scp> ‐1 signal pathways and the liver‐protective effect in <scp>T2DM</scp> mice: In vivo and in vitro study. *Environ. Toxicol.* 38, 1292–1304. doi: 10.1002/tox.23764  Erukainure, O. L., Oyebode, O. A., Chuturgoon, A. A., Ghazi, T., Muhammad, A., Aljoundi, A., et al. (2024). Potential molecular mechanisms underlying the ameliorative effect of Cola nitida (Vent.) Schott &amp; Endl. on insulin resistance in rat skeletal muscles. *J. Ethnopharmacol.* 319, 117249. doi: 10.1016/j.jep.2023.117249  Guo, F., Yao, L., Zhang, W., Chen, P., Hao, R., Huang, X., et al. (2023). The therapeutic mechanism of Yuye decoction on type 2 diabetes mellitus based on network pharmacology and experimental verification. *J. Ethnopharmacol.* 308, 116222. doi: 10.1016/j.jep.2023.116222  Guo, S., Ouyang, H., Du, W., Li, J., Liu, M., Yang, S., et al. (2021). Exploring the protective effect of Gynura procumbens against type 2 diabetes mellitus by network pharmacology and validation in C57BL/KsJ db/db mice. *Food Funct.* 12, 1732–1744. doi: 10.1039/D0FO01188F  Hao, Y., Cui, W., Gao, H., Wang, M., Liu, Y., Li, C., et al. (2022). Jinlida granules ameliorate the high-fat-diet induced liver injury in mice by antagonising hepatocytes pyroptosis. *Pharm. Biol.* 60, 274–281. doi: 10.1080/13880209.2022.2029501  Huang, T., Liu, P., Lin, Y., and Tsai, J. (2022). Hypoglycemic peptide‐enriched hydrolysates of <scp> *Corbicula fluminea* </scp> and <scp> *Chlorella sorokiniana* </scp> possess synergistic hypoglycemic activity through inhibiting α‐glucosidase and dipeptidyl peptidase‐4 activity. *J. Sci. Food Agric.* 102, 716–723. doi: 10.1002/jsfa.11402  Jeong, H., Yang, D., Zhao, J., Seo, J. H., Shin, D. G., Cha, J.-D., et al. (2021). Ethanol Extract of Orostachys japonicus A. Berger (Crassulaceae) Protects Against Type 2 Diabetes by Reducing Insulin Resistance and Hepatic Inflammation in Mice. *J. Med. Food* 24, 464–478. doi: 10.1089/jmf.2020.4790  Ji, S., Zhu, C., Gao, S., Shao, X., Chen, X., Zhang, H., et al. (2021). Morus alba leaves ethanol extract protects pancreatic islet cells against dysfunction and death by inducing autophagy in type 2 diabetes. *Phytomedicine* 83, 153478. doi: 10.1016/j.phymed.2021.153478  Jiang, P., Zheng, W., Sun, X., Jiang, G., Wu, S., Xu, Y., et al. (2021). Sulfated polysaccharides from Undaria pinnatifida improved high fat diet-induced metabolic syndrome, gut microbiota dysbiosis and inflammation in BALB/c mice. *Int. J. Biol. Macromol.* 167, 1587–1597. doi: 10.1016/j.ijbiomac.2020.11.116  Karim, N., Rahman, A., Chanudom, L., Thongsom, M., and Tangpong, J. (2019). Mangosteen Vinegar Rind from Garcinia mangostana Prevents High‐Fat Diet and Streptozotocin‐Induced Type II Diabetes Nephropathy and Apoptosis. *J. Food Sci.* 84, 1208–1215. doi: 10.1111/1750-3841.14511  Kashyap, B., Barge, S. R., Bharadwaj, S., Deka, B., Rahman, S., Ghosh, A., et al. (2021). Evaluation of therapeutic effect of Premna herbacea in diabetic rat and isoverbascoside against insulin resistance in L6 muscle cells through bioenergetics and stimulation of JNK and AKT/mTOR signaling cascade. *Phytomedicine* 93, 153761. doi: 10.1016/j.phymed.2021.153761  Kiage-Mokua, B. N., De Vrese, M., Kraus-Stojanowic, I., Nielsen, A., Kareru, P., Kenji, G., et al. (2020). Effect of extracts from selected Kenyan plants on traits of metabolic syndrom in Wistar rats fed a high-fat high fructose diet. *Trop. J. Pharm. Res.* 19, 2137–2146. doi: 10.4314/tjpr.v19i10.18  Kien, D. X., Ha, D. V., Kien, P. T., Huy, T. H. A., Nga, N. T., Dung, V. M., et al. (2024). Blood Glucose Lowering Effect of Lagerstroemia speciosa L. Leaves Extract on Type 2 Diabetic Rat Model. *Trop. J. Nat. Prod. Res.* 8, 7709–7714. doi: 10.26538/tjnpr/v8i7.13  Lam, C.-S., Xia, Y.-X., Chen, B.-S., Du, Y.-X., Liu, K.-L., and Zhang, H.-J. (2023). Dihydro-Resveratrol Attenuates Oxidative Stress, Adipogenesis and Insulin Resistance in In Vitro Models and High-Fat Diet-Induced Mouse Model via AMPK Activation. *Nutrients* 15, 3006. doi: 10.3390/nu15133006  Lee, J. H., Park, J. E., and Han, J. S. (2020). Portulaca oleracea L. extract reduces hyperglycemia via PI3k/Akt and AMPK pathways in the skeletal muscles of C57BL/Ksj-db/db mice. *J. Ethnopharmacol.* 260, 112973. doi: 10.1016/j.jep.2020.112973  Li, C., Zhang, K., Liu, L., Shen, J., Wang, Y., Tan, Y., et al. (2023). Study of the Mechanism of Astragali Radix in Treating Type 2 Diabetes Mellitus and Its Renal Protection Based on Enzyme Activity, Network Pharmacology, and Experimental Verification. *Molecules* 28, 8030. doi: 10.3390/molecules28248030  Liu, Y., Zheng, S., Cui, J., Guo, T., and Zhang, J. (2022). Lactiplantibacillus plantarum Y15 alleviate type 2 diabetes in mice via modulating gut microbiota and regulating NF-κB and insulin signaling pathway. *Brazilian J. Microbiol.* 53, 935–945. doi: 10.1007/s42770-022-00686-5  Lyu, K., Yue, W., Ran, J., Liu, Y., and Zhu, X. (2021). In vivo therapeutic exploring for Mori folium extract against type 2 diabetes mellitus in rats. *Biosci. Rep.* 41. doi: 10.1042/BSR20210977  Meléndez-Martínez, D., Ortega-Hernández, E., Reza-Zaldívar, E. E., Carbajal-Saucedo, A., Arnaud-Franco, G., Gatica-Colima, A., et al. (2024). Bioprospection of rattlesnake venom peptide fractions with anti-adipose and anti-insulin resistance activity in vitro. *Toxicon X* 24, 100209. doi: 10.1016/j.toxcx.2024.100209  Meng, Q., Qi, X., Fu, Y., Chen, Q., Cheng, P., Yu, X., et al. (2020). Flavonoids extracted from mulberry (Morus alba L.) leaf improve skeletal muscle mitochondrial function by activating AMPK in type 2 diabetes. *J. Ethnopharmacol.* 248, 112326. doi: 10.1016/j.jep.2019.112326  Niu, D., An, S., Chen, X., Bi, H., Zhang, Q., Wang, T., et al. (2020). Corni Fructus as a Natural Resource Can Treat Type 2 Diabetes by Regulating Gut Microbiota. *Am. J. Chin. Med.* 48, 1385–1407. doi: 10.1142/S0192415X20500688  Olofinsan, K., Salau, V., Erukainure, O., and Islam, M. S. (2024). Erythrina lysistemon Hutch. abates deranged metabolic indices linked with diabetic complications in experimental rat model. *Sci. African* 26, e02478. doi: 10.1016/j.sciaf.2024.e02478  Oyebode, O., Zuma, L., Lucky Erukainure, O., Koorbanally, N., and Islam, M. S. (2023). Bridelia ferruginea inhibits key carbohydrate digesting enzyme and intestinal glucose absorption and modulates glucose metabolism in diabetic rats. *Arch. Physiol. Biochem.* 129, 671–681. doi: 10.1080/13813455.2020.1861026  Paul, K., Chakraborty, S., Mallick, P., Bhattacharjee, P., Pal, T. K., Chatterjee, N., et al. (2021). Supercritical carbon dioxide extracts of small cardamom and yellow mustard seeds have fasting hypoglycaemic effects: diabetic rat, predictive iHOMA2 models and molecular docking study. *Br. J. Nutr.* 125, 377–388. doi: 10.1017/S000711452000286X  Peng, S., Wang, Y., Zhou, Y., Ma, T., Wang, Y., Li, J., et al. (2019). Rare ginsenosides ameliorate lipid overload-induced myocardial insulin resistance via modulating metabolic flexibility. *Phytomedicine* 58, 152745. doi: 10.1016/j.phymed.2018.11.006  Raffaele, M., Licari, M., Amin, S., Alex, R., Shen, H., Singh, S. P., et al. (2020). Cold Press Pomegranate Seed Oil Attenuates Dietary-Obesity Induced Hepatic Steatosis and Fibrosis through Antioxidant and Mitochondrial Pathways in Obese Mice. *Int. J. Mol. Sci.* 21, 5469. doi: 10.3390/ijms21155469  Reguero, M., Reglero, G., Quintela, J. C., Ramos-Ruiz, R., Ramírez de Molina, A., and Gómez de Cedrón, M. (2024). Silymarin-Enriched Extract from Milk Thistle Activates Thermogenesis in a Preclinical Model of High-Fat-Diet-Induced Obesity to Relieve Systemic Meta-Inflammation. *Nutrients* 16, 4166. doi: 10.3390/nu16234166  Rocha, S., Amaro, A., Ferreira-Junior, M. D., Proença, C., Silva, A. M. S., Costa, V. M., et al. (2024). Melanoxetin: A Hydroxylated Flavonoid Attenuates Oxidative Stress and Modulates Insulin Resistance and Glycation Pathways in an Animal Model of Type 2 Diabetes Mellitus. *Pharmaceutics* 16, 261. doi: 10.3390/pharmaceutics16020261  Roy, J. R., Janaki, C. S., Jayaraman, S., Periyasamy, V., Balaji, T., Vijayamalathi, M., et al. (2022). Effect of Carica papaya on IRS-1/Akt Signaling Mechanisms in High-Fat-Diet–Streptozotocin-Induced Type 2 Diabetic Experimental Rats: A Mechanistic Approach. *Nutrients* 14, 4181. doi: 10.3390/nu14194181  Shamshoum, H., Vlavcheski, F., MacPherson, R. E. K., and Tsiani, E. (2021). Rosemary extract activates AMPK, inhibits mTOR and attenuates the high glucose and high insulin-induced muscle cell insulin resistance. *Appl. Physiol. Nutr. Metab.* 46, 819–827. doi: 10.1139/apnm-2020-0592  Su, H., Xie, L., Xu, Y., Ke, H., Bao, T., Li, Y., et al. (2020). Pelargonidin-3- O -glucoside Derived from Wild Raspberry Exerts Antihyperglycemic Effect by Inducing Autophagy and Modulating Gut Microbiota. *J. Agric. Food Chem.* 68, 13025–13037. doi: 10.1021/acs.jafc.9b03338  Surbala, L., Singh, C. B., Devi, R. V., and Singh, O. J. (2020). Rutaecarpine exhibits anti-diabetic potential in high fat diet–multiple low dose streptozotocin induced type 2 diabetic mice and in vitro by modulating hepatic glucose homeostasis. *J. Pharmacol. Sci.* 143, 307–314. doi: 10.1016/j.jphs.2020.04.008  Swargiary, D., Kashyap, B., Sarma, P., Ahmed, S. A., Gurumayum, S., Barge, S. R., et al. (2024). Free radical scavenging polyphenols isolated from Phyllanthus niruri L. ameliorates hyperglycemia via SIRT1 induction and GLUT4 translocation in in vitro and in vivo models. *Fitoterapia* 173, 105803. doi: 10.1016/j.fitote.2023.105803  Tahayneh, S., Qasem, B., Zakarneh, H., Shanak, S., and Zaid, H. (2024). Ligand-protein Docking of Gundelia tournefortii and Ocimum basilicum Derivatives in Scanning Hub Protein Targets (PI3K, PDK1, AKT, and RAC1) of the Insulin Signaling Pathway and ADME/Tox Drug Properties. *New Emirates Med. J.* 05. doi: 10.2174/0102506882306428240613104944  Takahashi, A., Ishizaki, M., Kimira, Y., Egashira, Y., and Hirai, S. (2021). Erucic Acid-Rich Yellow Mustard Oil Improves Insulin Resistance in KK-Ay Mice. *Molecules* 26, 546. doi: 10.3390/molecules26030546  Wahab, S., Khalid, M., Alqarni, M. H., Elagib, M. F. A., Bahamdan, G. K., Foudah, A. I., et al. (2023). Antihyperglycemic Potential of Spondias mangifera Fruits via Inhibition of 11β-HSD Type 1 Enzyme: In Silico and In Vivo Approach. *J. Clin. Med.* 12, 2152. doi: 10.3390/jcm12062152  Wang, Y., Zeng, T., Li, H., Wang, Y., Wang, J., and Yuan, H. (2023). Structural Characterization and Hypoglycemic Function of Polysaccharides from Cordyceps cicadae. *Molecules* 28, 526. doi: 10.3390/molecules28020526  Wu, F., Shao, Q., Xia, Q., Hu, M., Zhao, Y., Wang, D., et al. (2021). A bioinformatics and transcriptomics based investigation reveals an inhibitory role of Huanglian-Renshen-Decoction on hepatic glucose production of T2DM mice via PI3K/Akt/FoxO1 signaling pathway. *Phytomedicine* 83, 153487. doi: 10.1016/j.phymed.2021.153487  Wu, R., Jian, T., Ding, X., Lv, H., Meng, X., Ren, B., et al. (2021). Total Sesquiterpene Glycosides from Loquat Leaves Ameliorate HFD‐Induced Insulin Resistance by Modulating IRS‐1/GLUT4, TRPV1, and SIRT6/Nrf2 Signaling Pathways. *Oxid. Med. Cell. Longev.* 2021. doi: 10.1155/2021/4706410  Xu, J., Li, T., Xia, X., Fu, C., Wang, X., and Zhao, Y. (2020). Dietary Ginsenoside T19 Supplementation Regulates Glucose and Lipid Metabolism via AMPK and PI3K Pathways and Its Effect on Intestinal Microbiota. *J. Agric. Food Chem.* 68, 14452–14462. doi: 10.1021/acs.jafc.0c04429  Xu, W., Lu, Z., Wang, X., Cheung, M. H., Lin, M., Li, C., et al. (2020). Gynura divaricata exerts hypoglycemic effects by regulating the PI3K/AKT signaling pathway and fatty acid metabolism signaling pathway. *Nutr. Diabetes* 10, 31. doi: 10.1038/s41387-020-00134-z  Xu, X., Niu, L., Liu, Y., Pang, M., Lu, W., Xia, C., et al. (2020). Study on the mechanism of Gegen Qinlian Decoction for treating type II diabetes mellitus by integrating network pharmacology and pharmacological evaluation. *J. Ethnopharmacol.* 262, 113129. doi: 10.1016/j.jep.2020.113129  Yan, D., Fan, P., Sun, W., Ding, Q., Zheng, W., Xiao, W., et al. (2021). Anemarrhena asphodeloides modulates gut microbiota and restores pancreatic function in diabetic rats. *Biomed. Pharmacother.* 133, 110954. doi: 10.1016/j.biopha.2020.110954  Yan, S., Lu, W., Zhou, J., Guo, X., Li, J., Cheng, H., et al. (2022). Aqueous extract of Scrophularia ningpoensis improves insulin sensitivity through AMPK-mediated inhibition of the NLRP3 inflammasome. *Phytomedicine* 104, 154308. doi: 10.1016/j.phymed.2022.154308  Yang, R., Zhao, G., and Yan, B. (2022). Discovery of Novel c-Jun N-Terminal Kinase 1 Inhibitors from Natural Products: Integrating Artificial Intelligence with Structure-Based Virtual Screening and Biological Evaluation. *Molecules* 27, 6249. doi: 10.3390/molecules27196249  Yang, S., Zhao, M., Lu, M., Feng, Y., Zhang, X., Wang, D., et al. (2024). Network Pharmacology Analysis, Molecular Docking Integrated Experimental Verification Reveal the Mechanism of Gynostemma pentaphyllum in the Treatment of Type II Diabetes by Regulating the IRS1/PI3K/Akt Signaling Pathway. *Curr. Issues Mol. Biol.* 46, 5561–5581. doi: 10.3390/cimb46060333  Yao, Y., Chen, Y., Chen, H., Pan, X., Li, X., Liu, W., et al. (2024). Black mulberry extract inhibits hepatic adipogenesis through AMPK/mTOR signaling pathway in T2DM mice. *J. Ethnopharmacol.* 319, 117216. doi: 10.1016/j.jep.2023.117216  Ye, X., Chen, W., Huang, X.-F., Yan, F.-J., Deng, S.-G., Zheng, X.-D., et al. (2024). Anti-diabetic effect of anthocyanin cyanidin-3-O-glucoside: data from insulin resistant hepatocyte and diabetic mouse. *Nutr. Diabetes* 14, 7. doi: 10.1038/s41387-024-00265-7  Zhao, P., Zhong, S., Liao, J., Tao, J., Yao, Y., Song, P., et al. (2025). Caragana jubata ethanol extract ameliorates the symptoms of STZ-HFD-induced T2DM mice by PKC/GLUT4 pathway. *J. Ethnopharmacol.* 339, 119171. doi: 10.1016/j.jep.2024.119171  Zhu, J., Yu, C., Zhou, H., Wei, X., and Wang, Y. (2021). Comparative evaluation for phytochemical composition and regulation of blood glucose, hepatic oxidative stress and insulin resistance in mice and <scp>HepG2</scp> models of four typical <scp>Chinese</scp> dark teas. *J. Sci. Food Agric.* 101, 6563–6577. doi: 10.1002/jsfa.11328  Zima, K., Khaidakov, B., Banaszkiewicz, L., Lemke, K., and Kowalczyk, P. K. (2024). Exploring the Potential of Ribes nigrum L., Aronia melanocarpa (Michx.) Elliott, and Sambucus nigra L. Fruit Polyphenol‐Rich Composition and Metformin Synergy in Type 2 Diabetes Management. *J. Diabetes Res.* 2024. doi: 10.1155/2024/1092462  Zou, J., Song, Q., Shaw, P. C., Wu, Y., Zuo, Z., and Yu, R. (2024). Tangerine Peel-Derived Exosome-Like Nanovesicles Alleviate Hepatic Steatosis Induced by Type 2 Diabetes: Evidenced by Regulating Lipid Metabolism and Intestinal Microflora. *Int. J. Nanomedicine* Volume 19, 10023–10043. doi: 10.2147/IJN.S478589  Zuhri, U. M., Yuliana, N. D., Fadilah, F., Erlina, L., Purwaningsih, E. H., and Khatib, A. (2024). Exploration of the main active metabolites from Tinospora crispa (L.) Hook. f. &amp; Thomson stem as insulin sensitizer in L6.C11 skeletal muscle cell by integrating in vitro, metabolomics, and molecular docking. *J. Ethnopharmacol.* 319, 117296. doi: 10.1016/j.jep.2023.117296 |
| Insulin sensitization | Abdel-Rahman, R. F., Ezzat, S. M., Ogaly, H. A., Abd-Elsalam, R. M., Hessin, A. F., Fekry, M. I., et al. (2020). Ficus deltoidea extract down-regulates protein tyrosine phosphatase 1B expression in a rat model of type 2 diabetes mellitus: a new insight into its antidiabetic mechanism. *J. Nutr. Sci.* 9, e2. doi: 10.1017/jns.2019.40  Achyutha Devi, J., Bindu, G., and Ravi Kiran, S. (2023). Evaluation of in vitro glucose metabolism potential of sterols and essential oil extracts from Erythroxylum monogynum Roxb. using cell lines and enzyme inhibition models. *South African J. Bot.* 159, 110–130. doi: 10.1016/j.sajb.2023.06.008  Álvarez-Almazán, S., Solís-Domínguez, L. C., Duperou-Luna, P., Fuerte-Gómez, T., González-Andrade, M., Aranda-Barradas, M. E., et al. (2023). Anti-Diabetic Activity of Glycyrrhetinic Acid Derivatives FC-114 and FC-122: Scale-Up, In Silico, In Vitro, and In Vivo Studies. *Int. J. Mol. Sci.* 24, 12812. doi: 10.3390/ijms241612812  Bai, J., Zhang, S., Cao, J., Sun, H., Mang, Z., Shen, W. L., et al. (2022). Hernandezine, a natural herbal alkaloid, ameliorates type 2 diabetes by activating AMPK in two mouse models. *Phytomedicine* 105, 154366. doi: 10.1016/j.phymed.2022.154366  Barik, S. K., Dehury, B., Russell, W. R., Moar, K. M., Cruickshank, M., Scobbie, L., et al. (2020). Analysis of polyphenolic metabolites from in vitro gastrointestinal digested soft fruit extracts identify malvidin-3-glucoside as an inhibitor of PTP1B. *Biochem. Pharmacol.* 178, 114109. doi: 10.1016/j.bcp.2020.114109  Casertano, M., Genovese, M., Piazza, L., Balestri, F., Del Corso, A., Vito, A., et al. (2022). Identifying Human PTP1B Enzyme Inhibitors from Marine Natural Products: Perspectives for Developing of Novel Insulin-Mimetic Drugs. *Pharmaceuticals* 15, 325. doi: 10.3390/ph15030325  Casertano, M., Genovese, M., Santi, A., Pranzini, E., Balestri, F., Piazza, L., et al. (2023). Evidence of Insulin-Sensitizing and Mimetic Activity of the Sesquiterpene Quinone Avarone, a Protein Tyrosine Phosphatase 1B and Aldose Reductase Dual Targeting Agent from the Marine Sponge Dysidea avara. *Pharmaceutics* 15, 528. doi: 10.3390/pharmaceutics15020528  Chakraborty, K., and Dhara, S. (2021). Conoidecyclics A-C from marine macroalga Turbinaria conoides: Newly described natural macrolides with prospective bioactive properties. *Phytochemistry* 191, 112909. doi: 10.1016/j.phytochem.2021.112909  Fan, X., Jiao, G., Pang, T., Wen, T., He, Z., Han, J., et al. (2023). Ameliorative effects of mangiferin derivative TPX on insulin resistance via PI3K/AKT and AMPK signaling pathways in human HepG2 and HL-7702 hepatocytes. *Phytomedicine* 114, 154740. doi: 10.1016/j.phymed.2023.154740  Genovese, M., Imperatore, C., Casertano, M., Aiello, A., Balestri, F., Piazza, L., et al. (2021). Dual Targeting of PTP1B and Aldose Reductase with Marine Drug Phosphoeleganin: A Promising Strategy for Treatment of Type 2 Diabetes. *Mar. Drugs* 19, 535. doi: 10.3390/md19100535  Ha, M. T., Shrestha, S., Tran, T. H., Kim, J. A., Woo, M. H., Choi, J. S., et al. (2020). Inhibition of PTP1B by farnesylated 2-arylbenzofurans isolated from Morus alba root bark: unraveling the mechanism of inhibition based on in vitro and in silico studies. *Arch. Pharm. Res.* 43, 961–975. doi: 10.1007/s12272-020-01269-4  Hsing, H. Y., Rathnasamy, S., Dianita, R., and Wahab, H. A. (2020). Docking-based virtual screening in search for natural PTP1B inhibitors in treating type-2 diabetes mellitus and obesity. *Biomed. Res. Ther.* 7, 3579–3592. doi: 10.15419/bmrat.v7i1.585  Li, J., Zhang, Y., Yu, F., Pan, Y., Zhang, Z., He, Y., et al. (2023). Proteoglycan Extracted from Ganoderma lucidum Ameliorated Diabetes-Induced Muscle Atrophy via the AMPK/SIRT1 Pathway In Vivo and In Vitro. *ACS Omega* 8, 30359–30373. doi: 10.1021/acsomega.3c03513  Li, N., Li, X., Deng, M., Zhu, F., Wang, Z., Sheng, R., et al. (2023). Isosteviol derivatives as protein tyrosine Phosphatase-1B inhibitors: Synthesis, biological evaluation and molecular docking. *Bioorg. Med. Chem.* 83, 117240. doi: 10.1016/j.bmc.2023.117240  Liu, X., Zhang, Y., Chu, Y., Zhao, X., Mao, L., Zhao, S., et al. (2021). The natural compound rutaecarpine promotes white adipocyte browning through activation of the AMPK-PRDM16 axis. *Biochem. Biophys. Res. Commun.* 545, 189–194. doi: 10.1016/j.bbrc.2021.01.080  Macalalad, M. A. B., and Gonzales, A. A. (2023). In Silico Screening and Identification of Antidiabetic Inhibitors Sourced from Phytochemicals of Philippine Plants against Four Protein Targets of Diabetes (PTP1B, DPP-4, SGLT-2, and FBPase). *Molecules* 28, 5301. doi: 10.3390/molecules28145301  Meng, Q., Qi, X., Fu, Y., Chen, Q., Cheng, P., Yu, X., et al. (2020). Flavonoids extracted from mulberry (Morus alba L.) leaf improve skeletal muscle mitochondrial function by activating AMPK in type 2 diabetes. *J. Ethnopharmacol.* 248, 112326. doi: 10.1016/j.jep.2019.112326  Mugari, P., Nyoni, S., and Dzomba, P. (2024). Ethnomedicinal Plants, Associated Indigenous Knowledge and Phytochemical Composition of Extracts with Significant in vitro Antidiabetic Activity. *Pharmacognosy Res.* 16, 769–783. doi: 10.5530/pres.16.4.89  Nishikai-Shen, T., Hosono-Fukao, T., Ariga, T., Hosono, T., and Seki, T. (2022). Cinnamon extract improves abnormalities in glucose tolerance by decreasing Acyl-CoA synthetase long-chain family 1 expression in adipocytes. *Sci. Rep.* 12, 12574. doi: 10.1038/s41598-022-13421-9  Oliveira, K. A. de, Araújo, H. N., Lima, T. I. de, Oliveira, A. G., Favero-Santos, B. C., Guimarães, D. S. P. S. F., et al. (2021). Phytomodulatory proteins isolated from Calotropis procera latex promote glycemic control by improving hepatic mitochondrial function in HepG2 cells. *Saudi Pharm. J.* 29, 1061–1069. doi: 10.1016/j.jsps.2021.07.008  Rocha, S., Amaro, A., Ferreira-Junior, M. D., Proença, C., Silva, A. M. S., Costa, V. M., et al. (2024). Melanoxetin: A Hydroxylated Flavonoid Attenuates Oxidative Stress and Modulates Insulin Resistance and Glycation Pathways in an Animal Model of Type 2 Diabetes Mellitus. *Pharmaceutics* 16, 261. doi: 10.3390/pharmaceutics16020261  SarathKumar, B., and Lakshmi, B. S. (2019). In silico investigations on the binding efficacy and allosteric mechanism of six different natural product compounds towards PTP1B inhibition through docking and molecular dynamics simulations. *J. Mol. Model.* 25, 272. doi: 10.1007/s00894-019-4172-7  Sun, K., Ding, M., Fu, C., Li, P., Li, T., Fang, L., et al. (2023). Effects of dietary wild bitter melon (Momordica charantia var. abbreviate Ser.) extract on glucose and lipid metabolism in HFD/STZ-induced type 2 diabetic rats. *J. Ethnopharmacol.* 306, 116154. doi: 10.1016/j.jep.2023.116154  Wang, H., Huang, M., Bei, W., Yang, Y., Song, L., Zhang, D., et al. (2021). FTZ attenuates liver steatosis and fibrosis in the minipigs with type 2 diabetes by regulating the AMPK signaling pathway. *Biomed. Pharmacother.* 138, 111532. doi: 10.1016/j.biopha.2021.111532  Wang, X., Deng, Y., Wang, J., Qin, L., Du, Y., Zhang, Q., et al. (2024). New natural protein tyrosine phosphatase 1B inhibitors from Gynostemma pentaphyllum. *J. Enzyme Inhib. Med. Chem.* 39. doi: 10.1080/14756366.2024.2360063  Xu, J., Li, T., Xia, X., Fu, C., Wang, X., and Zhao, Y. (2020). Dietary Ginsenoside T19 Supplementation Regulates Glucose and Lipid Metabolism via AMPK and PI3K Pathways and Its Effect on Intestinal Microbiota. *J. Agric. Food Chem.* 68, 14452–14462. doi: 10.1021/acs.jafc.0c04429  Yan, S., Lu, W., Zhou, J., Guo, X., Li, J., Cheng, H., et al. (2022). Aqueous extract of Scrophularia ningpoensis improves insulin sensitivity through AMPK-mediated inhibition of the NLRP3 inflammasome. *Phytomedicine* 104, 154308. doi: 10.1016/j.phymed.2022.154308  Yoon, S.-Y., Ahn, D., Hwang, J. Y., Kang, M. J., and Chung, S. J. (2021). Linoleic acid exerts antidiabetic effects by inhibiting protein tyrosine phosphatases associated with insulin resistance. *J. Funct. Foods* 83, 104532. doi: 10.1016/j.jff.2021.104532  Yoon, S.-Y., Kim, J., Lee, B. S., Baek, S. C., Chung, S. J., and Kim, K. H. (2022). Terminalin from African Mango (Irvingia gabonensis) Stimulates Glucose Uptake through Inhibition of Protein Tyrosine Phosphatases. *Biomolecules* 12, 321. doi: 10.3390/biom12020321  Zhao, J.-F., Li, L.-H., Guo, X.-J., Zhang, H.-X., Tang, L.-L., Ding, C.-H., et al. (2023). Identification of natural product inhibitors of PTP1B based on high-throughput virtual screening strategy: In silico, in vitro and in vivo studies. *Int. J. Biol. Macromol.* 243, 125292. doi: 10.1016/j.ijbiomac.2023.125292  Zima, K., Khaidakov, B., Banaszkiewicz, L., Lemke, K., and Kowalczyk, P. K. (2024). Exploring the Potential of Ribes nigrum L., Aronia melanocarpa (Michx.) Elliott, and Sambucus nigra L. Fruit Polyphenol‐Rich Composition and Metformin Synergy in Type 2 Diabetes Management. *J. Diabetes Res.* 2024. doi: 10.1155/2024/1092462 |
| b-cell protection | Achyutha Devi, J., Bindu, G., and Ravi Kiran, S. (2023). Evaluation of in vitro glucose metabolism potential of sterols and essential oil extracts from Erythroxylum monogynum Roxb. using cell lines and enzyme inhibition models. *South African J. Bot.* 159, 110–130. doi: 10.1016/j.sajb.2023.06.008  Alghamdi, A. H., Shatla, I. M., Shreed, S., Khirelsied, A. H., and El-Refaei, M. F. (2023). Bee Honey Extract Attenuates Hyperglycemia in Induced Type 1 Diabetes: Impact of Antioxidant and Angiogenesis Activities on Diabetic Severity In Vivo. *Appl. Sci.* 13, 8045. doi: 10.3390/app13148045  Alzahrani, A. (2022). In silico study of four alkaloids as dipeptidyl peptidase-4 (DPP4) inhibitors to generate anti-diabetics effect. *Egypt. J. Chem.*, 0–0. doi: 10.21608/ejchem.2022.126394.5602  Ansari, P., Azam, S., Hannan, J. M. A., Flatt, P. R., and Abdel Wahab, Y. H. A. (2020). Anti-hyperglycaemic activity of H. rosa-sinensis leaves is partly mediated by inhibition of carbohydrate digestion and absorption, and enhancement of insulin secretion. *J. Ethnopharmacol.* 253, 112647. doi: 10.1016/j.jep.2020.112647  Ansari, P., Choudhury, S. T., and Abdel-Wahab, Y. H. A. (2022). Insulin Secretory Actions of Ethanol Extract of Eucalyptus citriodora Leaf, including Plasma DPP-IV and GLP-1 Levels in High-Fat-Fed Rats, as Well as Characterization of Biologically Effective Phytoconstituents. *Metabolites* 12, 757. doi: 10.3390/metabo12080757  Ansari, P., Flatt, P. R., Harriott, P., and Abdel-Wahab, Y. H. A. (2021). Anti-hyperglycaemic and insulin-releasing effects of Camellia sinensis leaves and isolation and characterisation of active compounds. *Br. J. Nutr.* 126, 1149–1163. doi: 10.1017/S0007114520005085  Ansari, P., Flatt, P. R., Harriott, P., Hannan, J. M. A., and Abdel-Wahab, Y. H. A. (2021). Identification of Multiple Pancreatic and Extra-Pancreatic Pathways Underlying the Glucose-Lowering Actions of Acacia arabica Bark in Type-2 Diabetes and Isolation of Active Phytoconstituents. *Plants* 10, 1190. doi: 10.3390/plants10061190  Ansari, P., Hannan, J. M. A., Choudhury, S. T., Islam, S. S., Talukder, A., Seidel, V., et al. (2022). Antidiabetic Actions of Ethanol Extract of Camellia sinensis Leaf Ameliorates Insulin Secretion, Inhibits the DPP-IV Enzyme, Improves Glucose Tolerance, and Increases Active GLP-1 (7–36) Levels in High-Fat-Diet-Fed Rats. *Medicines* 9, 56. doi: 10.3390/medicines9110056  Ansari, P., Hannon-Fletcher, M. P., Flatt, P. R., and Abdel-Wahab, Y. H. A. (2021). Effects of 22 traditional anti-diabetic medicinal plants on DPP-IV enzyme activity and glucose homeostasis in high-fat fed obese diabetic rats. *Biosci. Rep.* 41. doi: 10.1042/BSR20203824  Awote, O. K., Kanmodi, R. I., Ebube, S. C., and Abdulganniyyu, Z. F. (2024). Nutritional Profile, GC-MS Analysis and In-silico Anti-diabetic Phytocompounds Candidature of Jatropha gossypifolia Leaf Extracts. *Curr. Drug Discov. Technol.* 21. doi: 10.2174/0115701638267143230925172207  Awwad, A., Poucheret, P., Idres, Y. A., Tshibangu, D. S. T., Servent, A., Ferrare, K., et al. (2021). In Vitro Tests for a Rapid Evaluation of Antidiabetic Potential of Plant Species Containing Caffeic Acid Derivatives: A Validation by Two Well-Known Antidiabetic Plants, Ocimum gratissimum L. Leaf and Musanga cecropioides R. Br. ex Tedlie (Mu) Stem Bark. *Molecules* 26, 5566. doi: 10.3390/molecules26185566  Bahramsoltani, R., Farzaei, M. H., Sajadimajd, S., Iranpanah, A., Khazaei, M., Pourjabar, Z., et al. (2021). In vitro and in vivo antidiabetic activity of Tamarix stricta Boiss.: Role of autophagy. *J. Ethnopharmacol.* 269, 113692. doi: 10.1016/j.jep.2020.113692  Balogun, F. O., Naidoo, K., Aribisala, J. O., Pillay, C., and Sabiu, S. (2022). Cheminformatics Identification and Validation of Dipeptidyl Peptidase-IV Modulators from Shikimate Pathway-Derived Phenolic Acids towards Interventive Type-2 Diabetes Therapy. *Metabolites* 12, 937. doi: 10.3390/metabo12100937  Balogun, F. O., Singh, K., Rampadarath, A., Akoonjee, A., Naidoo, K., and Sabiu, S. (2023). Cheminformatics identification of modulators of key carbohydrate-metabolizing enzymes from C. cujete for type-2 diabetes mellitus intervention. *J. Diabetes Metab. Disord.* 22, 1299–1317. doi: 10.1007/s40200-023-01249-7  Chavan, A., Daniel, K., and Patel, A. M. (2022). In-silico Exploration of Phytoconstituents of Gymnema sylvestre as Potential Glucokinase Activators and DPP-IV Inhibitors for the Future Synthesis of Silver Nanoparticles for the Treatment of Type 2 Diabetes Mellitus. *Curr. Enzym. Inhib.* 18, 47–60. doi: 10.2174/1573408017666211029160203  Chen, Z., Su, X., Cao, W., Tan, M., Zhu, G., Gao, J., et al. (2024). The Discovery and Characterization of a Potent DPP-IV Inhibitory Peptide from Oysters for the Treatment of Type 2 Diabetes Based on Computational and Experimental Studies. *Mar. Drugs* 22, 361. doi: 10.3390/md22080361  dos Santos, F. A. R., Xavier, J. A., da Silva, F. C., Merlin, J. P. J., Goulart, M. O. F., and Rupasinghe, H. P. V. (2022). Antidiabetic, Antiglycation, and Antioxidant Activities of Ethanolic Seed Extract of Passiflora edulis and Piceatannol In Vitro. *Molecules* 27, 4064. doi: 10.3390/molecules27134064  El-Askary, H., Salem, H. H., and Abdel Motaal, A. (2022). Potential Mechanisms Involved in the Protective Effect of Dicaffeoylquinic Acids from Artemisia annua L. Leaves against Diabetes and Its Complications. *Molecules* 27, 857. doi: 10.3390/molecules27030857  Farid, A., Haridyy, H., Ashraf, S., Ahmed, S., and Safwat, G. (2022). Co-treatment with grape seed extract and mesenchymal stem cells in vivo regenerated beta cells of islets of Langerhans in pancreas of type I-induced diabetic rats. *Stem Cell Res. Ther.* 13, 528. doi: 10.1186/s13287-022-03218-y  FARKHANI, A., SAURIASARI, R., and YANUAR, A. (2020). IN SILICO APPROACH FOR SCREENING OF THE INDONESIAN MEDICINAL PLANTS DATABASE TO DISCOVER POTENTIAL DIPEPTIDYL PEPTIDASE-4 INHIBITORS. *Int. J. Appl. Pharm.*, 60–68. doi: 10.22159/ijap.2020.v12s1.FF008  Fawzi Mahomoodally, M., Picot-Allain, M. C. N., Zengin, G., Llorent-Martínez, E. J., Abdullah, H. H., Ak, G., et al. (2020). Phytochemical Analysis, Network Pharmacology and in Silico Investigations on Anacamptis pyramidalis Tuber Extracts. *Molecules* 25, 2422. doi: 10.3390/molecules25102422  García-Viñuales, S., Ilie, I. M., Santoro, A. M., Romanucci, V., Zarrelli, A., Di Fabio, G., et al. (2022). Silybins inhibit human IAPP amyloid growth and toxicity through stereospecific interactions. *Biochim. Biophys. Acta - Proteins Proteomics* 1870, 140772. doi: 10.1016/j.bbapap.2022.140772  Guo, F., Yao, L., Zhang, W., Chen, P., Hao, R., Huang, X., et al. (2023). The therapeutic mechanism of Yuye decoction on type 2 diabetes mellitus based on network pharmacology and experimental verification. *J. Ethnopharmacol.* 308, 116222. doi: 10.1016/j.jep.2023.116222  Han, M., Lu, Y., Tao, Y., Zhang, X., Dai, C., Zhang, B., et al. (2023). Luteolin Protects Pancreatic β Cells against Apoptosis through Regulation of Autophagy and ROS Clearance. *Pharmaceuticals* 16, 975. doi: 10.3390/ph16070975  Huang, T., Liu, P., Lin, Y., and Tsai, J. (2022). Hypoglycemic peptide‐enriched hydrolysates of <scp> *Corbicula fluminea* </scp> and <scp> *Chlorella sorokiniana* </scp> possess synergistic hypoglycemic activity through inhibiting α‐glucosidase and dipeptidyl peptidase‐4 activity. *J. Sci. Food Agric.* 102, 716–723. doi: 10.1002/jsfa.11402  Idoko, V. O., Sulaiman, M. A., Adamu, R. M., Abdullahi, A. D., Tajuddeen, N., Mohammed, A., et al. (2023). Evaluating Khaya senegalensis for Dipeptidyl Peptidase‐IV Inhibition Using in Vitro Analysis and Molecular Dynamic Simulation of Identified Bioactive Compounds. *Chem. Biodivers.* 20. doi: 10.1002/cbdv.202200909  Istrate, D., and Crisan, L. (2022). Natural Compounds as DPP-4 Inhibitors: 3D-Similarity Search, ADME Toxicity, and Molecular Docking Approaches. *Symmetry (Basel).* 14, 1842. doi: 10.3390/sym14091842  Jasim, A. R. M., Abhirami, B. L., Anto, E. M., George, S., Jayamurthy, P., and Kumaran, A. (2024). Multimodal therapeutic amelioration of type 2 diabetes via bioactive compounds isolated from Cassia mimosoides L. *South African J. Bot.* 172, 567–578. doi: 10.1016/j.sajb.2024.07.060  Ji, S., Zhu, C., Gao, S., Shao, X., Chen, X., Zhang, H., et al. (2021). Morus alba leaves ethanol extract protects pancreatic islet cells against dysfunction and death by inducing autophagy in type 2 diabetes. *Phytomedicine* 83, 153478. doi: 10.1016/j.phymed.2021.153478  Kalhotra, P., Chittepu, V. C. S. R., Osorio-Revilla, G., and Gallardo-Velazquez, T. (2020). Phytochemicals in Garlic Extract Inhibit Therapeutic Enzyme DPP-4 and Induce Skeletal Muscle Cell Proliferation: A Possible Mechanism of Action to Benefit the Treatment of Diabetes Mellitus. *Biomolecules* 10, 305. doi: 10.3390/biom10020305  Kumar, P., Ram, H., Kala, C., Kashyap, P., Singh, G., Agnihotri, C., et al. (2023). DPP-4 inhibition mediated antidiabetic potential of phytoconstituents of an aqueous fruit extract of Withania coagulans (Stocks) Dunal: in-silico , in-vitro and in-vivo assessments. *J. Biomol. Struct. Dyn.* 41, 6145–6167. doi: 10.1080/07391102.2022.2103029  Kumar, S., Niguram, P., Bhat, V., Jinagal, S., Jairaj, V., and Chauhan, N. (2022). Synthesis, molecular docking and ADMET prediction of novel swertiamarin analogues for the restoration of type-2 diabetes: an enzyme inhibition assay. *Nat. Prod. Res.* 36, 2197–2207. doi: 10.1080/14786419.2020.1825428  Kumar, V., Sachan, R., Rahman, M., Sharma, K., Al-Abbasi, F. A., and Anwar, F. (2021). Prunus amygdalus extract exert antidiabetic effect via inhibition of DPP-IV: in-silico and in-vivo approaches. *J. Biomol. Struct. Dyn.* 39, 4160–4174. doi: 10.1080/07391102.2020.1775124  Liao, H.-J., and Tzen, J. T. C. (2022). The Potential Role of Cyclopeptides from Pseudostellaria heterophylla, Linum usitatissimum and Drymaria diandra, and Peptides Derived from Heterophyllin B as Dipeptidyl Peptidase IV Inhibitors for the Treatment of Type 2 Diabetes: An In Silico Study. *Metabolites* 12, 387. doi: 10.3390/metabo12050387  Liao, H.-J., and Tzen, J. T. C. (2022). Investigating Potential GLP-1 Receptor Agonists in Cyclopeptides from Pseudostellaria heterophylla, Linum usitatissimum, and Drymaria diandra, and Peptides Derived from Heterophyllin B for the Treatment of Type 2 Diabetes: An In Silico Study. *Metabolites* 12, 549. doi: 10.3390/metabo12060549  Lin, L.-C., Lee, L.-C., Huang, C., Chen, C.-T., Song, J.-S., Shiao, Y.-J., et al. (2019). Effects of boschnaloside from Boschniakia rossica on dysglycemia and islet dysfunction in severely diabetic mice through modulating the action of glucagon-like peptide-1. *Phytomedicine* 62, 152946. doi: 10.1016/j.phymed.2019.152946  Liu, L., Liang, C., Mei, P., Zhu, H., Hou, M., Yu, C., et al. (2019). Dracorhodin perchlorate protects pancreatic β‐cells against glucotoxicity‐ or lipotoxicity‐induced dysfunction and apoptosis in vitro and in vivo. *FEBS J.* 286, 3718–3736. doi: 10.1111/febs.15020  Lüersen, K., Fischer, A., Bauer, I., Huebbe, P., Uekaji, Y., Chikamoto, K., et al. (2023). Soy Extract, Rich in Hydroxylated Isoflavones, Exhibits Antidiabetic Properties In Vitro and in Drosophila melanogaster In Vivo. *Nutrients* 15, 1392. doi: 10.3390/nu15061392  Lundqvist, L. C. E., Rattigan, D., Ehtesham, E., Demmou, C., Östenson, C.-G., and Sandström, C. (2019). Profiling and activity screening of Dammarane-type triterpen saponins from Gynostemma pentaphyllum with glucose-dependent insulin secretory activity. *Sci. Rep.* 9, 627. doi: 10.1038/s41598-018-37517-3  Manzano, J. A. H., Llames, L. C. J., and Macabeo, A. P. G. (2023). Tetrahydrobisbenzylisoquinoline alkaloids from Phaeanthus ophthalmicus inhibit target enzymes associated with type 2 diabetes and obesity. *J. Appl. Pharm. Sci.* doi: 10.7324/JAPS.2023.154518  Martini, S., Solieri, L., Cattivelli, A., Pizzamiglio, V., and Tagliazucchi, D. (2021). An Integrated Peptidomics and In Silico Approach to Identify Novel Anti-Diabetic Peptides in Parmigiano-Reggiano Cheese. *Biology (Basel).* 10, 563. doi: 10.3390/biology10060563  Matsabisa, M. G., Chukwuma, C. I., Chaudhary, S. K., Kumar, C. S., Baleni, R., Javu, M., et al. (2020). Dicoma anomala (Sond.) abates glycation and DPP-IV activity and modulates glucose utilization in Chang liver cells and 3T3-L1 adipocytes. *South African J. Bot.* 128, 182–188. doi: 10.1016/j.sajb.2019.09.013  Mkabayi, L., Viljoen, Z., Krause, R. W. M., Lobb, K. A., Pletschke, B. I., and Frost, C. L. (2024). Inhibitory effects of selected cannabinoids against dipeptidyl peptidase IV, an enzyme linked to type 2 diabetes. *Heliyon* 10, e23289. doi: 10.1016/j.heliyon.2023.e23289  Moens, C., Muller, C. J. F., and Bouwens, L. (2022). In vitro comparison of various antioxidants and flavonoids from Rooibos as beta cell protectants against lipotoxicity and oxidative stress-induced cell death. *PLoS One* 17, e0268551. doi: 10.1371/journal.pone.0268551  Mohanty, I., Kumar, Cs., and Borde, M. (2021). Antidiabetic activity of Commiphora mukul and Phyllanthus emblica and Computational analysis for the identification of active principles with dipeptidyl peptidase IV inhibitory activity. *Indian J. Pharmacol.* 53, 384. doi: 10.4103/ijp.IJP_69_19  Mugari, P., Nyoni, S., and Dzomba, P. (2024). Ethnomedicinal Plants, Associated Indigenous Knowledge and Phytochemical Composition of Extracts with Significant in vitro Antidiabetic Activity. *Pharmacognosy Res.* 16, 769–783. doi: 10.5530/pres.16.4.89  Nabi, F., Ahmad, O., Khan, A., Hassan, M. N., Hisamuddin, M., Malik, S., et al. (2024). Natural compound plumbagin based inhibition of <scp>hIAPP</scp> revealed by Markov state models based on <scp>MD</scp> data along with experimental validations. *Proteins Struct. Funct. Bioinforma.* 92, 1070–1084. doi: 10.1002/prot.26682  Nagy, L., Béke, F., Juhász, L., Kovács, T., Juhász-Tóth, É., Docsa, T., et al. (2020). Glycogen phosphorylase inhibitor, 2,3‐bis[(2E)‐3‐(4‐hydroxyphenyl)prop‐2‐enamido] butanedioic acid (BF142), improves baseline insulin secretion of MIN6 insulinoma cells. *PLoS One* 15, e0236081. doi: 10.1371/journal.pone.0236081  Nandeshwar, Rout, J., Panda, S. M., and Tripathy, U. (2024). Phytoconstituents of Ashwagandha as potential inhibitors of human islet amyloid polypeptide (hIAPP): an in silico investigation. *J. Biomol. Struct. Dyn.* 42, 11020–11036. doi: 10.1080/07391102.2023.2259491  Ovalle-Magallanes, B., Navarrete, A., Haddad, P. S., Tovar, A. R., Noriega, L. G., Tovar-Palacio, C., et al. (2019). Multi-target antidiabetic mechanisms of mexicanolides from Swietenia humilis. *Phytomedicine* 58, 152891. doi: 10.1016/j.phymed.2019.152891  Oyebode, O., Zuma, L., Lucky Erukainure, O., Koorbanally, N., and Islam, M. S. (2023). Bridelia ferruginea inhibits key carbohydrate digesting enzyme and intestinal glucose absorption and modulates glucose metabolism in diabetic rats. *Arch. Physiol. Biochem.* 129, 671–681. doi: 10.1080/13813455.2020.1861026  Oyedemi, S. O., Atanes, P., Aiyegoro, O. A., Amoo, S. O., Swain, S. S., and Persaud, S. J. (2023). In vitro profiling and functional assessments of the anti‐diabetic capacity of phenolic‐rich extracts of Bulbine natalensis and Bulbine frutescens. *Diabet. Med.* 40. doi: 10.1111/dme.14770  Parveen, S., Shehzadi, S., Shafiq, N., Rashid, M., Naz, S., Mehmood, T., et al. (2025). A discovery of potent kaempferol derivatives as multi-target medicines against diabetes as well as bacterial infections: an in silico approach. *J. Biomol. Struct. Dyn.* 43, 5218–5240. doi: 10.1080/07391102.2024.2308773  Patle, D., Kaur, P., Khurana, N., and Sahu, S. K. (2024). Synthesis, Molecular Docking Analysis and In vitro Evaluation of Potential Anti-Diabetic Candidates with Harnessing the Effectiveness of Scoparia Dulcis Plant. *Chem. Africa* 7, 3093–3107. doi: 10.1007/s42250-024-01006-0  Patle, D., Khurana, N., Gupta, J., Kaur, P., and Khatik, G. L. (2023). Design, synthesis, and biological evaluation of coixol-based derivatives as potential antidiabetic agents. *J. Mol. Struct.* 1277, 134861. doi: 10.1016/j.molstruc.2022.134861  Perumal, N., Nallappan, M., Shohaimi, S., Kassim, N. K., Tee, T. T., and Cheah, Y. H. (2022). Synergistic antidiabetic activity of Taraxacum officinale (L.) Weber ex F.H.Wigg and Momordica charantia L. polyherbal combination. *Biomed. Pharmacother.* 145, 112401. doi: 10.1016/j.biopha.2021.112401  Purnomo, Y., W Soeatmadji, D., B Sumitro, S., and Widodo, M. A. (2023). Dipeptidyl Peptidase-4 Inhibitory Activity of Indonesian Anti-Diabetic Herbs: Carica papaya, Tithonia diversifolia, Urena lobata. *Res. J. Pharm. Technol.*, 273–277. doi: 10.52711/0974-360X.2023.00050  Quimque, M. T. J., Magsipoc, R. J. Y., Llames, L. C. J., Flores, A. I. G., Garcia, K. Y. M., Ratzenböck, A., et al. (2022). Polyoxygenated Cyclohexenes from Uvaria grandiflora with Multi-Enzyme Targeting Properties Relevant in Type 2 Diabetes and Obesity. *ACS Omega* 7, 36856–36864. doi: 10.1021/acsomega.2c05544  Quintero‐Soto, M. F., Chávez‐Ontiveros, J., Garzón‐Tiznado, J. A., Salazar‐Salas, N. Y., Pineda‐Hidalgo, K. V., Delgado‐Vargas, F., et al. (2021). Characterization of peptides with antioxidant activity and antidiabetic potential obtained from chickpea ( Cicer arietinum L.) protein hydrolyzates. *J. Food Sci.* 86, 2962–2977. doi: 10.1111/1750-3841.15778  Sajid, M., Khan, M. R., Ismail, H., Latif, S., Rahim, A. A., Mehboob, R., et al. (2020). Antidiabetic and antioxidant potential of Alnus nitida leaves in alloxan induced diabetic rats. *J. Ethnopharmacol.* 251, 112544. doi: 10.1016/j.jep.2020.112544  Sharma, P., Joshi, T., Mathpal, S., Chandra, S., and Tamta, S. (2022). In silico identification of antidiabetic target for phytochemicals of A. marmelos and mechanistic insights by molecular dynamics simulations. *J. Biomol. Struct. Dyn.* 40, 10543–10560. doi: 10.1080/07391102.2021.1944910  Tan, S. C., Rajendran, R., Bhattamisra, S. K., Krishnappa, P., Davamani, F., Chitra, E., et al. (2025). Protective effects of madecassoside, a triterpenoid from Centella asiatica , against oxidative stress in INS-1E cells. *Nat. Prod. Res.* 39, 2787–2794. doi: 10.1080/14786419.2024.2315499  Vijh, D., and Gupta, P. (2024). GC–MS analysis, molecular docking, and pharmacokinetic studies on Dalbergia sissoo barks extracts for compounds with anti-diabetic potential. *Sci. Rep.* 14, 24936. doi: 10.1038/s41598-024-75570-3  Wang, S., Sheng, H., Bai, Y., Weng, Y., Fan, X., Zheng, F., et al. (2021). Inhibition of histone acetyltransferase by naringenin and hesperetin suppresses Txnip expression and protects pancreatic β cells in diabetic mice. *Phytomedicine* 88, 153454. doi: 10.1016/j.phymed.2020.153454  Widyawati, T., Syahputra, R. A., Syarifah, S., and Sumantri, I. B. (2023). Analysis of Antidiabetic Activity of Squalene via In Silico and In Vivo Assay. *Molecules* 28, 3783. doi: 10.3390/molecules28093783  XING, Q.-C., LIU, X., LI, W., CHEN, Y.-Z., and CHEN, J. (2020). Sangguayin preparation prevents palmitate-induced apoptosis by suppressing endoplasmic reticulum stress and autophagy in db/db mice and MIN6 pancreatic β-cells. *Chin. J. Nat. Med.* 18, 472–480. doi: 10.1016/S1875-5364(20)30054-6  Xu, J., Fu, C., Li, T., Xia, X., Zhang, H., Wang, X., et al. (2021). Protective effect of acorn (Quercus liaotungensis Koidz) on streptozotocin-damaged MIN6 cells and type 2 diabetic rats via p38 MAPK/Nrf2/HO-1 pathway. *J. Ethnopharmacol.* 266, 113444. doi: 10.1016/j.jep.2020.113444  Xu, L., Jois, S., and Cui, H. (2022). Metformin and Gegen Qinlian Decoction boost islet α-cell proliferation of the STZ induced diabetic rats. *BMC Complement. Med. Ther.* 22, 193. doi: 10.1186/s12906-022-03674-2  Yan, D., Fan, P., Sun, W., Ding, Q., Zheng, W., Xiao, W., et al. (2021). Anemarrhena asphodeloides modulates gut microbiota and restores pancreatic function in diabetic rats. *Biomed. Pharmacother.* 133, 110954. doi: 10.1016/j.biopha.2020.110954  Zabidi, N. A., Ishak, N. A., Hamid, M., Ashari, S. E., and Mohammad Latif, M. A. (2021). Inhibitory evaluation of Curculigo latifolia on α-glucosidase, DPP (IV) and in vitro studies in antidiabetic with molecular docking relevance to type 2 diabetes mellitus. *J. Enzyme Inhib. Med. Chem.* 36, 109–121. doi: 10.1080/14756366.2020.1844680  Zambrana, S., Mamani, O., Canaviri, M., Gutierrez, M., Catrina, S., Ostenson, C., et al. (2021). Glycemia-reducing effects of Bolivian nutraceutical plants. *Ars Pharm* 62, 52–65. doi: 10.30827/ars.v62i1.15456  Zhang, X., Sun, Z., Sun, W., Li, Y., Gao, F., Teng, F., et al. (2024). Bioinformatics Analysis and Experimental Findings Reveal the Therapeutic Actions and Targets of Cyathulae Radix Against Type 2 Diabetes Mellitus. *J. Diabetes Res.* 2024. doi: 10.1155/2024/5521114  Zhang, Y., Zhou, G., Peng, Y., Wang, M., and Li, X. (2020). Anti-hyperglycemic and anti-hyperlipidemic effects of a special fraction of Luohanguo extract on obese T2DM rats. *J. Ethnopharmacol.* 247, 112273. doi: 10.1016/j.jep.2019.112273  Zhao, C., Zhao, H., Zhang, C.-C., Yang, X.-H., Chen, K., Xue, Y., et al. (2023). Impact of Lycium barbarum polysaccharide on the expression of glucagon-like peptide 1 in vitro and in vivo. *Int. J. Biol. Macromol.* 224, 908–918. doi: 10.1016/j.ijbiomac.2022.10.176  Zheng, H., Zhao, L., Xie, Y., and Tan, Y. (2024). Purification and Identification of Novel Dipeptidyl Peptidase IV Inhibitory Peptides Derived from Bighead Carp (Hypophthalmichthys nobilis). *Foods* 13, 2644. doi: 10.3390/foods13172644 |
| Lipid metabolism improvement | Bharadwaja, S., Issac, P. K., Cleta, J., Jeganathan, R., Chandrakumar, S. S., and Sundaresan, S. (2021). An in vitro mechanistic approach towards understanding the distinct pathways regulating insulin resistance and adipogenesis by apocynin. *J. Biosci.* 46. Available at: http://www.ncbi.nlm.nih.gov/pubmed/33709960  Campos, M. L. de, Castro, M. B. de, Campos, A. D., Fernandes, M. F., Conegundes, J. L. M., Rodrigues, M. N., et al. (2021). Antiobesity, hepatoprotective and anti-hyperglycemic effects of a pharmaceutical formulation containing Cecropia pachystachya Trécul in mice fed with a hypercaloric diet. *J. Ethnopharmacol.* 280, 114418. doi: 10.1016/j.jep.2021.114418  Cardullo, N., Calcagno, D., Pulvirenti, L., Sciacca, C., Pittalà, M. G. G., Maccarronello, A. E., et al. (2024). Flavonoids with lipase inhibitory activity from lemon squeezing waste: isolation, multispectroscopic and <scp> *in silico* </scp> studies. *J. Sci. Food Agric.* 104, 7639–7648. doi: 10.1002/jsfa.13600  Choi, E. M., Suh, K. S., Park, S. Y., Yun, S., Chin, S. O., Rhee, S. Y., et al. (2020). Orientin reduces the inhibitory effects of 2,3,7,8-tetrachlorodibenzo-p-dioxin on adipogenic differentiation and insulin signaling pathway in murine 3T3-L1 adipocytes. *Chem. Biol. Interact.* 318, 108978. doi: 10.1016/j.cbi.2020.108978  De la Fuente-Muñoz, M., De la Fuente-Fernández, M., Román-Carmena, M., Amor, S., Iglesias-de la Cruz, M. C., García-Laínez, G., et al. (2023). Supplementation with a New Standardized Extract of Green and Black Tea Exerts Antiadipogenic Effects and Prevents Insulin Resistance in Mice with Metabolic Syndrome. *Int. J. Mol. Sci.* 24, 8521. doi: 10.3390/ijms24108521  Dound, Y. A., Chaudhary, S., Chaudhary, S. S., Rawat, S., Alqarni, M. H., Ahmad, M. M., et al. (2021). Mechanistic understanding of PtyroneTM: A plant based natural anti diabetic product. *J. King Saud Univ. - Sci.* 33, 101454. doi: 10.1016/j.jksus.2021.101454  Elrherabi, A., Abdnim, R., Loukili, E. H., Laftouhi, A., Lafdil, F. Z., Bouhrim, M., et al. (2024). Antidiabetic potential of Lavandula stoechas aqueous extract: insights into pancreatic lipase inhibition, antioxidant activity, antiglycation at multiple stages and anti-inflammatory effects. *Front. Pharmacol.* 15. doi: 10.3389/fphar.2024.1443311  Fawzi Mahomoodally, M., Picot-Allain, M. C. N., Zengin, G., Llorent-Martínez, E. J., Abdullah, H. H., Ak, G., et al. (2020). Phytochemical Analysis, Network Pharmacology and in Silico Investigations on Anacamptis pyramidalis Tuber Extracts. *Molecules* 25, 2422. doi: 10.3390/molecules25102422  Giacoman-Martínez, A., Alarcón-Aguilar, F., Zamilpa, A., Hidalgo-Figueroa, S., Navarrete-Vázquez, G., García-Macedo, R., et al. (2019). Triterpenoids from Hibiscus sabdariffa L. with PPARδ/γ Dual Agonist Action: In Vivo, In Vitro and In Silico Studies. *Planta Med.* 85, 412–423. doi: 10.1055/a-0824-1316  Giles-Rivas, D., Estrada-Soto, S., Aguilar-Guadarrama, A. B., Almanza-Pérez, J., García-Jiménez, S., Colín-Lozano, B., et al. (2020). Antidiabetic effect of Cordia morelosana, chemical and pharmacological studies. *J. Ethnopharmacol.* 251, 112543. doi: 10.1016/j.jep.2020.112543  Gulisano, M., Consoli, V., Sorrenti, V., and Vanella, L. (2024). Red Oranges and Olive Leaf Waste-Derived Bioactive Extracts Promote Adipocyte Functionality In Vitro. *Nutrients* 16, 1959. doi: 10.3390/nu16121959  Heo, S.-W., Chung, K.-S., Yoon, Y.-S., Kim, S.-Y., Ahn, H.-S., Shin, Y.-K., et al. (2023). Standardized Ethanol Extract of Cassia mimosoides var. nomame Makino Ameliorates Obesity via Regulation of Adipogenesis and Lipogenesis in 3T3-L1 Cells and High-Fat Diet-Induced Obese Mice. *Nutrients* 15, 613. doi: 10.3390/nu15030613  Hwang, M.-S., Baek, J.-H., Song, J.-K., Lee, I. H., and Chun, K.-H. (2023). Tschimganidine reduces lipid accumulation through AMPK activation and alleviates high-fat diet-induced metabolic diseases. *BMB Rep.* 56, 246–251. doi: 10.5483/BMBRep.2022-0211  Islas-Garduño, A. L., Romero-Cerecero, O., Jiménez-Aparicio, A. R., Tortoriello, J., Montiel-Ruiz, R. M., González-Cortazar, M., et al. (2023). Pharmacological and Chemical Analysis of Bauhinia divaricata L. Using an In Vitro Antiadipogenic Model. *Plants* 12, 3799. doi: 10.3390/plants12223799  Jyothi Reddy, G., Bhaskar Reddy, K., and Subba Reddy, G. V. (2020). In vivo Anti-diabetic and Anti-hyperlipidemic Activities of ethyl Acetate/Methanol Fractions of Feronia elephantum Fruit in type 2 Diabetic Rats: Via α-amylase and PPAR-γ by using in silico Approach. *Indian J. Pharm. Educ. Res.* 54, 761–770. doi: 10.5530/ijper.54.3.128  Kiage-Mokua, B. N., De Vrese, M., Kraus-Stojanowic, I., Nielsen, A., Kareru, P., Kenji, G., et al. (2020). Effect of extracts from selected Kenyan plants on traits of metabolic syndrom in Wistar rats fed a high-fat high fructose diet. *Trop. J. Pharm. Res.* 19, 2137–2146. doi: 10.4314/tjpr.v19i10.18  Kuranov, S. O., Luzina, O. A., Onopchenko, O., Pishel, I., Zozulya, S., Gureev, M., et al. (2020). Exploring bulky natural and natural-like periphery in the design of p-(benzyloxy)phenylpropionic acid agonists of free fatty acid receptor 1 (GPR40). *Bioorg. Chem.* 99, 103830. doi: 10.1016/j.bioorg.2020.103830  Le, D. D., Kim, E., Dang, T., Lee, J., Shin, C. H., Park, J. W., et al. (2024). Chemical Investigation and Regulation of Adipogenic Differentiation of Cultivated Moringa oleifera. *Pharmaceuticals* 17, 1310. doi: 10.3390/ph17101310  Lee, J.-Y., Kim, T. Y., Kang, H., Oh, J., Park, J. W., Kim, S.-C., et al. (2021). Anti-Obesity and Anti-Adipogenic Effects of Chitosan Oligosaccharide (GO2KA1) in SD Rats and in 3T3-L1 Preadipocytes Models. *Molecules* 26, 331. doi: 10.3390/molecules26020331  Lian, Y.-E., Wang, M., Ma, L., Yi, W., Liao, S., Gao, H., et al. (2024). Identification of Novel PPARγ Partial Agonists Based on Virtual Screening Strategy: In Silico and In Vitro Experimental Validation. *Molecules* 29, 4881. doi: 10.3390/molecules29204881  Liu, E., Tsuboi, H., Ikegami, S., Kamiyama, T., Asami, Y., Ye, L., et al. (2021). Effects of Nelumbo nucifera Leaf Extract on Obesity. *Plant Foods Hum. Nutr.* 76, 377–384. doi: 10.1007/s11130-020-00852-w  Liu, W., Chen, X., Ge, Y., Wang, H., Phosat, C., Li, J., et al. (2020). Network pharmacology strategy for revealing the pharmacological mechanism of pharmacokinetic target components of San-Ye-Tang-Zhi-Qing formula for the treatment of type 2 diabetes mellitus. *J. Ethnopharmacol.* 260, 113044. doi: 10.1016/j.jep.2020.113044  Loza-Rodríguez, H., Estrada-Soto, S., Alarcón-Aguilar, F. J., Huang, F., Aquino-Jarquín, G., Fortis-Barrera, Á., et al. (2020). Oleanolic acid induces a dual agonist action on PPARγ/α and GLUT4 translocation: A pentacyclic triterpene for dyslipidemia and type 2 diabetes. *Eur. J. Pharmacol.* 883, 173252. doi: 10.1016/j.ejphar.2020.173252  Lüersen, K., Fischer, A., Bauer, I., Huebbe, P., Uekaji, Y., Chikamoto, K., et al. (2023). Soy Extract, Rich in Hydroxylated Isoflavones, Exhibits Antidiabetic Properties In Vitro and in Drosophila melanogaster In Vivo. *Nutrients* 15, 1392. doi: 10.3390/nu15061392  Luo, Z., Liu, Y., Han, X., Yang, W., Wang, G., Wang, J., et al. (2021). Mechanism of Paeoniae Radix Alba in the Treatment of Non-alcoholic Fatty Liver Disease Based on Sequential Metabolites Identification Approach, Network Pharmacology, and Binding Affinity Measurement. *Front. Nutr.* 8. doi: 10.3389/fnut.2021.677659  Ma, Y., Du, X., Zhao, D., Tang, K., Wang, X., Guo, S., et al. (2021). 18:0 Lyso PC, a natural product with potential PPAR-γ agonistic activity, plays hypoglycemic effect with lower liver toxicity and cardiotoxicity in db/db mice. *Biochem. Biophys. Res. Commun.* 579, 168–174. doi: 10.1016/j.bbrc.2021.09.059  Mesquita, P. G., Araujo, L. M. de, Neves, F. de A. R., and Borin, M. de F. (2022). Metabolites of endophytic fungi isolated from leaves of Bauhinia variegata exhibit antioxidant activity and agonist activity on peroxisome proliferator-activated receptors α, β/δ and γ. *Front. Fungal Biol.* 3. doi: 10.3389/ffunb.2022.1049690  Nakayama, H., Hata, K., Matsuoka, I., Zang, L., Kim, Y., Chu, D., et al. (2020). Anti-Obesity Natural Products Tested in Juvenile Zebrafish Obesogenic Tests and Mouse 3T3-L1 Adipogenesis Assays. *Molecules* 25, 5840. doi: 10.3390/molecules25245840  Nasreen, W., Sarker, S., Sufian, M. A., Md. Opo, F. A. D., Shahriar, M., Akhter, R., et al. (2020). A possible alternative therapy for type 2 diabetes using Myristica fragrans Houtt in combination with glimepiride: in vivo evaluation and in silico support. *Zeitschrift für Naturforsch. C* 75, 103–112. doi: 10.1515/znc-2019-0134  Noruddin, N. A. A., Hamzah, M. F., Rosman, Z., Salin, N. H., Shu-Chien, A. C., and Muhammad, T. S. T. (2021). Natural Compound 3β,7β,25-trihydroxycucurbita-5,23(E)-dien-19-al from Momordica charantia Acts as PPARγ Ligand. *Molecules* 26, 2682. doi: 10.3390/molecules26092682  Nur Kabidul Azam, M., Biswas, P., Mohaimenul Islam Tareq, M., Ridoy Hossain, M., Bibi, S., Anisul Hoque, M., et al. (2024). Identification of antidiabetic inhibitors from Allophylus villosus and Mycetia sinensis by targeting α-glucosidase and PPAR-γ: In-vitro, in-vivo, and computational evidence. *Saudi Pharm. J.* 32, 101884. doi: 10.1016/j.jsps.2023.101884  Pinto, C., Ibáñez, M. R., Loyola, G., León, L., Salvatore, Y., González, C., et al. (2021). Characterization of an Agarophyton chilense Oleoresin Containing PPARγ Natural Ligands with Insulin-Sensitizing Effects in a C57Bl/6J Mouse Model of Diet-Induced Obesity and Antioxidant Activity in Caenorhabditis elegans. *Nutrients* 13, 1828. doi: 10.3390/nu13061828  Prabhu, D. S., and Rajeswari, V. D. (2020). PPAR-Gamma as putative gene target involved in Butein mediated anti-diabetic effect. *Mol. Biol. Rep.* 47, 5273–5283. doi: 10.1007/s11033-020-05605-1  Pucci, M., Mandrone, M., Chiocchio, I., Sweeney, E. Mac, Tirelli, E., Uberti, D., et al. (2022). Different Seasonal Collections of Ficus carica L. Leaves Diversely Modulate Lipid Metabolism and Adipogenesis in 3T3-L1 Adipocytes. *Nutrients* 14, 2833. doi: 10.3390/nu14142833  Reguero, M., Reglero, G., Quintela, J. C., Ramos-Ruiz, R., Ramírez de Molina, A., and Gómez de Cedrón, M. (2024). Silymarin-Enriched Extract from Milk Thistle Activates Thermogenesis in a Preclinical Model of High-Fat-Diet-Induced Obesity to Relieve Systemic Meta-Inflammation. *Nutrients* 16, 4166. doi: 10.3390/nu16234166  Sahin, C., Melanson, J.-R., Le Billan, F., Magomedova, L., Ferreira, T. A. M., Oliveira, A. S., et al. (2024). A novel fatty acid mimetic with pan-PPAR partial agonist activity inhibits diet-induced obesity and metabolic dysfunction-associated steatotic liver disease. *Mol. Metab.* 85, 101958. doi: 10.1016/j.molmet.2024.101958  Song, J.-H., Kim, H., Jeong, M., Kong, M. J., Choi, H.-K., Jun, W., et al. (2021). In Vivo Evaluation of Dendropanax morbifera Leaf Extract for Anti-Obesity and Cholesterol-Lowering Activity in Mice. *Nutrients* 13, 1424. doi: 10.3390/nu13051424  Sut, S., Tahmasebi, A., Ferri, N., Ferrarese, I., Rossi, I., Panighel, G., et al. (2022). NMR, LC-MS Characterization of Rydingia michauxii Extracts, Identification of Natural Products Acting as Modulators of LDLR and PCSK9. *Molecules* 27, 2256. doi: 10.3390/molecules27072256  Takahashi, A., Ishizaki, M., Kimira, Y., Egashira, Y., and Hirai, S. (2021). Erucic Acid-Rich Yellow Mustard Oil Improves Insulin Resistance in KK-Ay Mice. *Molecules* 26, 546. doi: 10.3390/molecules26030546  Tomar, R., Mishra, S. S., Sahoo, J., and Rath, S. K. (2024). Computational and in-vitro Investigation of Phytochemicals from Allamanda cathartica as a Potential Candidate for the Treatment of Type 2 Diabetes mellitus. *J. Comput. Biophys. Chem.* 23, 901–923. doi: 10.1142/S2737416524500194  Tomar, R., Mishra, S. S., Sahoo, J., and Rath, S. K. (2024). In Silico and In Vitro Investigation of Phytoconstituents from Flacourtia Jangomas as a Potential Candidate for the Treatment of Type 2 Diabetes Mellitus. *ChemistrySelect* 9. doi: 10.1002/slct.202400542  Viraragavan, A., Hlengwa, N., de Beer, D., Riedel, S., Miller, N., Bowles, S., et al. (2020). Model development for predicting in vitro bio-capacity of green rooibos extract based on composition for application as screening tool in quality control. *Food Funct.* 11, 3084–3094. doi: 10.1039/C9FO02480H  Wang, H., Huang, M., Bei, W., Yang, Y., Song, L., Zhang, D., et al. (2021). FTZ attenuates liver steatosis and fibrosis in the minipigs with type 2 diabetes by regulating the AMPK signaling pathway. *Biomed. Pharmacother.* 138, 111532. doi: 10.1016/j.biopha.2021.111532  Wu, Q.-H., Li, B.-T., Zhu, S.-L., Xiao, X., Zhang, X.-Q., and Tu, J. (2020). [Research on network pharmacology of Mongolian medicine Cymbaria in treatment of type 2 diabetes]. *Zhongguo Zhong Yao Za Zhi* 45, 1764–1771. doi: 10.19540/j.cnki.cjcmm.20191213.401  Yao, Y., Chen, Y., Chen, H., Pan, X., Li, X., Liu, W., et al. (2024). Black mulberry extract inhibits hepatic adipogenesis through AMPK/mTOR signaling pathway in T2DM mice. *J. Ethnopharmacol.* 319, 117216. doi: 10.1016/j.jep.2023.117216  Zou, J., Song, Q., Shaw, P. C., Wu, Y., Zuo, Z., and Yu, R. (2024). Tangerine Peel-Derived Exosome-Like Nanovesicles Alleviate Hepatic Steatosis Induced by Type 2 Diabetes: Evidenced by Regulating Lipid Metabolism and Intestinal Microflora. *Int. J. Nanomedicine* Volume 19, 10023–10043. doi: 10.2147/IJN.S478589 |
| Sirtuin modulation | Azminah, A., Erlina, L., Radji, M., Mun’im, A., Syahdi, R. R., and Yanuar, A. (2019). In silico and in vitro identification of candidate SIRT1 activators from Indonesian medicinal plants compounds database. *Comput. Biol. Chem.* 83, 107096. doi: 10.1016/j.compbiolchem.2019.107096  El Azab, E. F., Alakilli, S. Y. M., Saleh, A. M., Alhassan, H. H., Alanazi, H. H., Ghanem, H. B., et al. (2023). Actinidia deliciosa Extract as a Promising Supplemental Agent for Hepatic and Renal Complication-Associated Type 2 Diabetes (In Vivo and In Silico-Based Studies). *Int. J. Mol. Sci.* 24, 13759. doi: 10.3390/ijms241813759  Feng, B., Huang, B., Jing, Y., Shen, S., Feng, W., Wang, W., et al. (2021). Silymarin ameliorates the disordered glucose metabolism of mice with diet-induced obesity by activating the hepatic SIRT1 pathway. *Cell. Signal.* 84, 110023. doi: 10.1016/j.cellsig.2021.110023  Singh, P., Singh, V. K., and Singh, A. K. (2019). Molecular docking analysis of candidate compounds derived from medicinal plants with type 2 diabetes mellitus targets. *Bioinformation* 15, 179–188. doi: 10.6026/97320630015179 |
| Others | Abdullah, A., Biswas, P., Sahabuddin, M., Mubasharah, A., Khan, D. A., Hossain, A., et al. (2023). Molecular Dynamics Simulation and Pharmacoinformatic Integrated Analysis of Bioactive Phytochemicals from Azadirachta indica (Neem) to Treat Diabetes Mellitus. *J. Chem.* 2023, 1–19. doi: 10.1155/2023/4170703  Alshaghdali, K., Alharazi, T., Rezgui, R., Acar, T., Aljerwan, R. F., Altayyar, A., et al. (2024). Identification and evaluation of putative type 2 diabetes mellitus inhibitors derived from Cichorium intybus. *J. Mol. Struct.* 1306, 137629. doi: 10.1016/j.molstruc.2024.137629  Alvarado, A. T., Muñoz, A. M., Tasayco-Yataco, N., Gamarra-Castillo, F., Ybañez-Julca, R. O., Bendezú, M. R., et al. (2023). In vitro antioxidant and in vivo hypoglycemic activity of biophenols and polyunsaturated fatty acids from Vitis vinifera L. muscat and quebranta seeds from the Valley of Ica-Peru. *Pharmacia* 70, 733–744. doi: 10.3897/pharmacia.70.e109129  Ambalavanan, R., John, A. D., and Selvaraj, A. D. (2020). Nano‐encapsulated Tinospora cordifolia (Willd.) using poly (D, L‐lactide) nanoparticles educe effective control in streptozotocin‐induced type 2 diabetic rats. *IET Nanobiotechnology* 14, 803–808. doi: 10.1049/iet-nbt.2020.0085  Arafa, E.-S. A., Hassan, W., Murtaza, G., and Buabeid, M. A. (2020). Ficus carica and Sizigium cumini Regulate Glucose and Lipid Parameters in High-Fat Diet and Streptozocin-Induced Rats. *J. Diabetes Res.* 2020, 1–9. doi: 10.1155/2020/6745873  Bading-Taïka, B., Souza, A., Bourobou Bourobou, H.-P., and Lione, L. A. (2021). Hypoglycaemic and anti-hyperglycaemic activity of Tabernanthe iboga aqueous extract in fructose-fed streptozotocin type 2 diabetic rats. *Adv. Tradit. Med.* 21, 281–295. doi: 10.1007/s13596-020-00484-0  Ban, Q., Cheng, J., Sun, X., Jiang, Y., and Guo, M. (2020). Effect of feeding type 2 diabetes mellitus rats with synbiotic yogurt sweetened with monk fruit extract on serum lipid levels and hepatic AMPK (5′ adenosine monophosphate-activated protein kinase) signaling pathway. *Food Funct.* 11, 7696–7706. doi: 10.1039/D0FO01860K  Bhavsar, D., Kutre, S., Shikhare, P., Kumar, S., Behera, S. K., and Chauthe, S. K. (2025). Pharmacoinformatics approach for type 2 diabetes mellitus therapeutics using phytocompounds from Costus genus: an in-silico investigation. *J. Biomol. Struct. Dyn.* 43, 7509–7525. doi: 10.1080/07391102.2024.2330712  Binh, N. D. T., Ngoc, N. T. Le, Oladapo, I. J., Son, C. H., Thao, D. T., Trang, D. T. X., et al. (2020). Cyclodextrin glycosyltransferase‐treated germinated brown rice flour improves the cytotoxic capacity of HepG2 cell and has a positive effect on type‐2 diabetic mice. *J. Food Biochem.* 44. doi: 10.1111/jfbc.13533  Boutahiri, S., Bouhrim, M., Abidi, C., Mechchate, H., Alqahtani, A. S., Noman, O. M., et al. (2021). Antihyperglycemic Effect of Lavandula pedunculata: In Vivo, In Vitro and Ex Vivo Approaches. *Pharmaceutics* 13, 2019. doi: 10.3390/pharmaceutics13122019  Buabeid, M. A., Arafa, E.-S. A., Hassan, W., and Murtaza, G. (2020). In Silico Prediction of the Mode of Action of Viola odorata in Diabetes. *Biomed Res. Int.* 2020. doi: 10.1155/2020/2768403  Cam, M. E., Hazar-Yavuz, A. N., Yildiz, S., Ertas, B., Ayaz Adakul, B., Taskin, T., et al. (2019). The methanolic extract of Thymus praecox subsp. skorpilii var. skorpilii restores glucose homeostasis, ameliorates insulin resistance and improves pancreatic β-cell function on streptozotocin/nicotinamide-induced type 2 diabetic rats. *J. Ethnopharmacol.* 231, 29–38. doi: 10.1016/j.jep.2018.10.028  Chakraborty, S., Bhattacharjee, P., Chatterjee, N., and Pal, T. K. (2022). PEGylated Nanoencapsulate of Melatonin-Rich Supercritical CO 2 Extract of Yellow Mustard Seeds is an Authentic Lead for Type 2 Diabetes and Cholesterol Management. *J. Biol. Act. Prod. from Nat.* 12, 173–189. doi: 10.1080/22311866.2022.2029563  Chaudry, S. N., Hussain, W., and Rasool, N. (2021). Analyzing Phytochemicals as Inhibitors of Diabetes Mellitus 2 Causing Proteins based on Computer-Aided Drug Discovery Protocols. *Nat. Prod. J.* 11, 383–391. doi: 10.2174/2210315510999200420113754  Chen, J., Li, P., Ye, S., Li, W., Li, M., and Ding, Y. (2022). Systems pharmacology‐based drug discovery and active mechanism of phlorotannins for type 2 diabetes mellitus by integrating network pharmacology and experimental evaluation. *J. Food Biochem.* 46. doi: 10.1111/jfbc.14492  Chen, L., Lin, X., Fan, X., Lv, Q., Fang, H., Chenchen, Y., et al. (2020). A self-emulsifying formulation of Sonchus oleraceus Linn for an improved anti-diabetic effect in vivo. *Food Funct.* 11, 1225–1229. doi: 10.1039/C9FO00772E  Damián-Medina, K., Salinas-Moreno, Y., Milenkovic, D., Figueroa-Yáñez, L., Marino-Marmolejo, E., Higuera-Ciapara, I., et al. (2020). In silico analysis of antidiabetic potential of phenolic compounds from blue corn (Zea mays L.) and black bean (Phaseolus vulgaris L.). *Heliyon* 6, e03632. doi: 10.1016/j.heliyon.2020.e03632  Davella, R., and Mamidala, E. (2021). Luteolin: A Potential Multiple Targeted Drug Effectively Inhibits Diabetes Mellitus Protein Targets. *J. Pharm. Res. Int.*, 161–171. doi: 10.9734/jpri/2021/v33i44B32661  Dhanya, R., and Jayamurthy, P. (2020). In vitro evaluation of antidiabetic potential of hesperidin and its aglycone hesperetin under oxidative stress in skeletal muscle cell line. *Cell Biochem. Funct.* 38, 419–427. doi: 10.1002/cbf.3478  Di, S., Han, L., An, X., Kong, R., Gao, Z., Yang, Y., et al. (2021). In silico network pharmacology and in vivo analysis of berberine-related mechanisms against type 2 diabetes mellitus and its complications. *J. Ethnopharmacol.* 276, 114180. doi: 10.1016/j.jep.2021.114180  Escobar-Chaves, E., Acin, S., Muñoz, D. L., Fernández, M., Echeverri, A., Echeverri, F., et al. (2023). Polymeric nanoformulation prototype based on a natural extract for the potential treatment of type 2 diabetes mellitus. *J. Drug Deliv. Sci. Technol.* 81, 104264. doi: 10.1016/j.jddst.2023.104264  Fatimawali, Tallei, T. E., Kepel, B. J., Bodhi, W., Manampiring, A. E., and Nainu, F. (2023). Molecular Insight into the Pharmacological Potential of Clerodendrum minahassae Leaf Extract for Type-2 Diabetes Management Using the Network Pharmacology Approach. *Medicina (B. Aires).* 59, 1899. doi: 10.3390/medicina59111899  Gülmez, G., Şen, A., Şekerler, T., Algül, F. K., Çilingir‐Kaya, Ö. T., and Şener, A. (2022). The antioxidant, anti‐inflammatory, and antiplatelet effects of Ribes rubrum L. fruit extract in the diabetic rats. *J. Food Biochem.* 46. doi: 10.1111/jfbc.14124  H.R, D. M. F. ; A. A. I. ; M. (2020). Antidiabetic Effect of Pumpkin Seeds and Gum Arabic and/or Vildagliptin on type 2 Induced Diabetes in Male Rats. *Int. J. Vet. Sci.*, 229–233. doi: 10.37422/IJVS/20.028  Hajji, H., Tabti, K., En-nahli, F., Bouamrane, S., Lakhlifi, T., Ajana, M., et al. (2022). In Silico Investigation on the Beneficial Effects of Medicinal Plants on Diabetes and Obesity: Molecular Docking, Molecular Dynamic Simulations, and ADMET Studies. *Biointerface Res. Appl. Chem.* 11, 6933–6949. doi: 10.33263/BRIAC115.69336949  Hannan, J., Nipa, N., Toma, F. T., Talukder, A., and Ansari, P. (2023). Acute Anti-Hyperglycaemic Activity of Five Traditional Medicinal Plants in High Fat Diet Induced Obese Rats. *Front. Biosci.* 15. doi: 10.31083/j.fbs1502005  Husain, A., Alouffi, S., Khanam, A., Akasha, R., Farooqui, A., and Ahmad, S. (2022). Therapeutic Efficacy of Natural Product ‘C-Phycocyanin’ in Alleviating Streptozotocin-Induced Diabetes via the Inhibition of Glycation Reaction in Rats. *Int. J. Mol. Sci.* 23, 14235. doi: 10.3390/ijms232214235  Ibrahim, M. J., Nangia, A., Das, S., Verma, T., Rajeswari, V. D., Venkatraman, G., et al. (2024). Exploring Holy Basil’s Bioactive Compounds for T2DM Treatment: Docking and Molecular Dynamics Simulations with Human Omentin-1. *Cell Biochem. Biophys.* 83, 793–810. doi: 10.1007/s12013-024-01511-6  Karim, N., Rahman, M. A., Changlek, S., and Tangpong, J. (2020). Short-Time Administration of Xanthone From Garcinia mangostana Fruit Pericarp Attenuates the Hepatotoxicity and Renotoxicity of Type II Diabetes Mice. *J. Am. Coll. Nutr.* 39, 501–510. doi: 10.1080/07315724.2019.1696251  Khalil, H. E., Abdelwahab, M. F., Ibrahim, H.-I. M., AlYahya, K. A., Mohamed, A. A., Radwan, A. S., et al. (2022). Mechanistic Insights into the Ameliorative Effect of Cichoriin on Diabetic Rats—Assisted with an In Silico Approach. *Molecules* 27, 7192. doi: 10.3390/molecules27217192  Khanal, P., and Patil, B. M. (2021). Integration of network and experimental pharmacology to decipher the antidiabetic action of Duranta repens L. *J. Integr. Med.* 19, 66–77. doi: 10.1016/j.joim.2020.10.003  Kuranov, S., Luzina, O., Khvostov, M., Baev, D., Kuznetsova, D., Zhukova, N., et al. (2020). Bornyl Derivatives of p-(Benzyloxy)Phenylpropionic Acid: In Vivo Evaluation of Antidiabetic Activity. *Pharmaceuticals* 13, 404. doi: 10.3390/ph13110404  Laaroussi, H., Bakour, M., Ousaaid, D., Aboulghazi, A., Ferreira-Santos, P., Genisheva, Z., et al. (2020). Effect of antioxidant-rich propolis and bee pollen extracts against D-glucose induced type 2 diabetes in rats. *Food Res. Int.* 138, 109802. doi: 10.1016/j.foodres.2020.109802  Liang, H., Zhang, R., Zhou, L., Wu, X., Chen, J., Li, X., et al. (2024). Corn stigma ameliorates hyperglycemia in zebrafish and GK rats of type 2 diabetes. *J. Ethnopharmacol.* 325, 117746. doi: 10.1016/j.jep.2024.117746  Liao, C., Bao, M., Hasi, Q., Chen, Z., Qian, J., and Baigude, H. (2022). The study of ethanol extract of Epilobium angustifolium L. on blood sugar level in type II diabetic rats. *Pak. J. Pharm. Sci.* 35, 425–433. doi: 10.36721/PJPS.2022.35.2.REG.425-433.1  Liu, L., Guan, F., Chen, Y., Wang, F., Chen, P., Yin, M., et al. (2022). Two Novel Sesquiterpenoid Glycosides from the Rhizomes of Atractylodes lancea. *Molecules* 27, 5753. doi: 10.3390/molecules27185753  Liu, X., Li, Q., Cheng, X., Liu, Z., Zhao, X., Zhang, S., et al. (2020). Oligomannuronate prevents mitochondrial dysfunction induced by IAPP in RINm5F islet cells by inhibition of JNK activation and cell apoptosis. *Chin. Med.* 15, 27. doi: 10.1186/s13020-020-00310-4  Liza, Hussain, G., Malik, A., Akhtar, S., and Anwar, H. (2024). Artemisia vulgaris Extract as a Novel Therapeutic Approach for Reversing Diabetic Cardiomyopathy in a Rat Model. *Pharmaceuticals* 17, 1046. doi: 10.3390/ph17081046  Mariammal, B. G. V., Devarajan, D. W., Jerrin, R., Viswanathan, S., Siddikuzzaman, and Gopal, R. (2021). In Vivo Treatment Efficacy of Essential Oil Isolated from Seeds of Momordica charantia in Streptozotocin-Induced Diabetes Mellitus. *Recent Pat. Biotechnol.* 15, 316–331. doi: 10.2174/1872208315666210910092105  Monteiro-Alfredo, T., Oliveira, S., Amaro, A., Rosendo-Silva, D., Antunes, K., Pires, A. S., et al. (2021). Hypoglycaemic and Antioxidant Properties of Acrocomia aculeata (Jacq.) Lodd Ex Mart. Extract Are Associated with Better Vascular Function of Type 2 Diabetic Rats. *Nutrients* 13, 2856. doi: 10.3390/nu13082856  Moreira, L. N., Santos, J. L. dos, Souza, L. M. V., Marçal, A. C., Dias, A. S., Araújo, S. S. de, et al. (2021). Antioxidant activity and hypoglycemic effect assessment of the leaves from Syzygium cumini (L.) Skeels in Wistar rats. *Acta Sci. Heal. Sci.* 43, e52931. doi: 10.4025/actascihealthsci.v43i1.52931  Mushtaq, W., Ishtiaq, M., Maqbool, M., Ajaib, M., Hussain, T., Waqas Mazhar, M., et al. (2024). Exploration of antidiabetic potential of traditional ethno-medicinal plant Viscum cruciatum Sieber ex Boiss. (Loranthaceae) from Rawalakot district, Poonch, Azad Jammu and Kashmir, Pakistan. *Pakistan J. Bot.* 56. doi: 10.30848/PJB2024-6(15)  Nakashima, Y., Yamamoto, N., Tsukioka, R., Sugawa, H., Ohshima, R., Aoki, K., et al. (2022). In vitro evaluation of the anti-diabetic potential of soymilk yogurt and identification of inhibitory compounds on the formation of advanced glycation end-products. *Food Biosci.* 50, 102051. doi: 10.1016/j.fbio.2022.102051  Nguyen, X., Vu, D., Nguyen, T., Nguyen, T., and Le, H. (2024). Acute toxicity and antidiabetic effect of the ethyl acetate fraction of Commelina diffusa Burm.f. on the high-fat diet and streptozotocin- induced type 2 diabetic mice. *Pak. J. Pharm. Sci.* 37, 855–861. doi: 10.36721/PJPS.2024.37.4.REG.855-861.1  Olasehinde, O. R., Acho, M. A., Afolabi, O. B., and Arise, R. O. (2023). Peptide Hydrolysate of Telfairia occidentalis Hook f. Seed Protein Promotes Effective Glucose Homeostasis by Improving β-cell Dysfunction and Abating Carbohydrate Metabolic Disturbance in Diabetic Rats. *J. Food Biochem.* 2023, 1–14. doi: 10.1155/2023/6652466  Oyebode, O. A., Erukainure, O. L., Sanni, O., and Islam, M. S. (2022). Crassocephalum rubens (Juss. Ex Jacq.) S. Moore improves pancreatic histology, insulin secretion, liver and kidney functions and ameliorates oxidative stress in fructose-streptozotocin induced type 2 diabetic rats. *Drug Chem. Toxicol.* 45, 481–490. doi: 10.1080/01480545.2020.1716783  Parvathi, N., Rajendran, R., Iyyam Pillai, S., and Pillai Subramanian, S. (2024). In Silico Studies on the Antidiabetic activity of Avicularin. *Res. J. Pharm. Technol.*, 19–24. doi: 10.52711/0974-360X.2024.00004  Passari, A. K., Leo, V. V., Singh, G., Samanta, L., Ram, H., Siddaiah, C. N., et al. (2020). In Vivo Studies of Inoculated Plants and In Vitro Studies Utilizing Methanolic Extracts of Endophytic Streptomyces sp. Strain DBT34 Obtained from Mirabilis jalapa L. Exhibit ROS-Scavenging and Other Bioactive Properties. *Int. J. Mol. Sci.* 21, 7364. doi: 10.3390/ijms21197364  Pereira, A. S. P., den Haan, H., Peña-García, J., Moreno, M. M., Pérez-Sánchez, H., and Apostolides, Z. (2019). Exploring African Medicinal Plants for Potential Anti-Diabetic Compounds with the DIA-DB Inverse Virtual Screening Web Server. *Molecules* 24, 2002. doi: 10.3390/molecules24102002  Povydysh, M. N., Titova, M. V., Ivanov, I. M., Klushin, A. G., Kochkin, D. V., Galishev, B. A., et al. (2021). Effect of Phytopreparations Based on Bioreactor-Grown Cell Biomass of Dioscorea deltoidea, Tribulus terrestris and Panax japonicus on Carbohydrate and Lipid Metabolism in Type 2 Diabetes Mellitus. *Nutrients* 13, 3811. doi: 10.3390/nu13113811  Ralte, L., Sailo, H., Kumar, N. S., and Singh, Y. T. (2024). Exploring the pharmacological potential of Lepionurus sylvestris blume: from folklore medicinal usage to modern drug development strategies using in vitro and in silico analyses. *BMC Complement. Med. Ther.* 24, 289. doi: 10.1186/s12906-024-04567-2  Retnaningtyas, E., Setiawan, A., Susatia, B., Hariyanto, T., and Sudiwati, N. L. P. E. (2024). In silico studies of Ruellia tuberosa L. compounds as aldose reductase, dipeptidyl peptidase 4, and α-glucosidase inhibitors against type 2 diabetes mellitus. *J. Pharm. Pharmacogn. Res.* 12, 735–747. doi: 10.56499/jppres23.1891_12.4.735  Rout, D., Chandra Dash, U., Kanhar, S., Swain, S. K., and Sahoo, A. K. (2020). The modulatory role of prime identified compounds in the bioactive fraction of Homalium zeylanicum in high-fat diet fed-streptozotocin-induced type 2 diabetic rats. *J. Ethnopharmacol.* 260, 113099. doi: 10.1016/j.jep.2020.113099  Simeonova, R., Shkondrov, A., Kozuharova, E., Ionkova, I., and Krasteva, I. (2022). A Study on the Safety and Effects of Amorpha fruticosa Fruit Extract on Spontaneously Hypertensive Rats with Induced Type 2 Diabetes. *Curr. Issues Mol. Biol.* 44, 2583–2592. doi: 10.3390/cimb44060176  Singh, P., Ishteyaque, S., Prajapati, R., Yadav, K. S., Singh, R., Kumar, A., et al. (2022). Assessment of antidiabetic effect of 4-HIL in type 2 diabetic and healthy Sprague Dawley rats. *Hum. Exp. Toxicol.* 41. doi: 10.1177/09603271211061873  Sri Prakash, S. R., Kamalnath, S. M., Antonisamy, A. J., Marimuthu, S., and Malayandi, S. (2023). In Silico Molecular Docking of Phytochemicals for Type 2 Diabetes Mellitus Therapy: A Network Pharmacology Approach. *Int. J. Mol. Cell. Med.* 12, 372–387. doi: 10.22088/IJMCM.BUMS.12.4.372  Stojchevski, R., Velichkovikj, S., Bogdanov, J., Hadzi-Petrushev, N., Mladenov, M., Poretsky, L., et al. (2024). Monocarbonyl analogs of curcumin C66 and B2BrBC modulate oxidative stress, JNK activity, and pancreatic gene expression in rats with streptozotocin-induced diabetes. *Biochem. Pharmacol.* 229, 116491. doi: 10.1016/j.bcp.2024.116491  Syaifie, P. H., Harisna, A. H., Nasution, M. A. F., Arda, A. G., Nugroho, D. W., Jauhar, M. M., et al. (2022). Computational Study of Asian Propolis Compounds as Potential Anti-Type 2 Diabetes Mellitus Agents by Using Inverse Virtual Screening with the DIA-DB Web Server, Tanimoto Similarity Analysis, and Molecular Dynamic Simulation. *Molecules* 27, 3972. doi: 10.3390/molecules27133972  Szałabska-Rąpała, K., Zych, M., Borymska, W., Londzin, P., Dudek, S., and Kaczmarczyk-Żebrowska, I. (2024). Beneficial effect of honokiol and magnolol on polyol pathway and oxidative stress parameters in the testes of diabetic rats. *Biomed. Pharmacother.* 172, 116265. doi: 10.1016/j.biopha.2024.116265  T, B. B. ; S. S. S. ; P. M. ; P. K. ; L. A. ; G. (2022). Molecular Docking and TLC Analysis of Candidate Compounds from Lesser used Medicinal Plants Against Diabetes Mellitus Targets. *Jordan J. Biol. Sci.* 15, 339–346. doi: 10.54319/jjbs/150221  Taïlé, J., Bringart, M., Planesse, C., Patché, J., Rondeau, P., Veeren, B., et al. (2022). Antioxidant Polyphenols of Antirhea borbonica Medicinal Plant and Caffeic Acid Reduce Cerebrovascular, Inflammatory and Metabolic Disorders Aggravated by High-Fat Diet-Induced Obesity in a Mouse Model of Stroke. *Antioxidants* 11, 858. doi: 10.3390/antiox11050858  Ul Haq, M. N., Shah, G. M., Gul, A., Foudah, A. I., Alqarni, M. H., Yusufoglu, H. S., et al. (2022). Biogenic Synthesis of Silver Nanoparticles Using Phagnalon niveum and Its In Vivo Anti-Diabetic Effect against Alloxan-Induced Diabetic Wistar Rats. *Nanomaterials* 12, 830. doi: 10.3390/nano12050830  Ullah, S., Rahman, W., Ullah, F., Ullah, A., Jehan, R., Iqbal, M. N., et al. (2024). Identification of lead compound screened from the natural products atlas to treat renal inflammasomes using molecular docking and dynamics simulation. *J. Biomol. Struct. Dyn.* 42, 4851–4861. doi: 10.1080/07391102.2023.2254397  Wang, Y., Guan, Y., Xue, L., Liu, J., Yang, Z., Nie, C., et al. (2021). <scp>l</scp> -Arabinose suppresses gluconeogenesis through modulating AMP-activated protein kinase in metabolic disorder mice. *Food Funct.* 12, 1745–1756. doi: 10.1039/D0FO02163F  Weng, L., Chen, T.-H., Zheng, Q., Weng, W.-H., Huang, L., Lai, D., et al. (2021). Syringaldehyde promoting intestinal motility with suppressing α-amylase hinders starch digestion in diabetic mice. *Biomed. Pharmacother.* 141, 111865. doi: 10.1016/j.biopha.2021.111865  Wihastuti, T. A., Amiruddin, R., Cesa, F. Y., Alkaf, A. I., Setiawan, M., and Heriansyah, T. (2019). Decreasing angiogenesis vasa vasorum through Lp-PLA 2 and H 2 O 2 inhibition by PSP from Ganoderma lucidum in atherosclerosis: in vivo diabetes mellitus type 2. *J. Basic Clin. Physiol. Pharmacol.* 30. doi: 10.1515/jbcpp-2019-0349  Xu, S., Tang, L., Qian, X., Wang, Y., Gong, J., Yang, H., et al. (2022). Molecular mechanism of Ginkgo biloba in treating type 2 diabetes mellitus combined with non‐alcoholic fatty liver disease based on network pharmacology, molecular docking, and experimental evaluations. *J. Food Biochem.* 46. doi: 10.1111/jfbc.14419  Xu, X., Guo, Y., Chen, M., Li, N., Sun, Y., Ren, S., et al. (2024). Hypoglycemic activities of flowers of Xanthoceras sorbifolia and identification of anti-oxidant components by off-line UPLC-QTOF-MS/MS-free radical scavenging detection. *Chinese Herb. Med.* 16, 151–161. doi: 10.1016/j.chmed.2022.11.009  Yan, Z., Wu, H., Zhou, H., Chen, S., He, Y., Zhang, W., et al. (2020). Integrated metabolomics and gut microbiome to the effects and mechanisms of naoxintong capsule on type 2 diabetes in rats. *Sci. Rep.* 10, 10829. doi: 10.1038/s41598-020-67362-2  Yang, J., Bi, Y., Liang, S., Gu, Z., Cheng, L., Li, C., et al. (2020). The in vivo digestibility study of banana flour with high content of resistant starch at different ripening stages. *Food Funct.* 11, 10945–10953. doi: 10.1039/D0FO02494E  Yao, Y., Yan, L., Chen, H., Wu, N., Wang, W., and Wang, D. (2020). Cyclocarya paliurus polysaccharides alleviate type 2 diabetic symptoms by modulating gut microbiota and short-chain fatty acids. *Phytomedicine* 77, 153268. doi: 10.1016/j.phymed.2020.153268  Zanfirescu, A., Avram, I., Gatea, F., Roșca, R., and Vamanu, E. (2023). In Vitro and In Vivo Antihyperglycemic Effects of New Metabiotics from Boletus edulis. *Life* 14, 68. doi: 10.3390/life14010068  Zhang, H., Liu, J., Lv, Y., Jiang, Y., Pan, J., Zhu, Y., et al. (2020). Changes in Intestinal Microbiota of Type 2 Diabetes in Mice in Response to Dietary Supplementation With Instant Tea or Matcha. *Can. J. Diabetes* 44, 44–52. doi: 10.1016/j.jcjd.2019.04.021  Zhu, Y., Wu, L., Zhao, Y., Wang, Z., Lu, J., Yu, Y., et al. (2022). Discovery of oridonin as a novel agonist for BRS-3. *Phytomedicine* 100, 154085. doi: 10.1016/j.phymed.2022.154085 |
